# Ni- and Ni/Pd-Catalyzed Reductive Coupling of Lignin-Derived Aromatics to Access Biobased Plasticizers

**DOI:** 10.1021/acscentsci.2c01324

**Published:** 2023-01-18

**Authors:** Zhi-Ming Su, Jack Twilton, Caroline B. Hoyt, Fei Wang, Lisa Stanley, Heather B. Mayes, Kai Kang, Daniel J. Weix, Gregg T. Beckham, Shannon S. Stahl

**Affiliations:** † Department of Chemistry, University of Wisconsin−Madison, 1101 University Avenue, Madison, Wisconsin 53706, United States; ‡ Renewable Resources and Enabling Sciences Center, National Renewable Energy Laboratory, Golden, Colorado 80401, United States

## Abstract

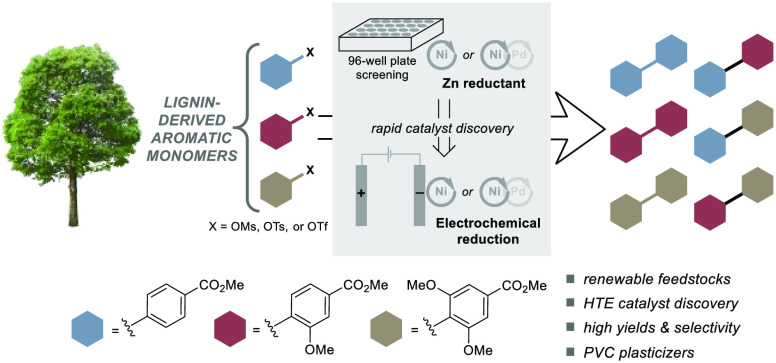

Lignin-derived aromatic chemicals offer a compelling alternative to petrochemical feedstocks, and new applications are the focus of extensive interest. 4-Hydroxybenzoic acid (**H**), vanillic acid (**G**), and syringic acid (**S**) are readily obtained via oxidative depolymerization of hardwood lignin substrates. Here, we explore the use of these compounds to access biaryl dicarboxylate esters that represent biobased, less toxic alternatives to phthalate plasticizers. Chemical and electrochemical methods are developed for catalytic reductive coupling of sulfonate derivatives of **H**, **G**, and **S** to access all possible homo- and cross-coupling products. A conventional NiCl_2_/bipyridine catalyst is able to access the **H–H** and **G–G** products, but new catalysts are identified to afford the more challenging coupling products, including a NiCl_2_/bisphosphine catalyst for **S–S** and a NiCl_2_/phenanthroline/PdCl_2_/phosphine cocatalyst system for **H–G**, **H–S**, and **G–S**. High-throughput experimentation methods with a chemical reductant (Zn powder) are shown to provide an efficient screening platform for identification of new catalysts, while electrochemical methods can access improved yields and/or facilitate implementation on larger scale. Plasticizer tests are performed with poly(vinyl chloride), using esters of the 4,4′-biaryl dicarboxylate products. The **H–G** and **G–G** derivatives, in particular, exhibit performance advantages relative to an established petroleum-based phthalate ester plasticizer.

## Abstract 2

HTE catalyst discovery with chemical reductants enables scalable electrochemical methods for Ni- and Ni/Pd-catalyzed reductive coupling of lignin-derived aromatics to generate biobased plasticizers.

## Introduction

**Figure 1:**
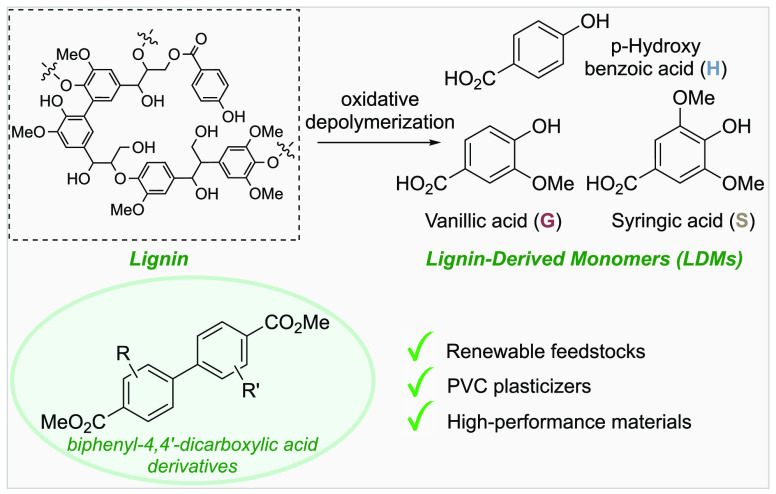
Lignin is an abundant biomass-derived source of aromatics that represent potential precursors to commercially important biphenyl-4,4′-dicarboxylates.

Lignin represents the largest source of biomass-derived aromatic chemicals and is an ideal supplement or alternative to petroleum-based feedstocks.^1−9^ Significant progress has been made in lignin depolymerization into aromatic monomers,^4−9^ but methods for conversion of lignin-derived monomers (LDMs) into value-added chemicals are still in the nascent stages of development.^1−3,10^ In connection with efforts focused on oxidative lignin depolymerization,^11−13^ we recognized that some of the most common products, 4-hydroxybenzoic acid (**H**), vanillic acid (**G**), and syringic acid (**S**), could serve as precursors to biaryl dicarboxylates (Figure 1).^14^ The parent analogue, biphenyl-4,4′-dicarboxylic acid (BPDA), has been the focus of commercial interest as a monomer for polyesters and as the core structure for nonphthalate plasticizers for poly(vinyl chloride) (PVC).^15−18^ Existing methods for the synthesis of BPDA use petroleum-based precursors in multistep routes (e.g., involving oxidative coupling, alkylation, and/or dehydrogenation steps, paired with autoxidation of alkyl groups into carboxylic acids), and they often afford a mixture of regioisomers.^16,19−21^ Reductive coupling of phenol derivatives represents a different route to BPDA derivatives that accesses a single product regioisomer. The biomass-derived **H** compound provides a means to access the same BPDA analogue currently sourced from petroleum, while the **G** and **S** compounds that have methoxy substituents will afford BPDA derivatives that could have favorable properties (e.g., as a PVC plasticizer).

**Scheme 1:**
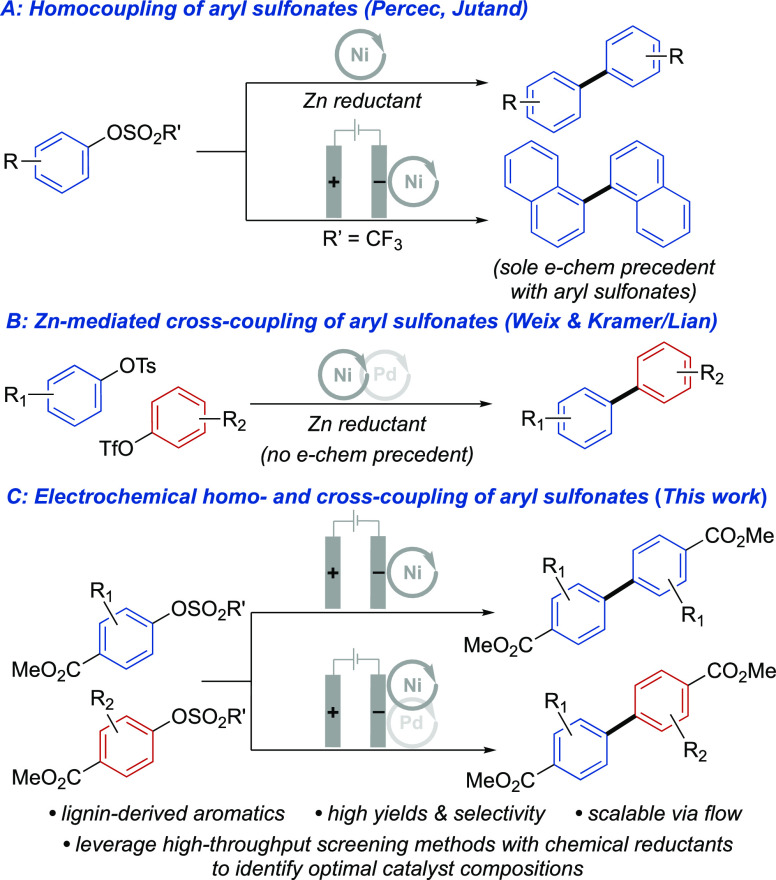
Precedents Relevant to Reductive Coupling of Lignin-Derived Aryl Sulfonates

We postulated that the **H**, **G**, and **S** products of lignin depolymerization could be readily converted to aryl sulfonates amenable to reductive cross-coupling. Ni-catalyzed coupling of aryl electrophiles to access biaryls was first reported in the 1970s, and the field advanced significantly in subsequent decades.^22−31^ These reactions typically feature stoichiometric metal reductants, such as Zn powder, but important electrochemical precedents also exist. Several examples provide an important foundation for the present work. In 1995, Percec et al. demonstrated that a Ni/PPh_3_ catalyst system with Zn reductant promotes homocoupling of aryl sulfonates to biaryls (Scheme 1A).^32^ Shortly thereafter, Jutand and co-workers achieved homocoupling of aryl triflates with phosphine-ligated Pd *or* Ni catalysts. This study included a single example of electrochemical Ni-catalyzed homocoupling, using 1-naphthyl triflate as the substrate (Scheme 1A).^29,30,33^ In recent years, Weix and co-workers have developed methods for selective cross-coupling of aryl electrophiles with a cocatalyst system containing both Ni and Pd in the presence of Zn as the reductant.^34−37^ The groups of Weix^37^ and Kramer/Lian^38^ independently reported reductive cross-coupling of two different aryl sulfonates by pairing Pd/bisphosphine and Ni/diimine cocatalysts [diimine = substituted 2,2′-bipyridine (bpy) or 1,10-phenanthroline (phen) derivatives] with Zn (Scheme 1B). To date, no electrochemical methods to our knowledge have been reported for reductive cross-coupling of phenol derivatives (Scheme 1C).^39−42^

Chemical and electrochemical conditions have complementary advantages for reductive coupling reactions. Chemical conditions are more straightforward to implement on small scale, owing to their use of standard laboratory equipment, and they are more amenable to high-throughput experimentation (HTE) techniques for catalyst discovery and reaction optimization. Electrochemical methods offer advantages for large scale applications by avoiding the challenges of handling dense metal-powder reagents and creating opportunities to improve sustainability. Although advances have been made in the development of electrochemical reactors for parallel reaction screening,^43,44^ chemical HTE methodology retains substantially improved efficiency and is compatible with smaller quantities of reagents. In this context, we postulated that HTE screening methods using chemical reductants could enable rapid identification of promising catalyst systems and conditions for subsequent development of electrochemical methods. The results outlined below validate this hypothesis and achieve successful chemical and electrochemical conditions for all possible homo- and cross-coupling permutations between **H**-, **G**-, and **S**-derived reaction partners. Additional important outcomes of this study include (a) identification of mono- and bidentate phosphine ligands that lack precedent in Ni-catalyzed reductive coupling reactions, (b) successful adaptation of catalysts from chemical to electrochemical conditions, with matching or superior performance, (c) the first demonstration of Ni/Pd cocatalyzed reductive biaryl cross-coupling under electrochemical conditions, and (d) data showing that biaryl dicarboxylic esters prepared from LDMs exhibit improved PVC plasticizer performance and reduced toxicity relative to a commercial phthalate-based plasticizer.^45^

## Results and Discussion

### Ni-Catalyzed Homocoupling of LDMs

The methyl esters of **H**, **G**, and **S** are readily converted into electrophiles by reaction of the phenols with sulfonyl chlorides, RSO_2_Cl [R = methyl (Ms) or tosyl (Ts)], or triflic anhydride (Tf_2_O). Initial studies evaluated the electrochemical homocoupling of methyl 3-methoxy-4-((methylsulfonyl)oxy)benzoate (**G–OMs**). The two possible byproducts are denoted as the Ar–H and ArO–H species, derived from reductive cleavage of the C–O or the S–O bond of the **G–OMs** substrate. A combination of NiCl_2_(dme)/bpy has been used previously for reductive homocoupling of Ar–X species^29,30^ and this catalyst system was tested initially in an undivided cell with LiBr as the electrolyte and stainless steel as the anode. However, these conditions only afforded the **G–G** product in 29% yield, with a significant amount of byproduct and unreacted starting material (Table 1, entry 1). Use of increased bpy ligand loading (bpy:Ni = 3:1) stabilizes the catalyst^46^ and leads to a higher yield of the desired product (72%), together with the Ar–H byproduct (27%; Table 1, entry 2). Other sacrificial anodes were tested in an effort to optimize the yield of biaryl product (Table 1, entries 3–5). Significant reductive C–O cleavage was also observed when Al or Zn was used as the anode (Table 1, entries 3 and 4). This C–O cleavage is rationalized by previous observations that aryl-Ni species can transfer an aryl group to Zn^2+^, generating aryl-Zn species that are susceptible to protonolysis and Ar–H byproduct formation.^37,47^ Electrolysis in an undivided cell using a Mg anode proved ineffective (Table 1, entry 5). In this case, reductive S–O bond cleavage was favored, likely reflecting single-electron reduction of the sulfonyl group at the Mg surface.^33^ These considerations prompted us to test a sacrificial anode with a divided cell configuration that would avoid the contact of substrate with the anode surface and minimize the presence of Lewis acidic metal ions in the cathodic chamber. This hypothesis was validated by observation of a 92% **G–G** product yield when using a Mg anode in a divided cell (Table 1, entry 7). This outcome is noteworthy because it is significantly better than that achieved when performing the same reaction under previously reported chemical conditions^32^ or optimized variations thereof with Zn powder as the reductant (48% and 59% **G–G** yields, respectively; Table S1). Use of analogous conditions with **H–OMs** as the substrate leads to near-quantitative yield of the biaryl **H–H** product (Table 1, entry 8). This outcome was achieved, even when lowering the Ni catalyst loading to 1 mol %. Use of a stainless-steel anode in an undivided cell retained good yield (Table 1, entry 9). The latter conditions are readily implemented in a recirculating flow electrolysis cell with a parallel-plate reactor. This approach was used to conduct a larger scale reaction (11 g, 48 mmol **H–OMs**), accessing the **H–H** product in 80% yield with 2 mol % Ni catalyst (see Section 4 of the Supporting Information for details).

**Table 1:**
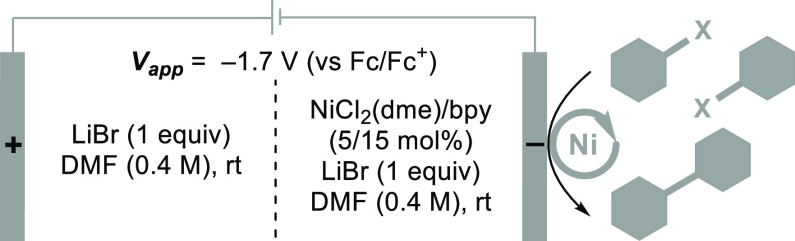

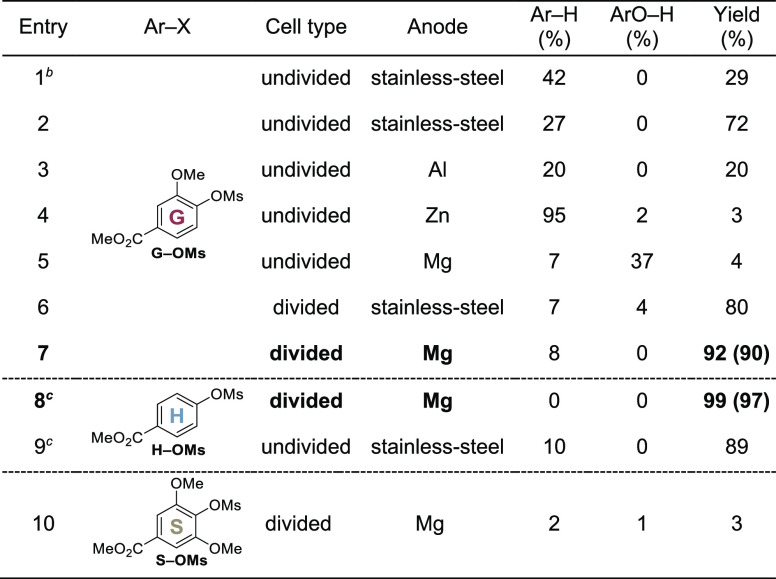
Optimization of Electrochemical Ni-Catalyzed Reductive Homocoupling^a^

a See the Supporting Information for full experimental details. Yields are determined by ^1^H NMR analysis of the crude reaction mixture using mesitylene as an internal standard; yields shown in parentheses are isolated.

b 5 mol % bpy. The rest of the mass corresponds to unreacted starting material.

c 1 mol % Ni catalyst.

**Figure 2:**
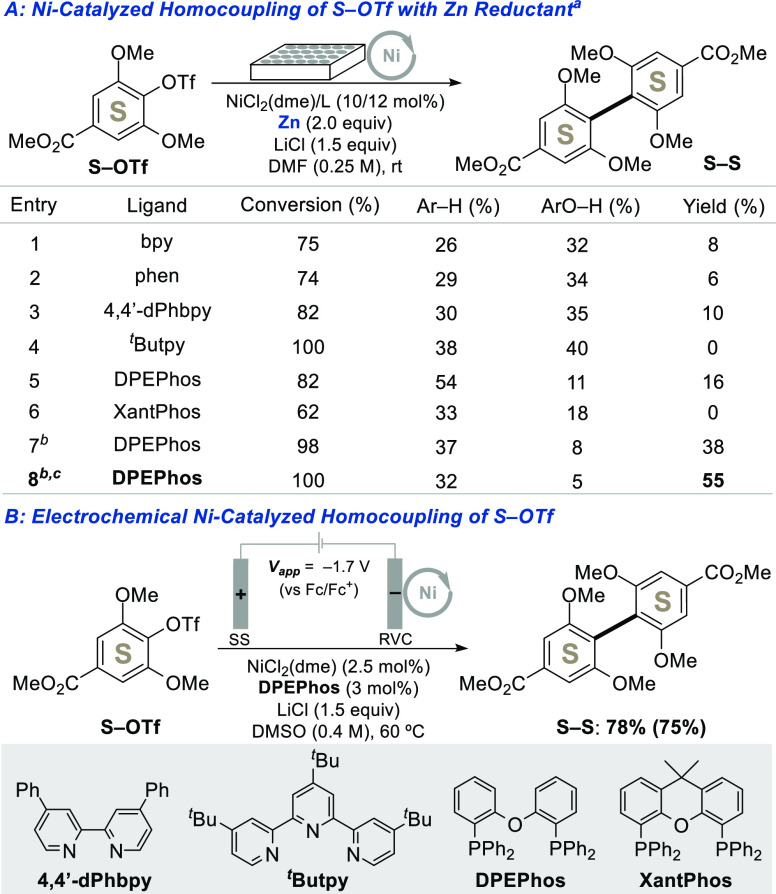
Ni-catalyzed reductive homocoupling of **S–OTf**: translating conditions optimized with Zn reductant (A) to electrochemical conditions (B). See the Supporting Information for full experimental details. (a) Yields are determined by ^1^H NMR analysis of the crude reaction mixture using mesitylene as an internal standard; yields shown in parentheses are isolated. (b) 60 °C. (c) DMSO solvent.

The catalyst and conditions identified for homocoupling of **H–OMs** and **G–OMs** proved ineffective with the more sterically demanding syringic acid derivative **S–OMs**. Only trace quantities of **S**–**S** product were obtained (Table 1, entry 10). To facilitate evaluation of modified conditions, we used a 24-well screening platform with Zn powder as a chemical reductant. The triflate derivative **S–OTf** was found to be more reactive than the mesylate (Table S2), and this substrate was tested with dozens of nitrogen- and phosphine-based ligands. Selected results are summarized in Figure 2A, with full screening data provided in the Supporting Information (see Tables S2–S8). DPEPhos was the only ligand that showed modest success; even the closely related, conformationally more rigid XantPhos ligand was completely ineffective (Figure 2A, entries 5 and 6). Increasing the temperature to 60 °C led to an increase in conversion and product yield (Figure 2A, entry 7), and changing the solvent to DMSO led to a 55% yield of **S–S** (Figure 2A, entry 8). The outcome improved even further when the conditions were adapted to an undivided electrochemical cell with a stainless-steel anode: the desired dimer **S–S** was generated in 78% yield (Figure 2B; see Table S9 for full screening data). This improved electrochemical outcome was achieved, even though the NiCl_2_/DPEPhos catalyst loading was lowered to 2.5 mol %.

### Optimization of Ni/Pd-Catalyzed Cross-Coupling

**Figure 3:**
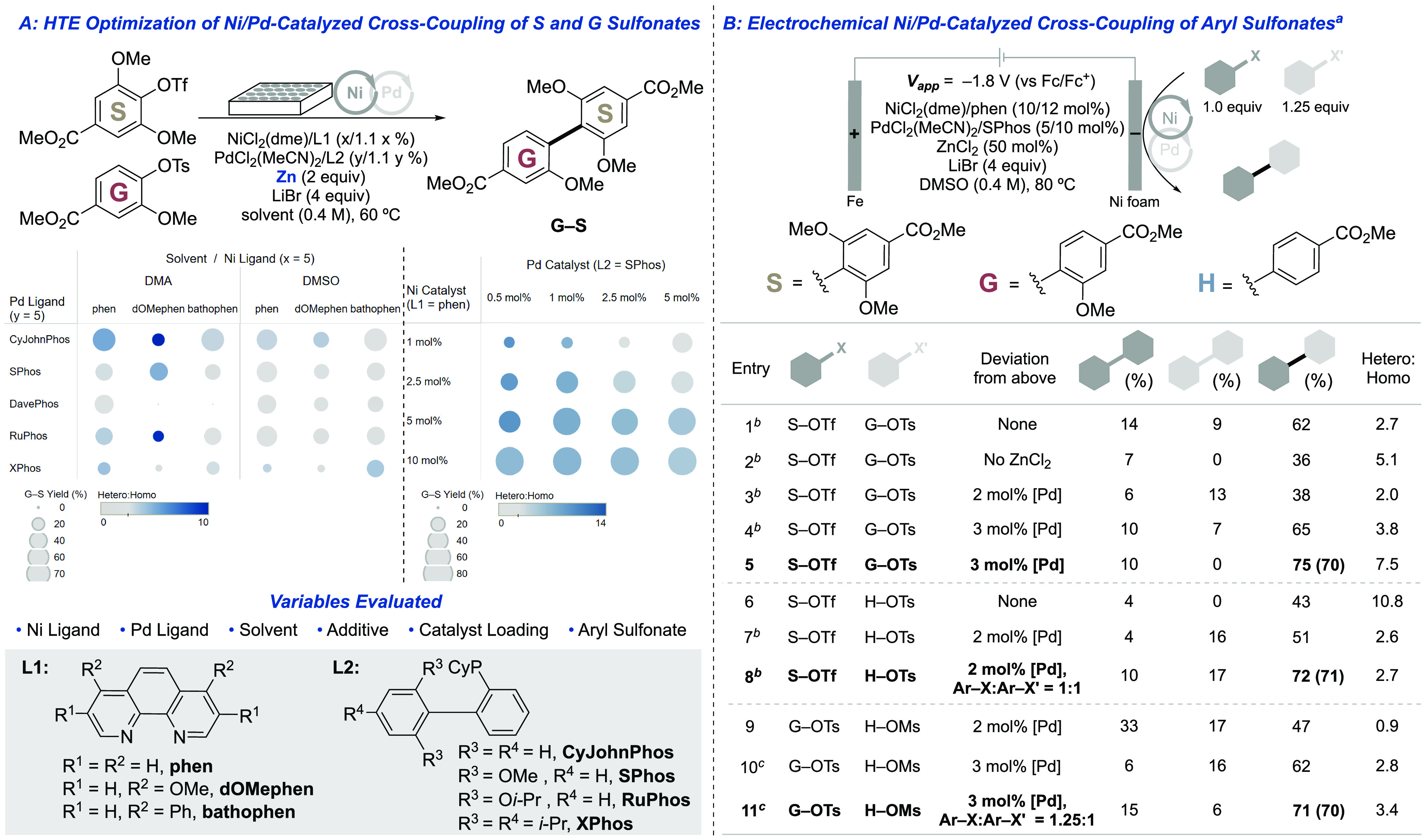
Ni/Pd**-**catalyzed reductive cross-coupling of lignin-derived aryl sulfonates. (A) HTE optimization of G/S cross-coupling. Left chart: S–OTf:G–OTs = 1:1; right chart: DMSO solvent, S–OTf:G–OTs = 1:1.25. The hetero:homo coupling ratio is defined as **G–S** yield/(**G–G** yield + **S–S** yield). (B) Optimization of electrochemical Ni/Pd-catalyzed cross-coupling. See the Supporting Information for full experimental details. (a) Yields determined by UPLC-MS analysis using 1,3,5-trimethoxylbenzene as an internal standard; yields shown in parentheses are isolated. (b) RVC cathode. (c) L1 = 4,4′-dPhbpy, L2 = dppb (3.6 mol %), DMA instead of DMSO, 60 °C.

The Ni-only catalyst systems noted above were evaluated in the cross-coupling of **H**, **G**, and **S** sulfonates; however, these reactions led to poor selectivity and yields of the desired products (Table S10). These complications prompted us to evaluate the recently disclosed dual Ni/Pd cocatalyst systems.^34−38^ For example, the method of Weix and co-workers, which employs Ni/Pd chloride salts in combination with 4,4′-diphenyl-bpy (4,4′-dPhbpy) and 1,4-bis(diphenylphosphino)butane (dppb) and Zn as a chemical reductant, supports cross-coupling of aryl triflates and tosylates.^37^ Efforts to translate this catalyst system to electrochemical cross-coupling of **G** and **S** sulfonates were unsuccessful, regardless of the sulfonate activating groups: biaryl products formed in ≤15% yield and favored the homocoupling products (Figure S5). Consequently, we again elected to use the high-throughput experimentation platform with Zn as the chemical reductant to evaluate modified conditions. Initial studies focused on cross-coupling of **G** and **S** sulfonates, evaluating different combinations of ligands, solvents, additives, sulfonate activating groups, and Ni/Pd ratios, and the results are visualized in Figure 3A (see Tables S11–S14 for full screening). The size of the circles in these charts corresponds to the yield, while the color reflects the hetero:homo coupling ratio (darker blue reflects higher selectivity). Among the most noteworthy outcome from these experiments is the beneficial effect of bulky biaryl dialkyl monophosphine ligands (“Buchwald ligands”^48^). The utility of these ligands could reflect their ability to promote the difficult reductive elimination steps.^48^ CyJohnPhos was the most effective ligand under screening conditions with Zn powder as the reductant (Figure 3A). Subsequent studies revealed that CyJohnPhos decomposes under electrochemical reaction conditions. In contrast, SPhos is stable and supports good reactivity. Further chemical screening evaluated different Ni:Pd ratios in a cocatalyst system derived from NiCl_2_(dme)/phen and PdCl_2_(MeCN)_2_/SPhos (Figure 3A). These studies showed that the highest yields were obtained with 10 mol % Ni and a Pd loading ranging from 0.5 to 5 mol %.

We then initiated electrochemical studies to access cross-coupled products **G–S**, **H–S**, and **H–G**, starting with a cocatalyst composed of 10 mol % NiCl_2_(dme)/phen and 5 mol % PdCl_2_(MeCN)_2_/SPhos (Figure 3B). Promising performance was identified with a reticulated vitreous carbon (RVC) cathode, sacrificial iron anode, and a constant applied potential of −1.8 V vs Fc/Fc^+^. Inclusion of 0.5 equiv of ZnCl_2_ significantly improved the reaction outcome (Figure 3B, entries 1 and 2), consistent with previous evidence that Zn^2+^ salts mediate transmetalation between Ni and Pd centers.^37,49,50^ Increasing the phosphine ligand loading from 1.1 to 2 equiv with respect to Pd stabilized the Pd catalyst. These initial conditions afforded the desired product **G–S** in 62% yield with 23% homocoupled byproducts, similar to the yields obtained in the chemical screening studies with Zn as a chemical reductant. It is not surprising that the reaction selectivity varies somewhat between chemical and electrochemical conditions. One important factor is that the cathode potential will not directly match the reduction potential of Zn, and variations in substrate consumption (i.e., via byproduct formation) will lead to differences in the selectivity between chemical and electrochemical conditions. Also, because the selectivity is dictated by pairing of the Ni and Pd catalytic cycles, different rates of catalyst turnover at the Zn surface (chemical) vs cathode surface (electrochemical) will affect the hetero:homo coupling selectivity. Adjusting the Ni:Pd ratio from 2:1 to 3.3:1 and using a Ni foam cathode instead of RVC increased the **G–S** product yield to 75% (Figure 3B, entries 3–5). Slight modification of these conditions accessed the **H–S** cross-coupling product in 72% yield (Figure 3B, entry 8). Analogous conditions were less effective for cross-coupling of the less sterically demanding **H** and **G** sulfonates (Figure 3B, entry 9), but adaptation of the chemical catalyst system reported by Weix and co-workers proved effective for the cross-coupling of **H–OMs**/**G–OTs**, accessing **H–G** in 71% yield (Figure 3B, entry 11). This reaction represents the first selective cross-coupling (under chemical or electrochemical conditions) of aryl mesylate/aryl tosylate partners, which are significantly more economical than aryl triflates.

### Plasticizer Properties of Lignin-Derived Biaryls

**Figure 4:**
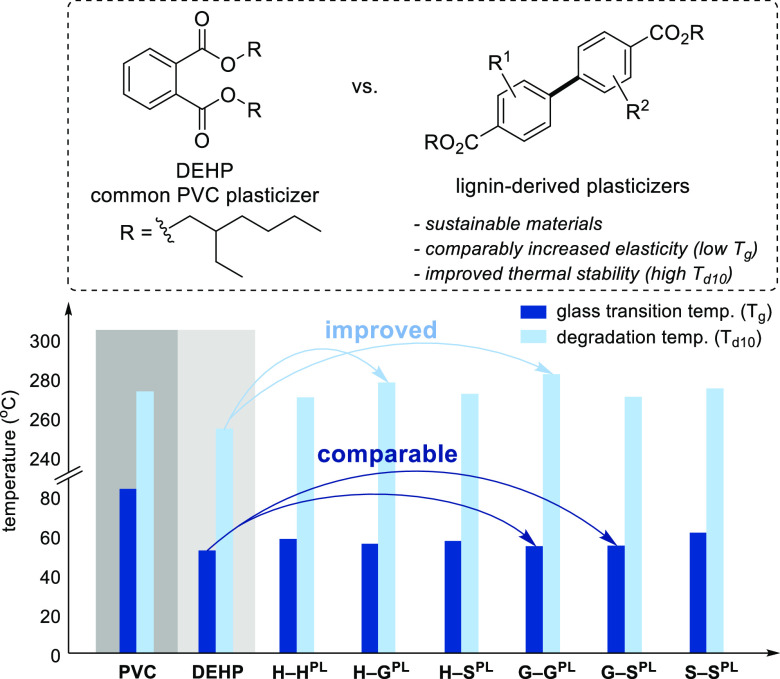
Thermal analysis of lignin-derived biaryl plasticizers. From left to right: unplasticized PVC, 10 wt % plasticized PVC with DEHP, and 10 wt % plasticized PVC with lignin-derived biaryl plasticizers.

The above results provide access to all possible homo- and cross-coupled BPDA derivatives of **H**, **G**, and **S**. These structures provide the basis for testing of these materials as plasticizers for PVC and comparison of their performance relative to the existing petroleum-derived incumbent, di(2-ethylhexyl)phthalate (DEHP). Each of the BPDA methyl esters was subjected to Ti(OBu)_4_-promoted transesterification with 2-ethylhexanol to afford the corresponding DEH–BPDA derivatives, designated **H–H**^**PL**^, **H–G**^**PL**^, **H–S**^**PL**^, **G–G**^**PL**^, **G–S**^**PL**^, and **S–S**^**PL**^. The thermal properties of these structures were characterized by thermogravimetric analysis (TGA) and differential scanning calorimetry (DSC) (Figure S9, Table S15). DEHP and the DEH–BPDA derivatives were then individually integrated with PVC at 10 wt %, and the materials were analyzed by TGA and DSC to measure their glass transition temperature (*T*_g_) and the temperature at which the polymer degrades with 10% or 50% loss of its original weight (*T*_d10_, *T*_d50_) (Figures S10 and S11, Table S16). The former metric reflects the ability of the plasticizer to soften PVC, while the latter metrics reflect the thermostability of the plasticized material. Preferred plasticizers will achieve lower *T*_g_ and higher *T*_d10_/*T*_d50_ values. The results, summarized in Figure 4, show that the different plasticizers lower the *T*_g_ of PVC from 83.0 to 52.1–61.0 °C. The greatest effect is observed with DEHP, **G–G**^**PL**^, and **G–S**^**PL**^, which lead to *T*_g_ values of 52.1, 54.4, and 54.6 °C, respectively. Meanwhile, **H–G**^**PL**^ and **G–G**^**PL**^ show a notable enhancement in thermostability, with these plasticized materials exhibiting even higher *T*_d10_ (278 and 281 °C) than PVC itself (272 °C), and both outperform DEHP (*T*_d10_ = 253 °C).

The same series of compounds were then evaluated using tools developed by the US Environmental Protection Agency to predict their potential toxicity^51^ and their metabolic and environmental transformation^52^ (see Section 7 of the Supporting Information for details). The results assign these materials to the lowest hazard category with respect to acute toxicity to mammals (>5,000 mg/kg), and the lignin-derived BPDAs arising from hydrolysis of the esters are predicted to be metabolized more easily than phthalic acid. Further experimental studies will be needed to validate this assessment, but these results and the promising performance characteristics in Figure 4 reinforce the potential performance-advantaged properties of biobased plasticizers derived from these BPDAs.

## Conclusion

The results above demonstrate the utility of Ni- and Ni/Pd-catalyzed cross-electrophile coupling to convert lignin-derived aromatic compounds into a collective of substituted biphenyl dicarboxylic acids. All possible combinations of **H**, **G**, and **S** monomers have been prepared, with symmetrical dimers accessed using a Ni-only catalyst system and the unsymmetrical dimers accessed using Ni/Pd cocatalyst systems. The results highlight the synergy between chemical and electrochemical reduction methods. HTE screening methods with a chemical reductant offer advantages for identification of effective catalyst compositions. For example, chemical HTE methods identified Ni/DPEPhos catalyst and Ni/phen/Pd/SPhos cocatalyst systems, which lacked precedent for homo- and cross-biaryl coupling, respectively. In each case, the chemical reaction conditions were successfully translated to electrochemical conditions, often resulting in improved performance. The beneficial effect of bulky phosphine ligands with the **S-**derived monomers has important implications for other cross-electrophile coupling reactions with sterically congested aryl electrophiles, beyond those studied here. Finally, the new BPDA derivatives bearing methoxy substituents, which are intrinsic to lignin-based aromatics, exhibit appealing plasticizer properties that merit further investigation and development.

## Supporting Information Available

Complete experimental procedures and compound characterization (PDF)
