# Supplementary material for: Ni- and Ni/Pd-Catalyzed Reductive Coupling of Lignin-Derived Aromatics to Access Biobased Plasticizers
Source: ACS Cent Sci. 2023 Jan 18;9(2):159–65. doi: 10.1021/acscentsci.2c01324 (PMC9951286; doi:10.1021/acscentsci.2c01324)

Supporting Information

Ni- and Ni/Pd-Catalyzed Reductive Coupling of Lignin-Derived Aromatics to Access Bio-Based Plasticizers

Zhi-Ming Su,[1] Jack Twilton,[1] Caroline B. Hoyt,[2] Fei Wang,[1] Lisa Stanley,[2] Heather B. Mayes,[2] Kai Kang,[1] Daniel J. Weix,[1] Gregg T. Beckham,[2*] Shannon S. Stahl[1*]

[1] Department of Chemistry, University of Wisconsin-Madison, 1101 University Avenue, Madison, Wisconsin 53706, United States.

[2] Renewable Resources and Enabling Sciences Center, National Renewable Energy Laboratory, Golden, Colorado 80401, United States.

stahl@chem.wisc.edu; gregg.beckham@nrel.gov

Table of Contents

1. General Experimental Considerations S2

2. General Procedure for Aryl Sulfonate Esters Syntheses (GP 1) S3

3. General Procedures for Bulk Electrolysis S3

4. General Procedures for Flow Electrolysis S5

5. Optimization of Reaction Conditions S7

6. CV Studies S17

7. Plasticizers S19

8. Compound Characterization Data S27

9. References S35

10. NMR Spectra S36

1. *General Experimental Considerations*

Solvents and reagents

All solvents (anhydrous) and reagents were purchased from commercial sources and used as received without further purification. Starting materials were purchased from MilliporeSigma, Alfa Aesar, and Combi-Blocks. Zn powder (average 4-7 micron) was purchased from Alfa Aesar. Nickel salts, palladium salts, and ligands were purchased from MilliporeSigma or Alfa Aesar. Anhydrous solvents were purchased from MilliporeSigma and handled in a nitrogen-filled glove box.

Electrodes

All electrode materials were purchased from commercial sources and used as received. RVC foams (pore size 30 ppi) were purchased from Ultramet. Ni foams (1.6 mm thickness) were purchased from MTI. Fe rods (5 mm diameter) were purchased from American-Scientific. Other metal electrodes (Zn, Mg, Al) were purchased from MilliporeSigma. Glassy carbon working electrodes (MF-2012) and non-aqueous reference electrodes (MF-2062) were purchased from BASi. Pt wires were purchased from MilliporeSigma and custom-made as Pt counter electrodes.

Characterization of products

^{1}H and ^{13}C NMR spectra were recorded on Bruker 400 or 600 MHz spectrometers. Chemical shifts are given in parts per million (ppm) relative to residual solvent peaks in the ^{1}H and ^{13}C NMR spectra or are referenced as noted. The following abbreviations (and their combinations) are used to label the multiplicities: s (singlet), d (doublet), t (triplet), m (multiplet) and br (broad). High-resolution mass spectra were obtained using a Thermo Q ExactiveTM Plus by the mass spectrometry facility at the University of Wisconsin. UPLC-MS analysis was conducted on a Waters-Acquity. Chromatographic purification of products was accomplished by chromatography on silica gel 60 M (particle size 40-63 µm, 230-400 mesh) from MACHEREY-NAGEL Inc. Thin-layer chromatography (TLC) was performed on Silicycle silica gel UV254 pre-coated plates (0.25 mm). Visualization of the developed chromatogram was performed by using UV lamps or $KMnO_4$ stain.

Electrochemical experiments

All cyclic voltammetric (CV) and chronoamperometric (CA) experiments were performed using Nuvant Array PGStats or a Pine WaveNow PGstat. The CV experiments were carried out in a three-electrode cell configuration with a glassy carbon (GC) working electrode (3 mm diameter), and a platinum wire counter electrode (~ 1.0 cm, spiral wire). The working electrode potentials were measured versus $Ag/AgNO_3$ reference electrode (internal solution, 0.1 M Bu_4NPF_6 and 0.01 M $AgNO_3$ in DMF). The redox potential of ferrocene/ferrocenium (Fc/Fc^+) was measured (under same experimental conditions) and used to provide an internal reference. The potential values were then adjusted relative to Fc/Fc^+, and electrochemical studies in organic solvents were recorded accordingly. The GC working electrode was polished with alumina powder (5 µm) before each experiment. All solutions used for CV analysis were prepared 30 min before the experiments and kept under nitrogen atmosphere using a thin Teflon tube to allow continuous nitrogen bubbling. Bulk electrolysis experiments were performed in custom-built undivided or divided cells, with RVC or Ni foam working electrodes, Mg, Fe or stainless-steel counter electrodes and $Ag/AgNO_3$ (internal solution, 0.1 M Bu_4NPF_6 and 0.01 M $AgNO_3$ in DMF) for a reference electrode.

2. *General Procedure for Aryl Sulfonate Esters Syntheses (GP 1)*

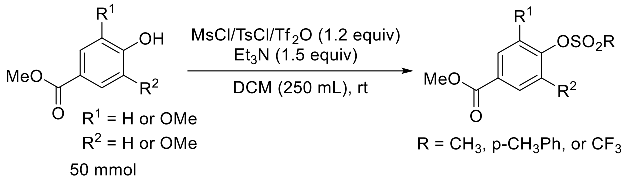

The following procedure was adapted from precedents reported in literatures.[1,2] To a 500 mL round-bottom flask was added the substrate (50 mmol, 1.0 equiv) and anhydrous DCM (200 mL). Et_3N (10.4 mL, 75 mmol, 1.5 equiv) was then injected to the solution. Another 200 mL round-bottom flask was charged with the sulfonating reagent (60 mmol, 1.2 equiv) and anhydrous DCM (50 mL), mixed thoroughly, then this solution was added dropwise to the 500 mL flask. The reaction mixture was stirred at room temperature and stopped when full conversion was observed via TLC. The DCM solution was washed with 300 mL water and 300 mL brine sequentially. The aqueous layers were collected and extracted with 200 mL DCM. The organic layers were combined, dried over anhydrous Na_2SO_4, and concentrated *in vacuo*. The crude product was purified by column chromatography to furnish the desired product (eluent: hexane/ethyl acetate, 2/1).

3. *General Procedures for Bulk Electrolysis*

*Electrochemical reductive homocoupling in a divided cell (**GP 2**)*

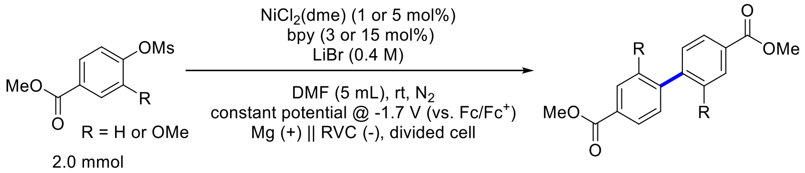

To the cathodic chamber of the divided cell (Figure S1) was added $NiCl_2$(dme) (4.4 or 22 mg, 1 or 5 mol%), LiBr (174 mg, 2 mmol, 1.0 equiv), 2,2'-bipyridyl (9.4 or 47 mg, 3 or 15 mol%), substrate (2 mmol, 1.0 equiv), and anhydrous DMF (5 mL). Then to the anodic chamber of the divided cell was added LiBr (174 mg, 1.0 equiv) and anhydrous DMF (5 mL). The cathodic chamber was then installed a RVC cathode and a Ag/$AgNO_3$ reference electrode, and the anodic chamber was installed a Mg anode. The two chambers were sealed with rubber septa, respectively, and to each chamber was introduced a thin Teflon tube to allow continuous nitrogen bubbling. The reaction mixture was stirred at 1000 rpm for 30 min to allow full dissolution of LiBr and exclusion of adventitious oxygen. After that, the reaction mixture was electrolyzed under a constant potential of -1.7 V (vs. Fc/Fc^+) for 18 h at room temperature. The resultant solution was directly concentrated *in vacuo*. The crude product was purified by column chromatography to furnish the desired product (eluent: hexane/ethyl acetate, 3/1).

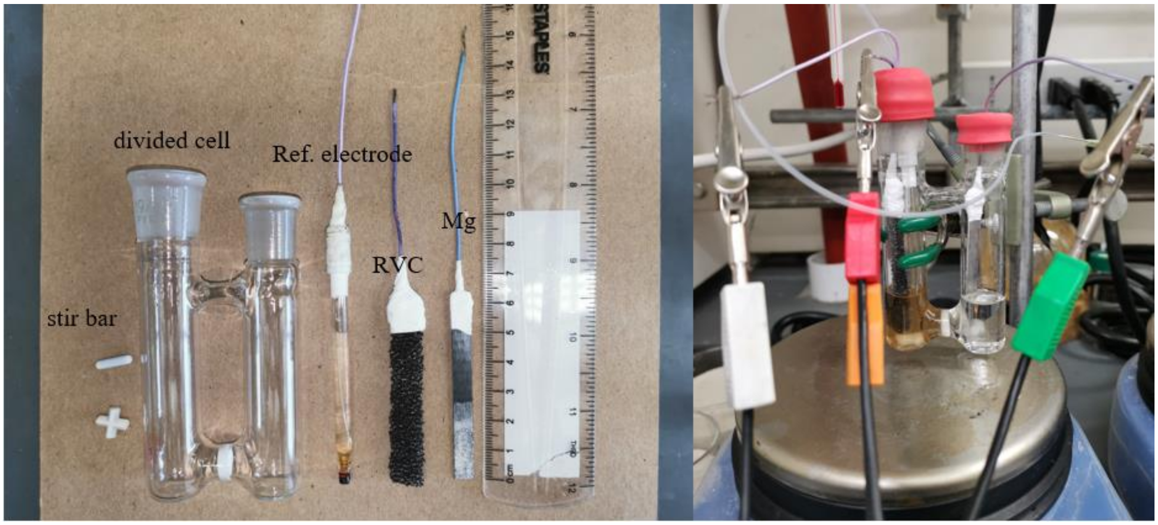

Figure S1. Graphic illustration of the divided cell before (left) and after (right) assembly.

*Electrochemical reductive homocoupling in an undivided cell (**GP 3**)*

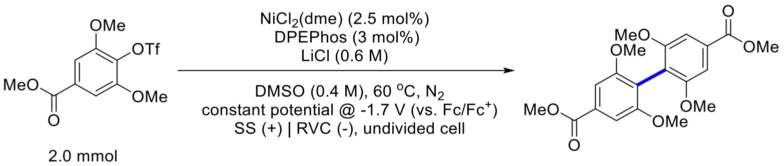

To the undivided cell (Figure S2) was added $NiCl_2$(dme) (11 mg, 2.5 mol%), DPEPhos (32 mg, 3 mol%), LiCl (125 mg, 3 mmol, 1.5 equiv), and substrate (2 mmol, 1.0 equiv). The cell was then introduced in a nitrogen-filled glove box and installed a RVC cathode, a Ag/$AgNO_3$ reference electrode, and a stainless steel anode. Anhydrous DMSO (5 mL) was injected into the cell with a 5 mL syringe. The cell was sealed with a rubber septum and removed from the glove box. A thin Teflon tube was introduced immediately into the cell to allow continuous nitrogen bubbling. The reaction mixture was stirred at 1000 rpm for 30 min to allow full dissolution of LiCl and exclusion of adventitious oxygen. After that, the reaction mixture was electrolyzed under a constant potential of -1.7 V (vs. Fc/Fc^+) for 24 h at 60 ºC. The resultant solution was directly concentrated *in vacuo*. The crude product was purified by column chromatography to furnish the desired product (eluent: hexane/ethyl acetate, 3/1).

*Electrochemical reductive cross-coupling in an undivided cell (**GP 4**)*

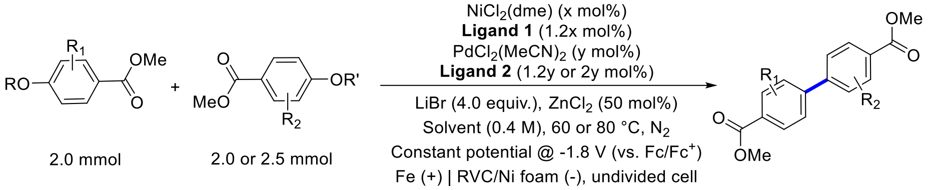

Under nitrogen atmosphere, $NiCl_2$(dme) (22 or 44 mg, 5 or 10 mol%), Ligand 1 (6 or 12 mol%), and anhydrous solvent (1 mL) was added to a 1.5-dram vial capped with a Teflon septum. Another 1.5-dram

vial was charged with $PdCl_2(MeCN)_2$ (10.4 ~ 26 mg, 2 ~ 5 mol%), Ligand 2 (4 ~ 10 mol%), and anhydrous solvent (1 mL) under nitrogen. The two vials were stirred for 30 min so that the ligands are well complexed with the metal.

To the undivided cell (Figure S2) was added the two coupling partners (2 or 2.5 mmol, 1.0 or 1.25 equiv) and the cell was introduced in a nitrogen-filled glove box and installed a Ni foam or RVC cathode, a $Ag/AgNO_3$ reference electrode, and an iron rod anode. The cell was then charged with LiBr (695 mg, 8 mmol, 4.0 equiv), $ZnCl_2$ (136 mg, 1 mmol, 0.5 equiv), the two catalyst solutions and anhydrous solvent (3 mL), sealed with rubber septum, and removed from the glove box. A thin Teflon tube was introduced immediately into the cell to allow continuous nitrogen bubbling. The reaction mixture was stirred at 1000 rpm for 30 min to allow full dissolution of LiBr and exclusion of adventitious oxygen. After that, the reaction mixture was electrolyzed under a constant potential of -1.8 V (vs. Fc/Fc^+) for 36 or 48 h at 60 or 80 ºC. The resultant solution was directly concentrated *in vacuo*. The crude product was purified by column chromatography to furnish the desired product (eluent: hexane/ethyl acetate, 3/1).

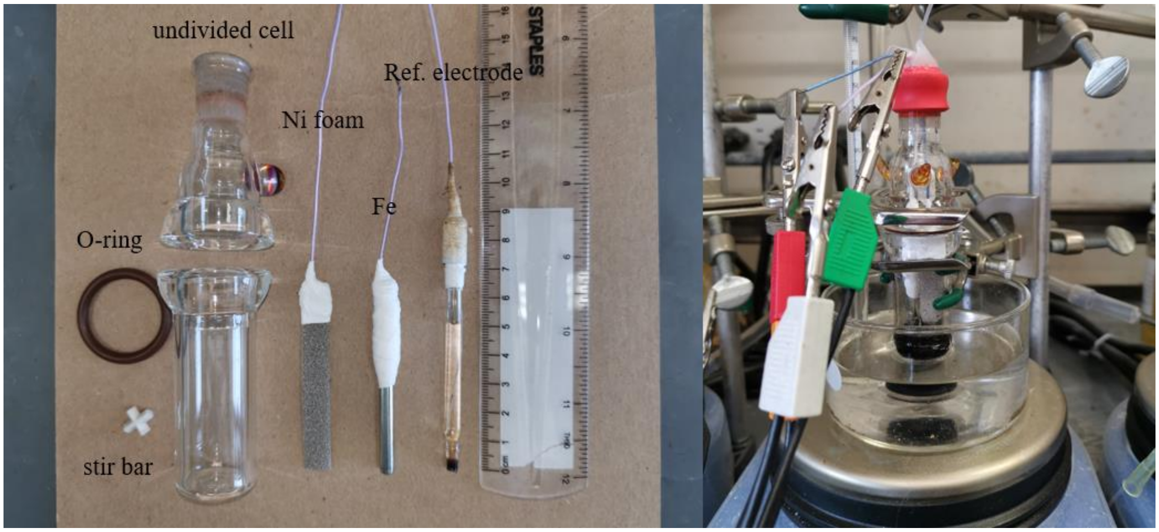

Figure S2. Graphic illustration of the undivided cell before (left) and after (right) assembly.

4. *General Procedures for Flow Electrolysis*

For the electrochemical flow reactions, a commercial Micro Flow Cell (purchased from ElectroCell) with an electrode area of 10 cm² was used, the active reactor volume is 5 mL, and a Pine WaveNow PGstat or Bipotentiostat BP-300 was used as power supply. The undivided flow cell consists of PTFE end frames, stainless steel plate (316L) as the anode, stainless steel plate and graphite plate overlay together as the cathodic electron collector. The flow cell also contains the flow frames and gaskets. RVC with approx. dimensions = 3.5 × 3.0 × 0.5 cm was used to increase the surface area of cathode and as a turbulence material for diffusion. All electrolysis reactions were performed in DMF solutions. A magnetic stir bar was used in the reservoirs and the reaction mixture was stirred (600 rpm) during flow electrolysis reactions. The reaction mixture is pumped through the system via peristaltic pump with a flow rate of 40 mL/min. The components of the electrochemical cell are shown in Figure S3.

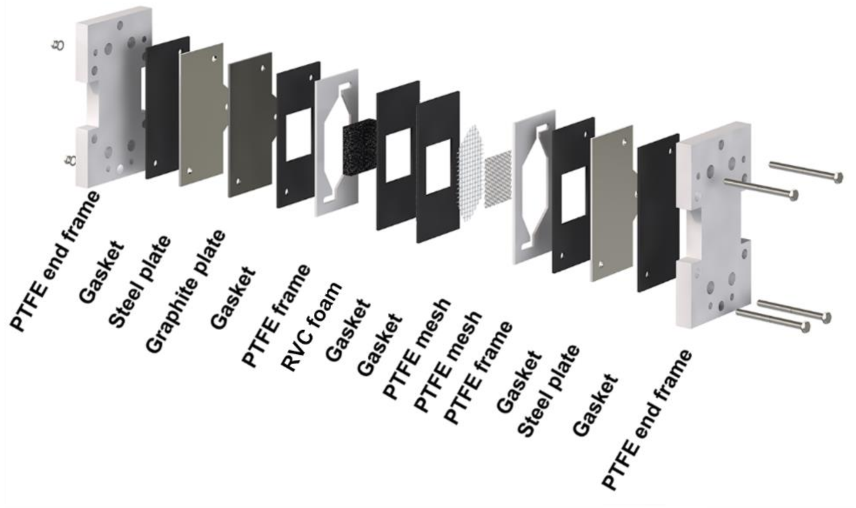

Figure S3. Graphic illustration of the components of the undivided flow cell reactor. Figure adapted with permission from Ref. 3, Copyright 2021, *Org. Process Res. Dev*.

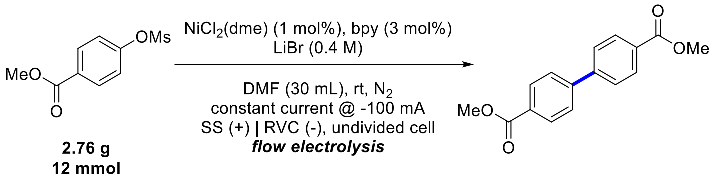

12 mmol Scale: To a 100 mL round-bottom flask was injected a solution containing $NiCl_2$(dme) (26.4 mg, 0.12 mmol, 1 mol%), LiBr (1044 mg, 12 mmol, 1.0 equiv), 2,2'-bipyridyl (56.4 mg, 0.36 mmol, 3 mol%), methyl 4-((methylsulfonyl)oxy)benzoate (2.76 g, 12 mmol, 1.0 equiv), and anhydrous DMF (30 mL). A thin Teflon tube was introduced immediately into the flask to allow continuous nitrogen bubbling. This solution was pushed via a peristaltic pump to pass through the undivided flow cell, with a flow rate of 40 mL min^{-1} and electrolyzed at a constant current of -100 mA at room temperature until full conversion of the substrate was determined by TLC. The resultant solution was directly concentrated *in vacuo* and analyzed by ^{1}H NMR using mesitylene as an internal standard to give the NMR yield (87%).

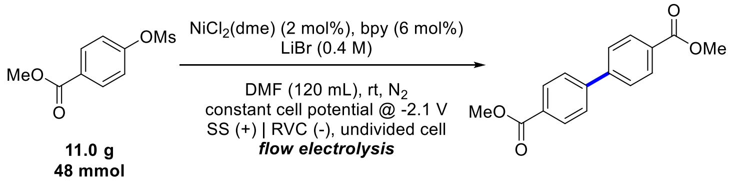

48 mmol scale: To a 250 mL three-neck round-bottom flask was injected a solution containing $NiCl_2$(dme) (106 mg, 0.48 mmol, 1 mol%), LiBr (4.17 g, 48 mmol, 1.0 equiv), 2,2'-bipyridyl (226 mg, 1.44 mmol, 3 mol%), methyl 4-((methylsulfonyl)oxy)benzoate (11.0 g, 48 mmol, 1.0 equiv), and anhydrous DMF (120 mL). A thin Teflon tube was introduced immediately into the flask to allow continuous nitrogen bubbling. This solution was pushed via a peristaltic pump to pass through the undivided flow cells, with a flow rate of 40 mL min^{-1} and electrolyzed at a constant cell potential of -2.1 V at room temperature. Two flow cells were connected in parallel via copper wire to increase the electrode surface area in total. An additional amount of catalyst (1 mol%) (106 mg $NiCl_2$(dme), 226 mg 2,2'-bipyridyl dissolved in 5 mL DMF) was injected into the reaction mixture via a syringe after 24 h of electrolysis without stopping the reaction. Electrolysis was conducted until full conversion of the substrate was determined by TLC. The resultant

solution was directly concentrated *in vacuo* and analyzed by ^{1}H NMR using mesitylene as an internal standard to give the NMR yield (80%).

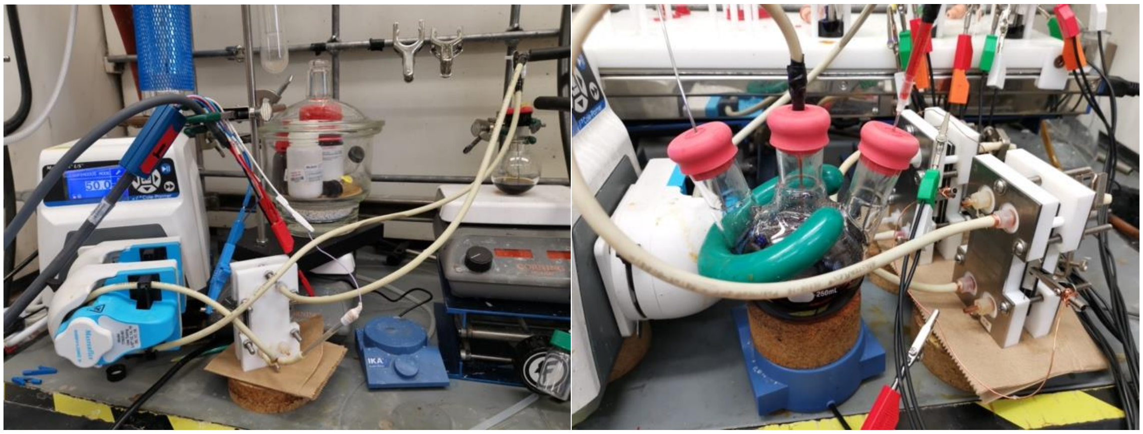

Figure S4. Graphic illustration of the undivided flow cell for 12 mmol (left) and 48 mmol (right) scale-up.

5. *Optimization of Reaction Conditions*

Optimization of Ni-catalyzed reductive homocoupling using Zn reductant

To a 1-dram vial fitted with cross-shaped stir bar was added $NiCl_2$(dme), the appropriate ligand, additive, substrate (0.4 mmol, 1.0 equiv), and Zn dust (52 mg, 0.8 mmol, 2.0 equiv, unless otherwise noted). This vial was transferred into a nitrogen-filled glove box, then additive and solvent (1 mL, unless otherwise noted) were added. The vial was capped with a screw cap fitted with a PTFE-faced silicone septum, then removed from the glovebox and heated in a sand bath to the desired temperature with stirring (1000 rpm) for 15 – 27 h. The reaction mixture was then cooled to room temperature and diluted with DMF (3 mL). Mesitylene (1.0 equiv) was added to the crude material. Then a 100 µL aliquot of the solution was filtered through a 2-cm silica gel plug in a Pasteur pipette into a vial. 500 µL $CDCl_3$ was also added to the pipette and eluted into the same vial. The resultant sample was analyzed by ^{1}H NMR spectroscopy and the yields were determined using mesitylene as the internal standard.

Table S1. Optimization of thermochemical Ni-catalyzed reductive homocoupling of G–OMs[a]

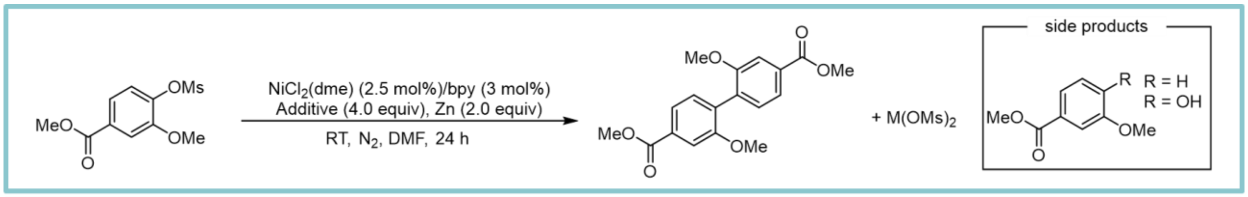

Entry	Additive	Concentration (M)	MB	Conversion	C-H bp	C-OH bp	Product
1[b]	LiBr	0.4	93%	100%	90%	0%	3%
2[c]	Et_4NI	1.0	94%	63%	3%	0%	45%
3	NaBr	0.25	95%	100%	86%	3%	6%
4	NaBr	0.5	93%	100%	79%	3%	11%
5	NaBr	0.5	92%	100%	76%	4%	12%
6	NaBr	0.25	98%	48%	27%	16%	3%
7[d]	LiBr	0.25	94%	33%	7%	18%	2%
8	KBr	0.25	97%	100%	38%	0%	59%
9[e]	LiBr	0.25	95%	100%	59%	20%	16%
10[f]	LiBr	0.25	99%	92%	40%	15%	36%

[a] Yields were determined by ^{1}H NMR spectroscopy using mesitylene as the internal standard. [b] 5 mol% $NiCl_2$(dme), 15 mol% bpy, 0.4 M LiBr, 0.4 M **G–OMs**. [c] Following the Percec condition[4]: 10 mol% $Ni(PPh_3)_2Cl_2$, 1.5 equiv Et_4NI, 1.7 equiv Zn dust, THF, 1.0 M **G–OMs**, 67 ºC. [d] < 5% yield was obtained with 8 more bpy derivatives (shown is with 2,2'-bipyridine). [e] With 1,10-Phen as ligand. [f] With 2,9-dimethyl-1,10-Phen as ligand.

Table S2. Conditions screening focused on nitrogen-based ligands for S/S homocoupling[a]

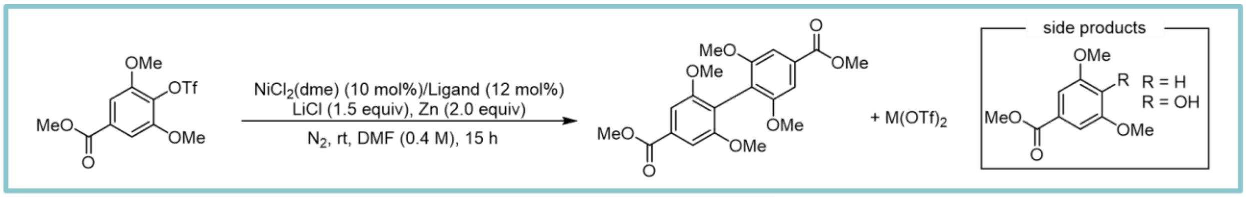

Ligand	Substrate	Conversion	MB	C-H bp	C-OH bp	Product
1	25%	75%	91%	26%	32%	8%
2	14%	86%	90%	50%	25%	1%
3	26%	74%	95%	29%	34%	6%
4	23%	77%	94%	31%	39%	1%
5	33%	67%	93%	27%	23%	10%
6	47%	53%	102%	25%	23%	7%
7	36%	64%	94%	27%	26%	5%
8	18%	82%	93%	30%	35%	10%
9	15%	85%	90%	35%	35%	5%
10	55%	45%	103%	22%	25%	1%
11	1%	99%	87%	79%	6%	1%
12	9%	91%	92%	64%	12%	7%

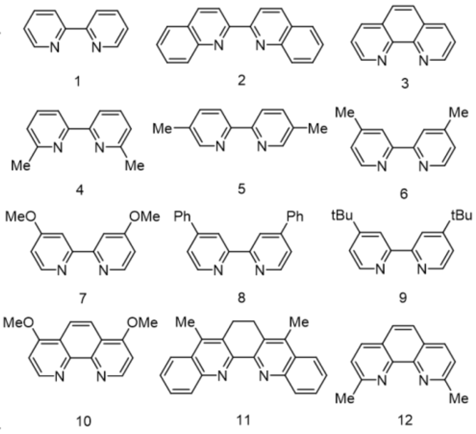

[a] Yields were determined by ^{1}H NMR spectroscopy using mesitylene as the internal standard.

Table S3. Conditions screening focused on nitrogen-based ligands for S/S homocoupling[a]

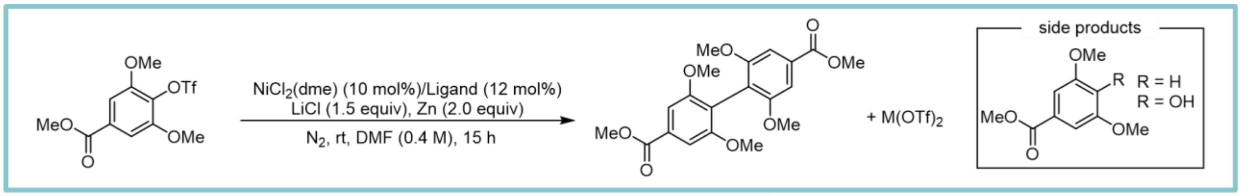

Ligand	Substrate	Conversion	MB	C-H bp	C-OH bp	Product
1	15%	85%	82%	29%	35%	3%
2	46%	54%	97%	24%	24%	3%
3	30%	70%	101%	44%	27%	0%
4	61%	39%	98%	14%	22%	1%
5	0%	100%	95%	88%	4%	3%
6	60%	40%	99%	18%	21%	0%
7	0%	100%	78%	38%	40%	0%
8	46%	54%	97%	19%	32%	0%
9	43%	57%	86%	16%	27%	0%
10	7%	93%	70%	36%	27%	0%
11	9%	91%	86%	45%	32%	0%
12	42%	58%	90%	32%	16%	0%

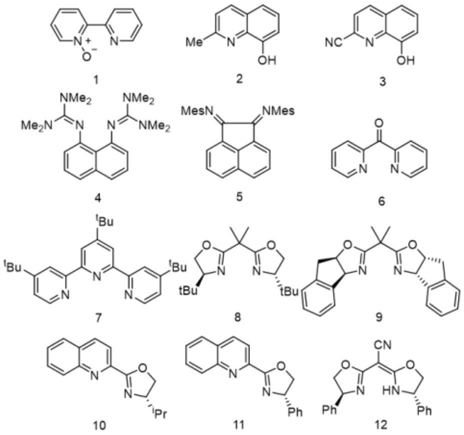

[a] Yields were determined by ^1H NMR spectroscopy using mesitylene as the internal standard.

Table S4. Conditions screening focused on phosphine-based ligands for S/S homocoupling[a]

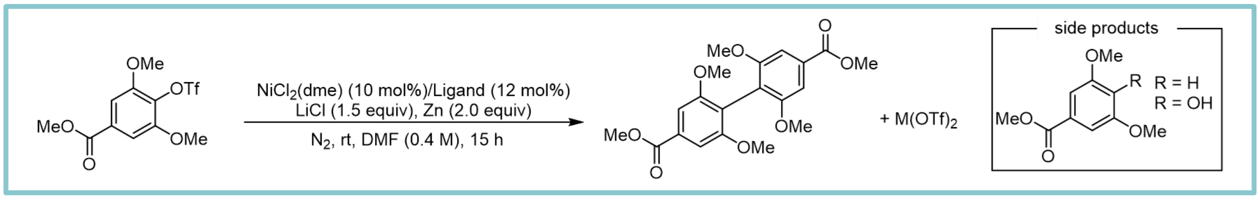

Ligand	Substrate	Conversion	MB	C-H bp	C-OH bp	Product
1	37%	63%	89%	22%	29%	1%
2	49%	51%	86%	17%	20%	0%
3	42%	58%	91%	20%	29%	0%
4	47%	53%	83%	15%	21%	0%
5	80%	20%	94%	6%	8%	0%
6	44%	56%	93%	20%	29%	0%
7	42%	58%	90%	19%	29%	0%
8	35%	65%	70%	13%	22%	0%
9	31%	69%	80%	21%	28%	0%
10	77%	23%	93%	5%	9%	2%
11	36%	64%	84%	21%	27%	0%
12	57%	43%	97%	14%	26%	0%
13	52%	48%	96%	16%	28%	0%
14	44%	56%	77%	12%	21%	0%
15	43%	57%	82%	17%	22%	0%
16	38%	62%	89%	33%	18%	0%
17	36%	64%	87%	33%	18%	0%
18	32%	68%	94%	48%	9%	5%
19	18%	82%	99%	54%	11%	16%
20	55%	45%	94%	13%	26%	0%

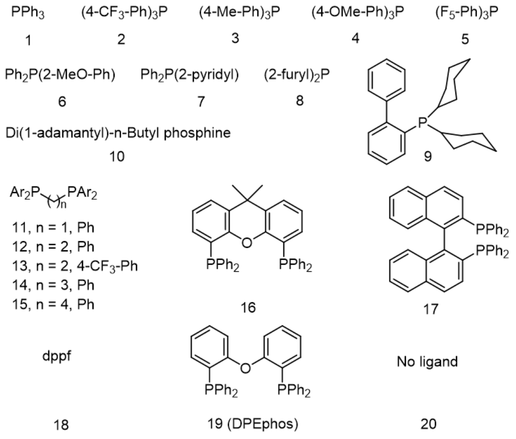

[a] Yields were determined by ^1H NMR spectroscopy using mesitylene as the internal standard.

Table S5. Conditions screening focused on ligand loading and additives for S/S homocoupling[a]

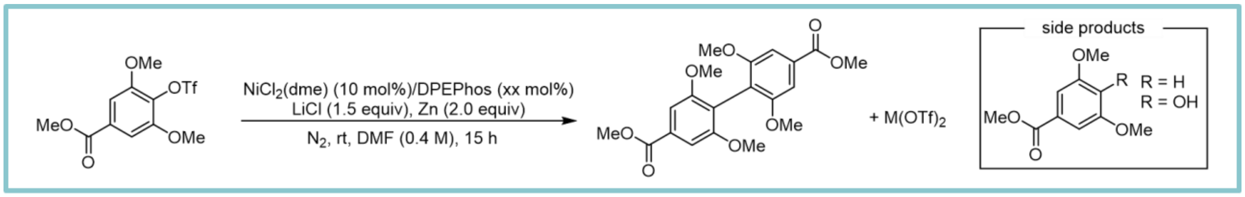

Entry	Ligand loading (xx mol%)	MB	Conversion	C–H bp	C–OH bp	Yield
1	8 mol%	82%	82%	44%	3%	17%
2	12 mol%	83%	82%	46%	4%	15%
3	15 mol%	92%	75%	46%	8%	13%
4	20 mol%	84%	59%	36%	12%	6%
5[b]	12 mol%	93%	69%	55%	3%	4%
6[c]	12 mol%	82%	77%	54%	2%	3%
7[d]	12 mol%	93%	61%	38%	12%	4%
8[e]	12 mol%	99%	74%	72%	0%	1%

[a] Yields were determined by ^{1}H NMR spectroscopy using mesitylene as the internal standard. [b] LiBr instead of LiCl. [c] $^{n}Bu_4NBr$ instead of LiCl. [d] $^{n}Bu_4NCl$ instead of LiCl. [e] $^{n}Bu_4NI$ instead of LiCl.

Table S6. Conditions screening focused on temperature and solvents for S/S homocoupling[a]

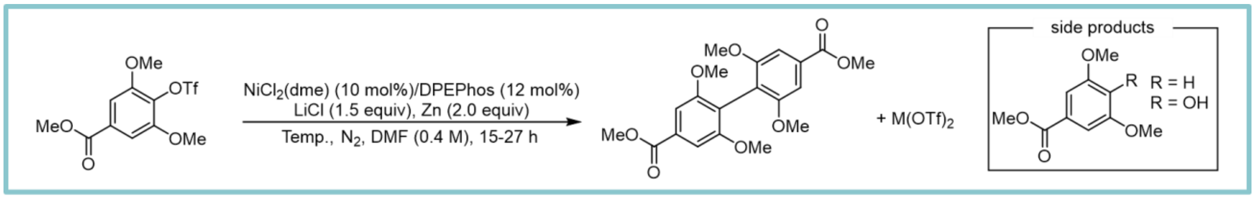

Entry	Temperature	Solvent	MB	Conversion	C–H bp	C–OH bp	Yield
1	rt	DMF	83%	82%	46%	4%	15%
2	~ 40 °C	DMF	85%	>99%	58%	<3%	24%
3	~ 50 °C	DMF	90%	>99%	49%	5%	36%
4	~ 60 °C	DMF	85%	98%	37%	8%	**38%**
5	~ 70 °C	DMF	93%	91%	42%	10%	32%
6	~ 80 °C	DMF	73%	>99%	35%	26%	12%
7	rt	CH_3CN	76%	>99%	72%	0%	4%
8	~ 60 °C	CH_3CN	87%	>99%	73%	0%	10%
9	rt	THF	90%	58%	45%	3%	0%
10	~ 60 °C	THF	76%	98%	66%	1%	7%
11	rt	DMA	88%	75%	37%	7%	19%
12	~ 60 °C	DMA	82%	89%	32%	10%	29%

[a] Yields were determined by ^{1}H NMR spectroscopy using mesitylene as the internal standard.

Table S7. Conditions screening focused on additive loadings for S/S homocoupling[a]

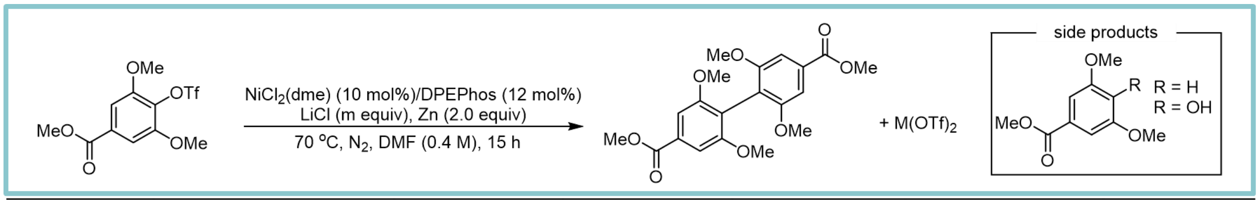

Entry	m	MB	Conv.	C–H bp	C–OH bp	Yield
1	1.0	94%	99%	75%	1%	17%
2	1.5	93%	91%	42%	10%	32%
3	2.5	103%	93%	49%	22%	25%
4	4.0	81%	>99%	36%	36%	9%
5	6.0	79%	>99%	31%	40%	8%

[a] Yields were determined by ^{1}H NMR spectroscopy using mesitylene as the internal standard.

Table S8. Conditions screening focused on molecular sieves and solvents for S/S homocoupling[a]

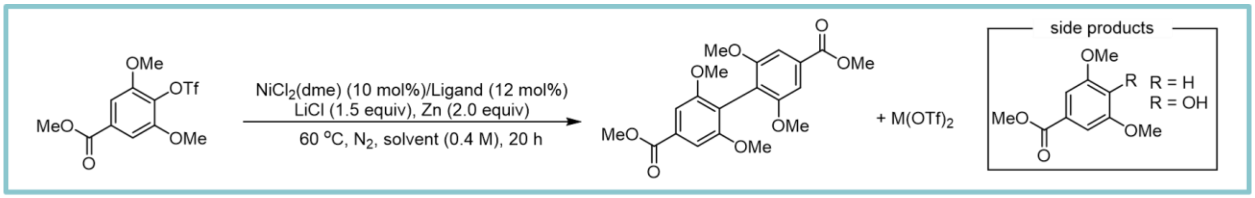

Entry	Ligand	Mol. sieves (100 mg)	Solvent	Conv.	C–H bp	C–OH bp	Yield
1	DPEPhos	No	DMF	77%	29%	23%	27%
2	DPEPhos	No	DMA	74%	29%	7%	33%
3	DPEPhos	Yes	DMF	85%	30%	15%	36%
4	DPEPhos	Yes	DMA	83%	37%	7%	37%
5	DPEPhos	Yes	DMSO	100%	32%	5%	55%
6	DPEPhos	Yes	NMP	58%	20%	15%	17%
7	DPEPhos	Yes	DMPU	100%	32%	61%	0%
8	DCEPhos	Yes	DMF	39%	15%	19%	0%
9	DCEPhos	Yes	DMA	34%	13%	17%	0%
10	DCEPhos	Yes	DMSO	35%	14%	30%	0%
11	DCEPhos	Yes	NMP	67%	1%	35%	0%
12	DCEPhos	Yes	DMPU	47%	41%	8%	2%

[a] Yields were determined by ^{1}H NMR spectroscopy using mesitylene as the internal standard.

Optimization of electrochemical Ni-catalyzed reductive S/S homocoupling

To a divided or undivided cell (Figure S1, S2) was added $NiCl_2$(dme), DPEPhos, LiCl (125 mg, 3 mmol, 1.5 equiv), and methyl 3,5-dimethoxy-4-(((trifluoromethyl)sulfonyl)oxy)benzoate (688 mg, 2 mmol, 1.0 equiv). The cell was then introduced in a nitrogen-filled glove box and installed a RVC cathode, a Ag/$AgNO_3$ reference electrode, and a sacrificial anode. Anhydrous DMSO (5 mL) was injected into the cell with a 5 mL syringe. The cell was sealed with a rubber septum and removed from the glove box. A thin Teflon tube was introduced immediately into the cell to allow continuous nitrogen bubbling. The reaction

mixture was stirred at 1000 rpm for 30 min to allow full dissolution of LiCl and exclusion of adventitious oxygen. After that, the reaction mixture was electrolyzed under a constant potential of -1.7 V (vs. Fc/Fc^+) for 24 h at 60 ºC. The reaction mixture was then cooled to room temperature and diluted with DMF (25 mL). Mesitylene (278 µL, 2 mmol, 1.0 equiv) was added to the crude material. Then a 150 µL aliquot of the solution was filtered through a 2-cm silica gel plug in a Pasteur pipette into a vial. 500 µL $CDCl_3$ was also added to the pipette and eluted into the same vial. The resultant sample was analyzed by ^1H NMR spectroscopy and the yields were determined using mesitylene as the internal standard. The results are summarized in Table S9. We postulated that the metal salts from anode oxidation could serve as an overcharge protector in an undivided cell to prevent overreduction of redox-active species in the solution,[5] thus explaining the poor results in entries 6 and 7.

Table S9. Optimization of electrochemical Ni catalyzed reductive S/S homocoupling[a]

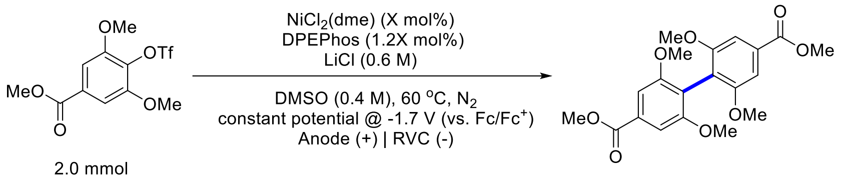

Entry	Cell type	Anode	X	Mass balance (%)	Ar–H (%)	ArO–H (%)	Yield (%)
1	undivided	stainless steel	10	95	18	2	73
2[b]	undivided	stainless steel	5	103	16	2	75
3	undivided	stainless steel	5	99	22	0	72
4	undivided	Fe	5	95	20	0	65
5	undivided	stainless steel	2.5	100	12	0	**78**
6[c,d]	undivided	stainless steel	\	97	34	0	0
7[d]	divided	Fe	5	88	11	1	12
8[d]	divided	Mg	5	100	0	0	0

[a] Yields were determined by ^1H NMR spectroscopy using mesitylene as the internal standard. [b] Added 500 mg 4 Å molecular sieves. [c] No Ni catalyst. [d] The rest of the mass corresponds to unreacted starting material.

Ni-only catalyzed reductive cross-coupling

To a 1-dram vial fitted with cross-shaped stir bar was added $NiCl_2$(dme) (2.2 mg, 5 mol%), the appropriate ligand, two coupling partners (0.2 mmol, 1.0 equiv), and Zn dust (26 mg, 0.4 mmol, 2.0 equiv). This vial was transferred into a nitrogen-filled glove box, then LiBr (87 mg, 0.2 mmol, 1.0 equiv) and DMA (1 mL) were added. The vial was capped with a screw cap fitted with a PTFE-faced silicone septum, then removed from the glovebox and heated in a sand bath to 60 ºC with stirring (1000 rpm) for 17 h. The reaction mixture was then cooled to room temperature and diluted with DMA (3 mL). Mesitylene (27.8 µL, 0.2 mmol, 1.0 equiv) was added to the crude material. Then a 100 µL aliquot of the solution was filtered through a 2-cm silica gel plug in a Pasteur pipette into a vial. 500 µL $CDCl_3$ was also added to the pipette and eluted into the same vial. The resultant sample was analyzed by ^1H NMR spectroscopy and the yields were determined using mesitylene as the internal standard. The results are summarized in Table S10.

Table S10. Ni-only catalyzed reductive cross-coupling of LDMs.[a]

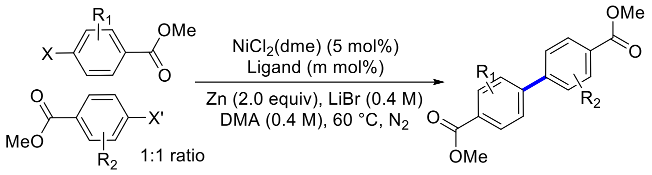

Entry	Ligand	m	Ar–X	Ar–X'	Yield (%)	Hetero:Homo
1	bpy	15	H–OMs	G–OTs	7	2.0
2[b]	bpy	30	H–OTs	S–OTf	48	1.1
3	bpy	15	G–OTs	S–OTf	0	N.A.
4	DPEPhos	6	H–OMs	G–OTs	14	1.3
5	DPEPhos	6	H–OTs	S–OTf	27	1.9
6	DPEPhos	6	G–OTs	S–OTf	0	N.A.

[a] Yields were determined by ^{1}H NMR spectroscopy using mesitylene as the internal standard.
[b] 10 mol% $NiCl_2$(dme).

Optimization of electrochemical Ni/Pd-catalyzed reductive cross-coupling

Under nitrogen atmosphere, $NiCl_2$(dme) (22 mg, 5 mol%), 4,4'-dPhbpy (37 mg, 6 mol%), and DMA (1 mL) was added to a 1.5-dram vial capped with a Teflon septum. Another 1.5-dram vial was charged with $PdCl_2(MeCN)_2$ (26 mg, 5 mol%), dppb (51 mg, 6 mol%), and DMA (1 mL) under nitrogen. The two vials were stirred for 30 min so that the ligands are well complexed with the metal.

To the undivided cell (Figure S2) was added the two coupling partners (2 mmol, 1.0 equiv) and the cell was introduced in a nitrogen-filled glove box and installed a Ni foam cathode, a $Ag/AgNO_3$ reference electrode, and an iron rod anode. The cell was then charged with LiBr (695 mg, 8 mmol, 4.0 equiv), $ZnCl_2$ (136 mg, 1 mmol, 0.5 equiv), the two catalyst solutions and DMA (3 mL), sealed with rubber septum, and removed from the glove box. A thin Teflon tube was introduced immediately into the cell to allow continuous nitrogen bubbling. The reaction mixture was stirred at 1000 rpm for 30 min to allow full dissolution of LiBr and exclusion of adventitious oxygen. After that, the reaction mixture was electrolyzed under a constant potential of -1.8 V (vs. Fc/Fc^+) for 36 h at 60 ºC. The reaction mixture was then cooled to room temperature and diluted with DMA (30 mL). 1,3,5-trimethoxybenzene (168 mg, 1 mmol, 0.5 equiv) was added to the crude material. Then a 150 µL aliquot of the solution was filtered through a 2-cm silica gel plug in a Pasteur pipette into a vial. 500 µL $CDCl_3$ was also added to the pipette and eluted into the same vial. The resultant sample was analyzed by ^{1}H NMR spectroscopy and the yields were determined using 1,3,5-trimethoxybenzene as the internal standard. The results are summarized in Figure S5.

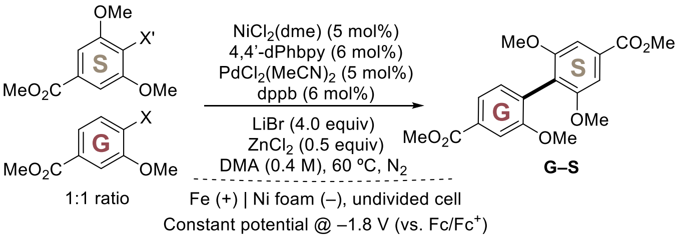

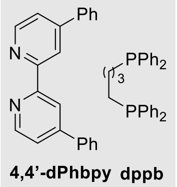

Entry	Ar–X	Ar–X'	Yield (%)	Hetero:homo
1	G–OMs	S–OTs	3	1:3.7
2	G–OTs	S–OTs	15	1:4
3	G–OTf	S–OTs	4	1:10
4	G–OMs	S–OTf	0	N.A.
5	G–OTs	S–OTf	14	1:1.6
6	G–OTf	S–OTf	8	1:4.2

Figure S5. Electrochemical Ni/Pd-catalyzed G/S cross-coupling. Yields were determined by ^{1}H NMR spectroscopy using 1,3,5-trimethoxybenzene as the internal standard.

Optimization of Ni/Pd-catalyzed reductive cross-coupling (HTE Optimization)

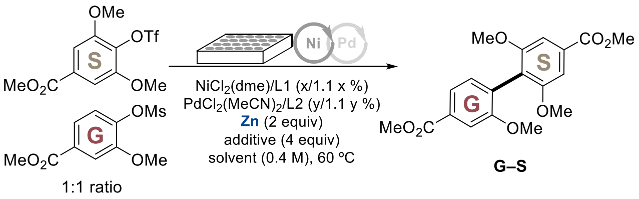

To a 2-dram vial fitted with cross-shaped stir bar was added $NiCl_2$(dme) and appropriate nitrogen-based ligand. This vial was then transferred into a nitrogen-filled glove box and solvent was added. In a separate 2-dram vial fitted with a cross-shaped stir bar was added $PdCl_2(MeCN)_2$ and appropriate phosphine ligand. This vial was then transferred into a nitrogen-filled glove box and solvent was added. These stock solutions were stirred for 1 h. To a 96-well optimization block (Analytical Sales and Services) with 1-mL glass vial inserts (Analytical Sales and Services) fitted with stainless-steel stir bars (V&P scientific) in a nitrogen-filled glove box, was dispensed appropriate quantities of the stock solutions of the catalysts (concentrations of stock solutions were adjusted so that around 10 μL of each stock solution was dispensed). The blocks were then aged for 15 minutes. To a 2-dram vial fitted with a cross-shaped stir bar was added both aryl sulfonate coupling partners (0.40 M, 1.0 equiv), LiBr (1.60 M, 4 equiv), Zn dust (0.80 M, 2.0 equiv), and solvent. This mixture was then stirred vigorously for 5 minutes. To the aged 96-well optimization block, 50 μl of a suspension containing the two aryl sulfonates (20 mmol, 0.40 M), LiBr (80 mmol, 1.6 M), and Zn dust (40 mmol, 0.80 M) in solvent was dispensed to each vial from the rapidly stirred 2-dram vial containing substrate, LiBr, and Zn. The plate was then sealed with a screwdriver and placed in a zip lock bag inside the glove box. The plate was then removed from the glove box and agitated on a tumble stirrer (V&P Scientific) at 60 ºC for 20 hours. The block was then diluted with a solution of 1,3,5-trimethoxybenzene in 3:1 MeCN/DMSO (10 mmol, 0.067 M, 150 mL) and sampled (5 μL) into an HPLC collection block (Analytical Sales and Services) pre-filled with 3:1 MeCN/DMSO (200 μL). The HPLC collection block was then analyzed utilizing UPLC-MS (Waters-Acquity) analysis and yields were determined with respect to 1,3,5-trimethoxybenzene utilizing calibration curves. Data was then visualized on Tableu®. Changes were made to this procedure to minimize the number of operations for each variable that was evaluated. It is worth noting that CyJohnPhos gave a higher selectivity in Zn-mediated chemical screening but was ineffective under electrochemical conditions, furnishing the desired product in only 14%

yield. UPLC-MS analysis of the reaction mixture in this case revealed the presence of significant quantities of CyJohnPhos phosphine oxide suggesting the possibility of an electrochemical catalyst decomposition pathway not observed with Zn as the terminal reductant. Thus, SPhos was used in the co-catalyst system presented in Figure 3B of the manuscript.

Separate stock solutions for each ligand were prepared before addition to the 96-well plate to ensure pre-complexation. Ligand structures are shown below:

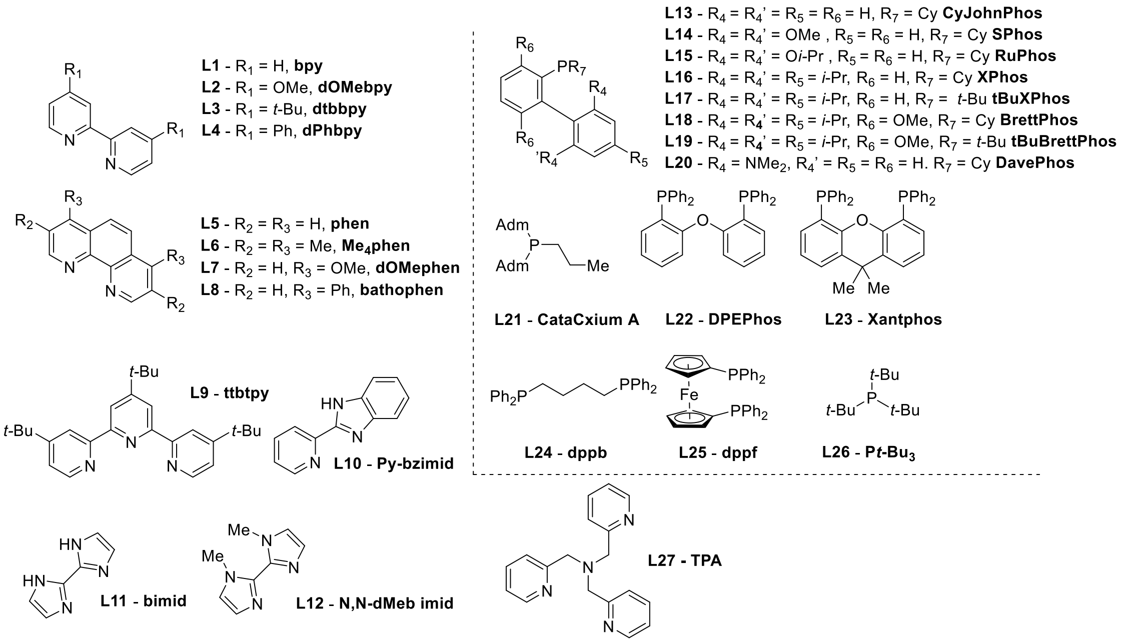

Table S11. Ligand, additive, and solvent screening for G/S cross-coupling[a]

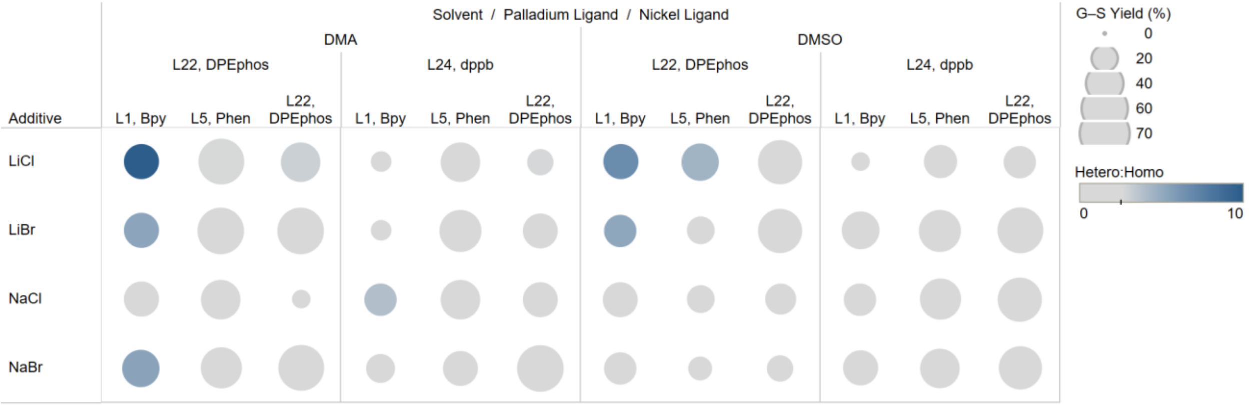

[a] Yields were determined by UPLC using 1,3,5-trimethoxybenzene as the internal standard.

Here, the Hetero:Homo ratio is defined as $\frac{cross-coupled\ product\ yield}{sum\ of\ the\ two\ homocoupled\ byproduct\ yields}$ and applies to Tables S11 to S14.

Table S12. Ligand screening for G/S cross-coupling[a]

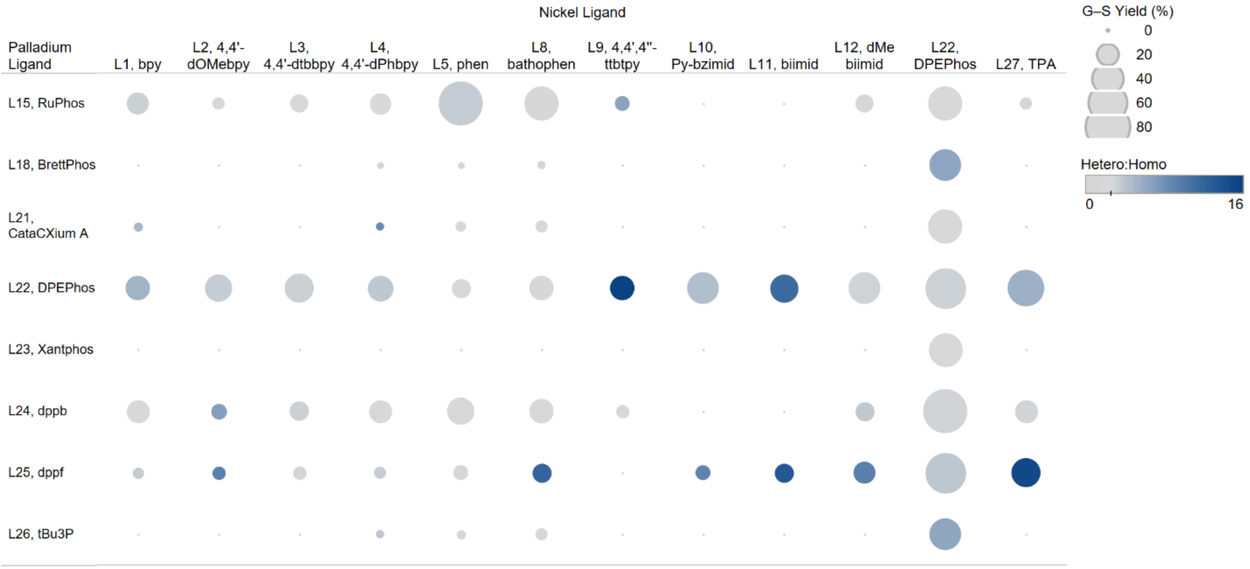

[a] Yields were determined by UPLC using 1,3,5-trimethoxybenzene as the internal standard, DMSO solvent.

Table S13. Ligand and solvent screening for G/S cross-coupling[a]

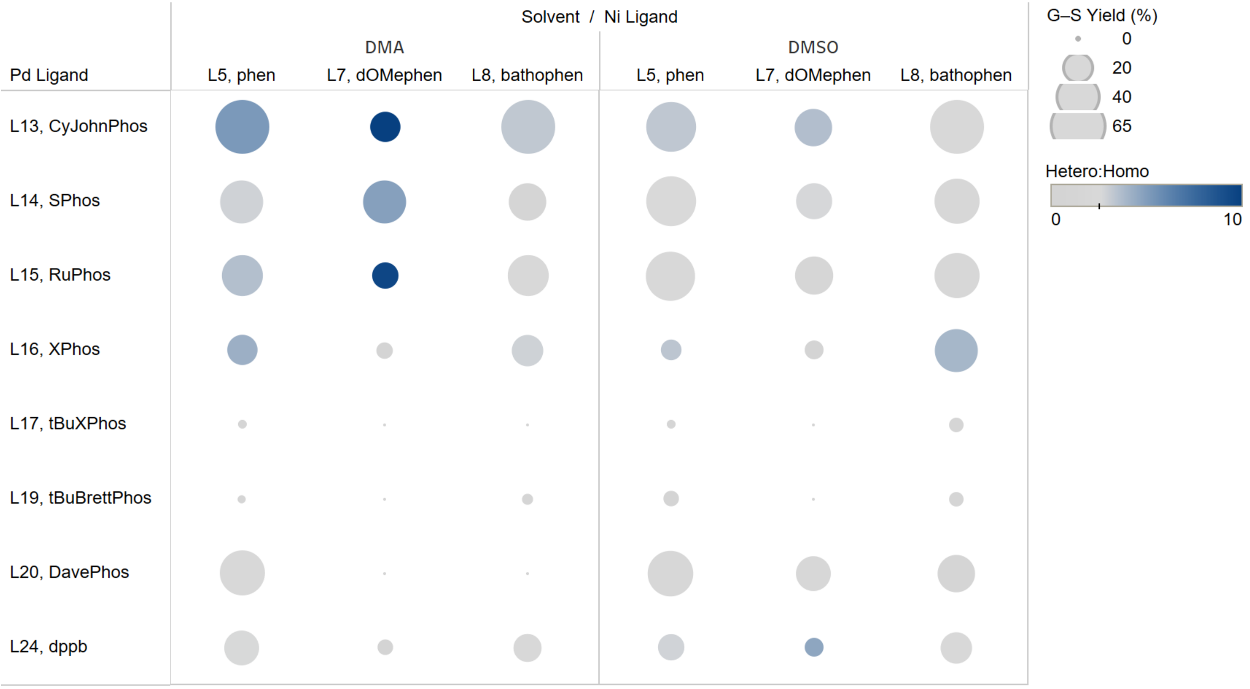

[a] Yields were determined by UPLC using 1,3,5-trimethoxybenzene as the internal standard.

Table S14. Catalyst loading screening for G/S cross-coupling[a]

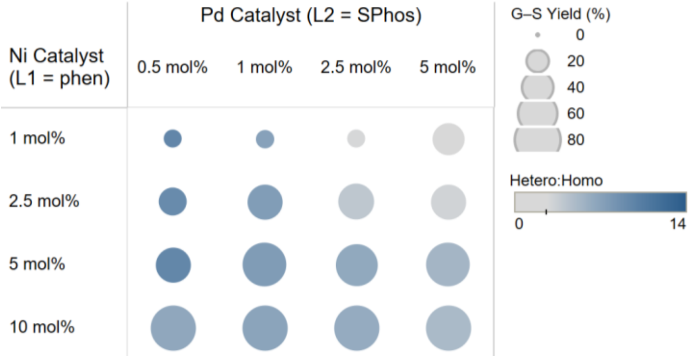

[a] Yields were determined by UPLC using 1,3,5-trimethoxybenzene as the internal standard. This table is also presented in Figure 3A.

6. *CV Studies*

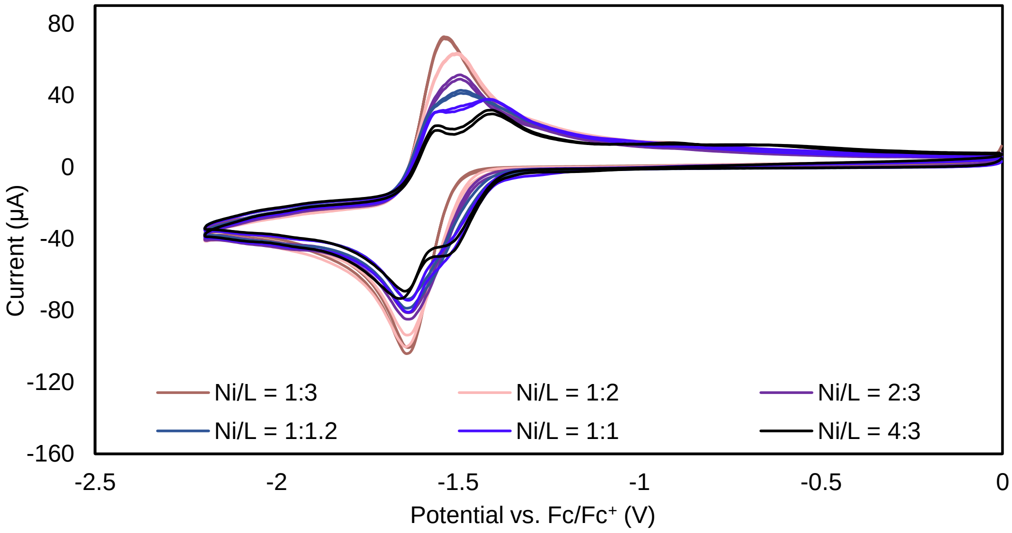

Figure S6. CVs of $NiCl_2$(dme) (denoted as Ni, 10 mM) in DMF with different loadings of bpy ligand (denoted as L), with NaBr (0.4 M) as supporting electrolyte, under N_2 protection, scan rate = 100 mV/s. Each experiment was scanned twice.

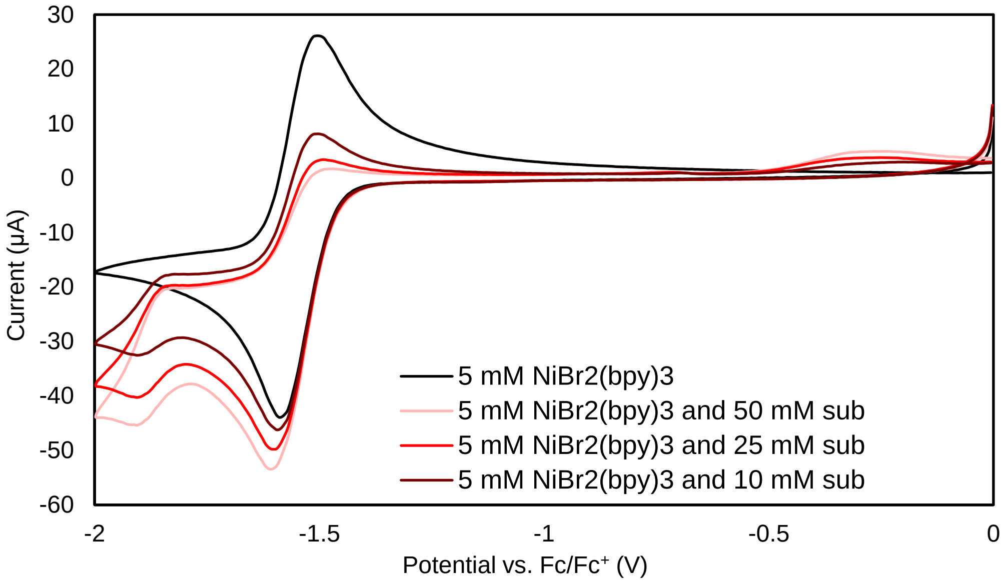

Figure S7. CVs of $NiBr_2(bpy)_3$ (5 mM) in DMF with different loadings of substrate **G–OMs** (denoted as sub), with LiBr (0.4 M) as supporting electrolyte, under N_2 protection, scan rate = 20 mV/s. $NiBr_2(bpy)_3$ was synthesized according to literature reports for the ease of CV studies.[6]

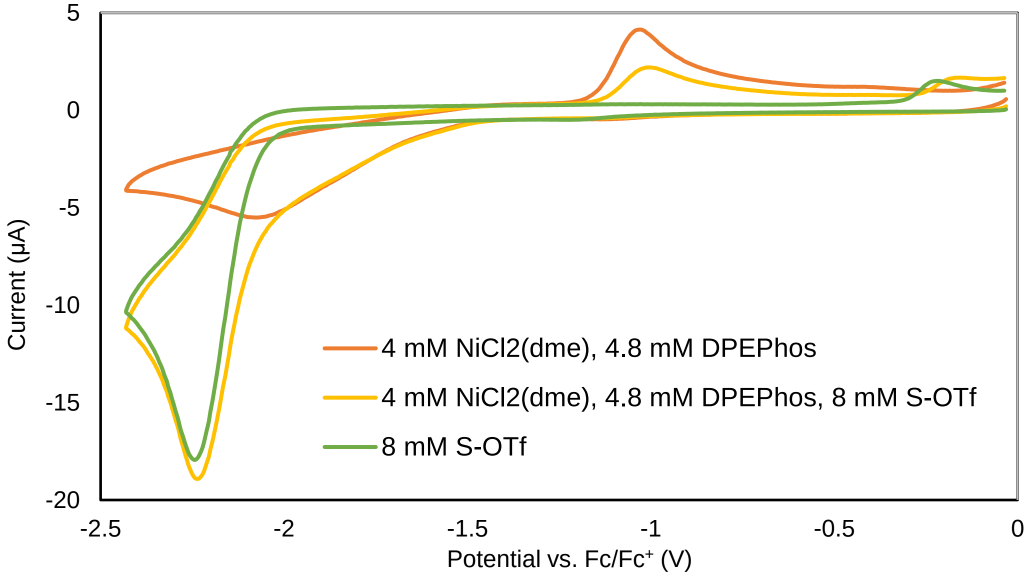

Figure S8. CVs of $NiCl_2(dme)$ (4 mM) and DPEPhos (4.8 mM) in DMSO with or without substrate **S–OTf** (8 mM), with LiBr (0.6 M) as supporting electrolyte, under N_2 protection, scan rate = 20 mV/s.

7. *Plasticizers*

Synthesis and thermal properties of lignin-derived biphenyl plasticizers

Materials: 2-ethylhexanol, titanium butoxide, ethyl acetate, hexanes were purchased from Sigma Aldrich and used as received. Polyvinyl chloride (PVC) - unplasticized, 250 microns was purchased from Goodfellow and used as received.

Instruments: The chemical structures of the lignin biphenyl plasticizers were confirmed by ^{1}H and ^{13}C NMR on a Bruker Advance III HD 400 MHz NMR spectrometer using $CDCl_3$. The typical relaxation time (T1) used was 10 s and an average number of transient scans was 16. The ^{13}C NMR spectrum was also acquired in $CDCl_3$ with an average number of transient scans of 512. Thermal properties of plasticized PVC films and plasticizers were studied using a TA Instruments Q-500 thermal gravimetric analyzer (TGA) at a heating rate of 10 °C/min with a nitrogen flow of 60 mL/min up to 800 °C and a TA instruments Q-5000 digital scanning calorimeter (DSC) was utilized at with a heating rate of 10 °C/min and a nitrogen flow rate of 60 mL/min.

*General procedure for synthesizing lignin-derived biphenyl plasticizers (**GP 5**):* The following procedure was adapted from previous literature reports synthesizing plasticizers.[7] To a 100 mL round-bottom flask was added the lignin-derived BPDA methyl esters (**H–H** through **S–S**; 500 mg, 1.0 equiv), 2-ethyl hexanol (10 equiv) and titanium butoxide (1.5 wt%) were stirred and heated to 150 °C for 2 h. The crude reaction was monitored by TLC for full conversion of the starting material. The crude product was purified by column chromatography using a gradient of hexanes to 10/1 hexane/ethyl acetate to furnish purified plasticizer as a transparent to slightly yellow tinted, viscous oil.

Preparation of plasticized PVC films: The following procedure was adapted from previous literature reports synthesizing polyesters.[8] Unplasticized polyvinyl chloride (UPVC) (100 mg) was solubilized in 1 mL THF and 10 weight percent of each plasticizer was added and stirred for 30 min. The resulting solubilized polymer solution was cast in a mold and slowly evaporated for 48 h. The films were then placed in an ambient vacuum oven under reduced pressure for 24 h to remove residual THF and the thin films were obtained for analysis.

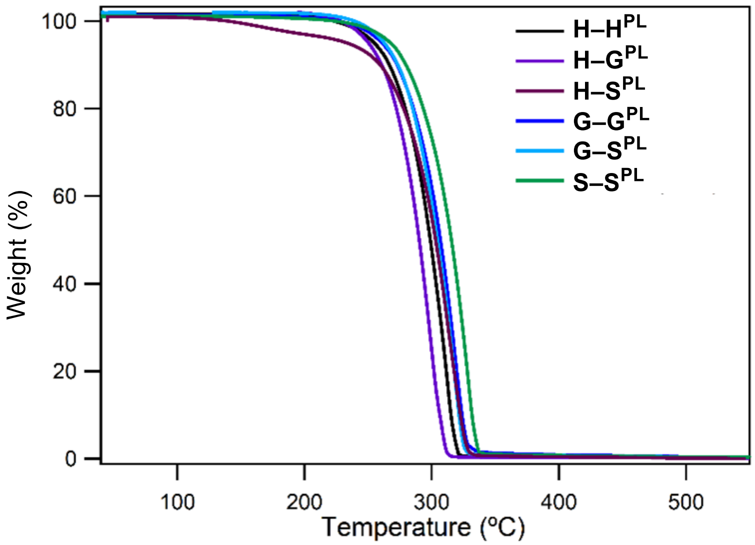

Figure S9. Thermogravimetric analysis of DEH-BPDA derivatives (**H–H^{PL}** through **S–S^{PL}**) at a heating rate of 10 °C/min under nitrogen.

Table S15. Thermogravimetric analysis of DEH-BPDA derivatives (H–H^{PL} through S–S^{PL}).

Plasticizer	T_{d10} (°C)[a]	T_{d50} (°C)[b]
**H–H^{PL}**	267	299
**H–G^{PL}**	260	290
**H–S^{PL}**	259	303
**G–G^{PL}**	272	307
**G–S^{PL}**	273	305
**S–S^{PL}**	280	316

[a] **T_{d10}** corresponds to the temperature at which 10% mass loss is observed.
[b] **T_{d50}** corresponds to the temperature at which 50% mass loss is observed.

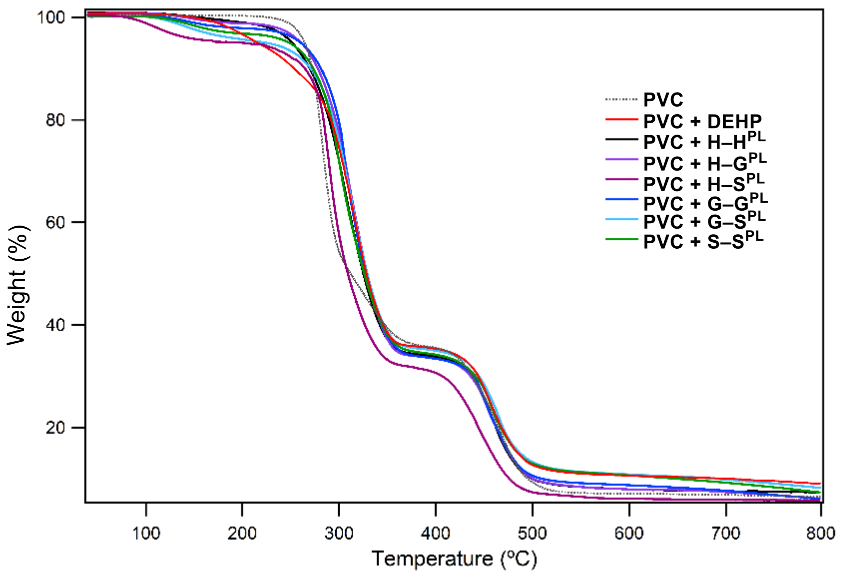

Figure S10. Thermogravimetric analysis of PVC with 10 wt% DEH-BPDA derivatives (**H–H^{PL}** through **S–S^{PL}**) compared with DEHP at a heating rate of 10 °C/min in nitrogen.

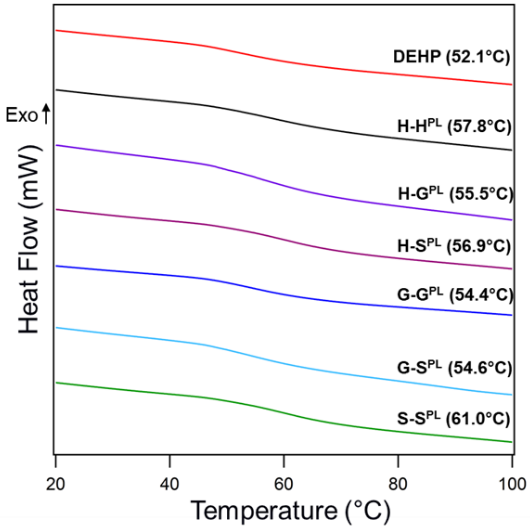

Figure S11. Differential scanning calorimetry of PVC with 10 wt% DEH-BPDA derivatives (**H–H^{PL}** through **S–S^{PL}**) compared with DEHP at a heating rate of 10 °C/min in nitrogen.

Table S16. Thermal properties of PVC plasticized with 10 wt% DEH-BPDA derivatives (H–H^{PL} through S–S^{PL}) and DEHP.

Polymer	T_g (°C)	T_{d10} (°C)[a]	T_{d50} (°C)[a]	Char Yield (%)
**PVC**	83.0	272	311	6.5
**PVC + DEHP**	52.1	253	328	9.0
**PVC + H–H^{PL}**	57.8	270	325	7.4
**PVC + H–G^{PL}**	55.5	278	329	6.1
**PVC + H–S^{PL}**	56.9	271	309	5.8
**PVC + G–G^{PL}**	54.4	281	327	6.0
**PVC + G–S^{PL}**	54.6	270	329	8.2
**PVC + S–S^{PL}**	61.0	273	326	7.3

[a] T_{d10} corresponds to the temperature at which 10% mass loss is observed. [b] T_{d50} corresponds to the temperature at which 50% mass loss is observed.

Toxicity predictions from EPA tools

Toxicity predictions were obtained from the EPA Toxicity Estimation Software Tool (TEST)[9] and the human metabolism and environmental breakdown products were estimated with the EPA Chemical Transformation Simulator (CTS).[10] We created in-house scripts in Python to allow high-throughput workflow and analysis.

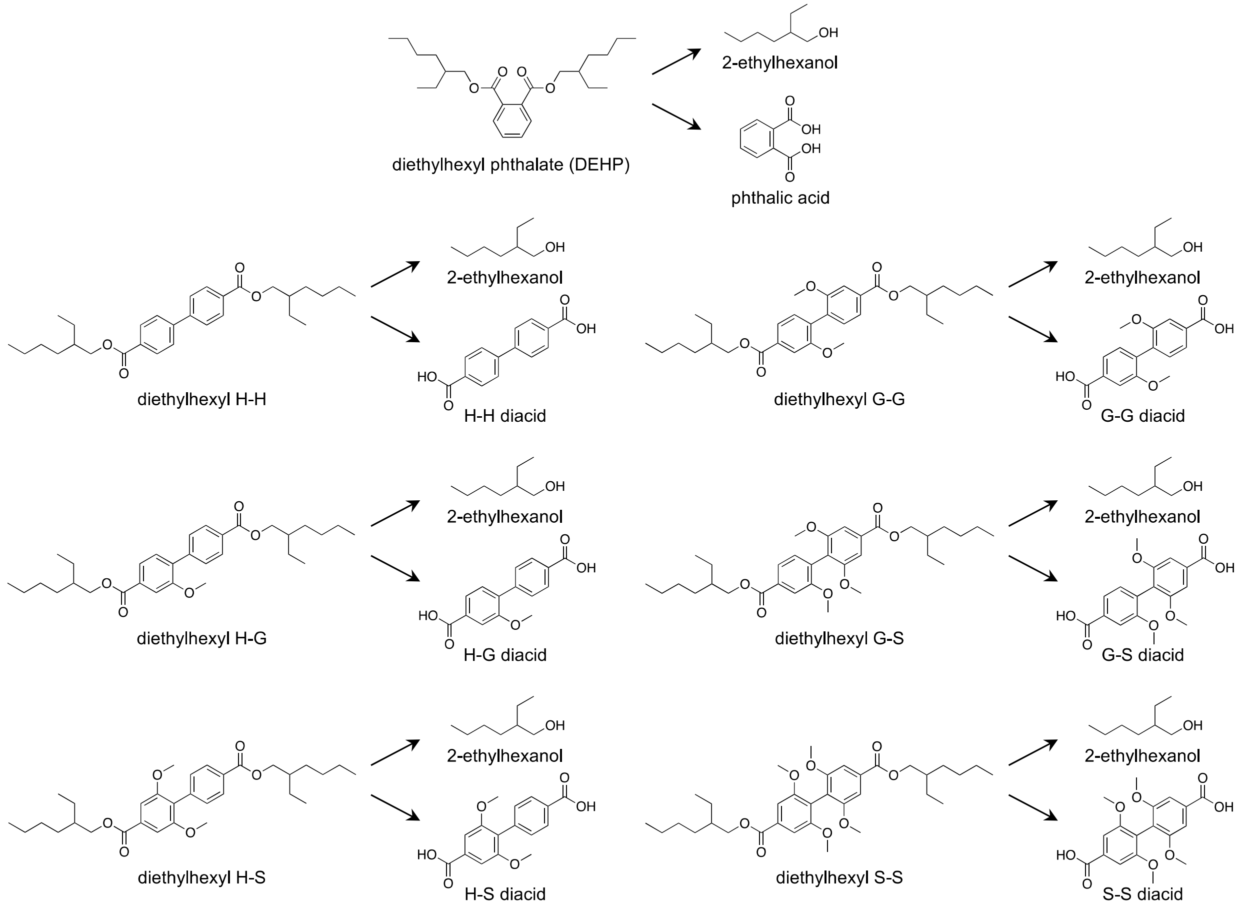

Figure S12. Predicted metabolic and environmental transformations of plasticizers

All plasticizers in this study were predicted by the EPA Chemical Transformation System to hydrolyse to their component diacids and alcohols, both in the environment via abiotic hydrolysis and in human via phase I metabolism. Toxicity predictions for these compounds in blue are provided in Table S16. None of these compounds were predicted to further react in the environment via abiotic means, while 2-ethylhexanol and all diacids except for phthalic acid were predicted to be further metabolized in humans (Figure S13).

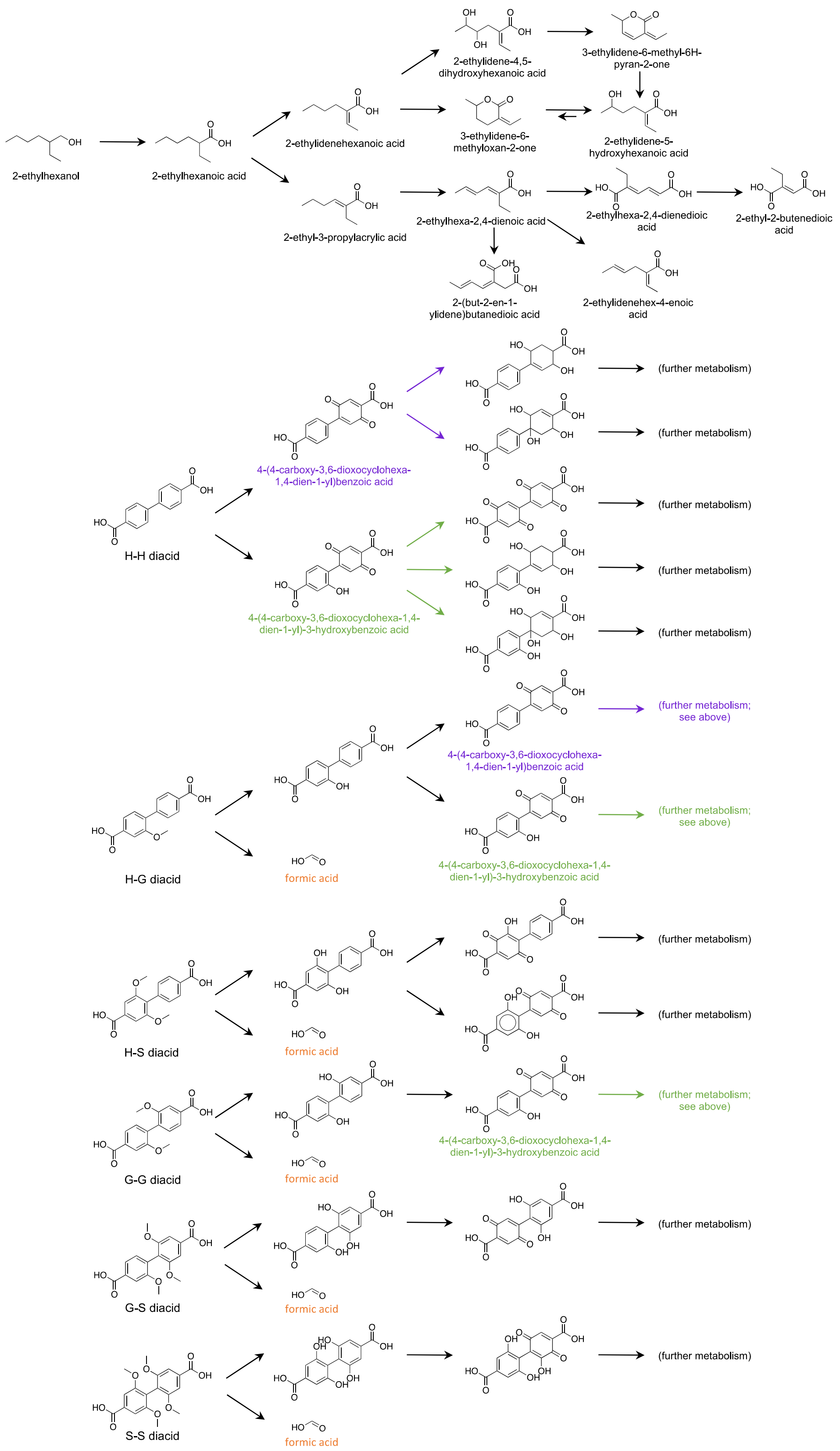

Figure S13. Predicted metabolites from 2-ethylhexanol and diacids. Main metabolic pathways predicted by the EPA Chemical Transformation System. Colors are used to highlight metabolites common to multiple diacids.

Table S17. Summary of EPA T.E.S.T. predictions.

	Tests for human health impacts			Tests for environmental impact			
	Develop. Toxicity	Ames Mutagen	Rat LD_{50} mg/kg**	Bioconcent. Factor	Minnow LC_{50} mg/L	*Daphnia* LC_{50} mg/L	*T. pyriformis* IGC_{50} mg/L
**Methylated Dimers**							
H-H			7,270	16	0.98	6.3	9.4
H-G			9,500	15	0.99	5.0	9.3
H-S			10,400	20	0.91	5.0	9.8
G-G			10,400	20	0.93	5.1	14
G-S			584	14	1.0	6.3	12
S-S			296	16	0.85	11	13
**Plasticizers**							
DEHP			31,000	18	0.24	1.6	0.09
DEH H-H			36,200	37	0.01	0.38	0.05
DEH H-G			38,600	36	0.01	0.34	0.05
DEH H-S			40,900	37	0.00	0.33	0.05
DEH G-G			44,800	37	0.00	0.32	0.05
DEH G-S			47,400	37	0.00	0.31	0.05
DEH S-S			36,200	38	0.00	0.32	0.06
**Oxoalcohol and Diacids**							
2-ethylhexanol			1,720	20	31	31	31
phthalic acid			5,130	0.5	30	30	30
H-H diacid			3,050	2.7	N/A	27	73
H-G diacid			3,430	2.1	N/A	17	82
H-S diacid			3,020	3.6	N/A	8.3	87
G-G diacid			3,240	2.8	N/A	5.6	87
G-S diacid			2,640	3.8	N/A	18	178
S-S diacid			3,010	3.6	N/A	6.8	N/A

Output from the Environmental Protection Agency (EPA) toxicity estimation software tool (T.E.S.T.) provided predicted results for experimental tests. The tests for developmental toxicity and Ames mutagenicity have either positive or negative outcomes, reported by the tool as true or false, and negative/false is the desired outcome corresponding to the lowest hazard category (see Table S18). Each cell is colored according to the corresponding color for the hazard category shown in Table S18, with green corresponding to the lowest hazard and red to the highest. "N/A" (colored gray) indicates that the EPA tool did not provide a prediction, which occurs when there is insufficient training data for a confident prediction. *Brief definitions of the hazard test names*: the bioconcentration factor is defined as the ratio of the chemical concentration in fish to that in water at steady state, and thus lower values correspond to lower hazard. Higher values correspond to lower hazard for the remaining categories: oral rat 50 percent lethal dose (LD_{50}), fathead minnow 50 percent lethal concentration (LC_{50}) after 96 hours of exposure, *D. magna* LC_{50} after 48 hours, and 50 percent growth inhibition concentration (IGC_{50}) after 48 hours for *T. pyriformis*. Colors correspond to the hazard levels shown in Table S18. *Abbreviations:* the *methylated dimers* **H–H** through **S–S** correspond to the coupling products created from methylated 4-hydroxy benzoic acid (**H**), vanillic acid (**G**), and syringic acid (**S**) monomers. *Plasticizers*: each diethylhexyl (DEH) structure is shown as the parent molecules in Figure S12. *Oxoalcohol and diacids*: these structures are the product molecules shown in Figure S12.

Table S18. Hazard classifications for EPA T.E.S.T. predictions

Hazard Indicators	I (highest)	II	III	IV (lowest)
Developmental toxin	positive			negative
Ames Mutagenicity	positive			negative
Oral Rat LD_{50}[a]	≤ 50 mg/kg	50 to 500 mg/kg	500 to 5,000	>5,000 mg/kg
Bioconcentration Factor[b]	≥ 5000	1000 to 5000		< 1000
96 hour fathead minnow LC_{50}[c]	≤1 mg/L	1 to ≤10 mg/L	>10 to 100 mg/L	> 100 mg/L
48 hour *D. magna* LC_{50}[c]				
48 hour *T. pyriformis* IGC_{50}[c]				

[a] *Protection of Environment*; Code of Federal Regulations, Title 40, Chapter I, Subchapter E, Part 156, Subpart D, § 156.62. [b] United Nations, *Globally harmonized system of classification and labelling of chemical (GHS), sixth revised edition*; ST/SG/AC.10/30/Rev.6; 2015. [c] United States Environmental Protection Agency, *Persistent Bioaccumulative Toxic (PBT) Chemicals; Lowering of Reporting Thresholds for Certain PBT Chemicals; Addition of Certain PBT Chemicals; Community Right-to-Know Toxic Chemical Reporting*; 40 CFR Part 372; 1999.

8. *Compound Characterization Data*

methyl 4-((methylsulfonyl)oxy)benzoate (**H–OMs**)

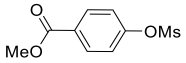

From methyl 4-hydroxybenzoate (7.6 g, 50 mmol) and methanesulfonyl chloride (9.9 mL, 60 mmol), the title compound was prepared following **GP 1** as a pale-yellow powder (11.1 g, 96% yield). The spectroscopic data matched those reported in the literature.[11]

^{1}H NMR (400 MHz, $CDCl_3$) δ 8.15 – 8.07 (m, 2H), 7.40 – 7.32 (m, 2H), 3.93 (s, 3H), 3.19 (s, 3H).

^{13}C NMR (101 MHz, $CDCl_3$) δ 165.9, 152.4, 131.7, 129.2, 121.9, 52.4, 37.8.

HRMS (ESI$^+$) Calc: $[M+H]^+$ ($C_9H_{11}O_5S$) 231.0322; measured: 231.0321 = 0.4 ppm difference.

methyl 4-(tosyloxy)benzoate (**H–OTs**)

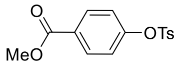

From methyl 4-hydroxybenzoate (7.6 g, 50 mmol) and *p*-toluenesulfonyl chloride (11.4 g, 60 mmol), the title compound was prepared following **GP 1** as a white powder (14 g, 93% yield). The spectroscopic data matched those reported in the literature.[11]

^{1}H NMR (400 MHz, $CDCl_3$) δ 8.02 – 7.94 (m, 2H), 7.74 – 7.67 (m, 2H), 7.35 – 7.28 (m, 2H), 7.10 – 7.02 (m, 2H), 3.90 (s, 3H), 2.45 (s, 3H).

^{13}C NMR (101 MHz, $CDCl_3$) δ 166.4, 153.0, 145.8, 132.0, 131.3, 129.9, 128.9, 128.5, 122.4, 52.3, 21.7.

HRMS (ESI$^+$) Calc: $[M+H]^+$ ($C_{15}H_{15}O_5S$) 307.0635; measured: 307.0630 = 1.6 ppm difference.

methyl 3-methoxy-4-((methylsulfonyl)oxy)benzoate (**G–OMs**)

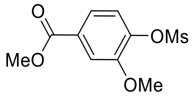

From methyl 4-hydroxy-3-methoxybenzoate (9.1 g, 50 mmol) and methanesulfonyl chloride (9.9 mL, 60 mmol), the title compound was prepared following **GP 1** as a white powder (12.2 g, 94% yield). The spectroscopic data matched those reported in the literature.[12]

^{1}H NMR (600 MHz, $CDCl_3$) δ 7.71 – 7.65 (m, 2H), 7.37 (d, J = 8.1 Hz, 1H), 3.96 (s, 3H), 3.93 (s, 3H), 3.22 (s, 3H).

^{13}C NMR (101 MHz, $CDCl_3$) δ 166.0, 151.4, 141.7, 130.1, 124.4, 122.8, 114.0, 56.3, 52.5, 38.7.

HRMS (ESI$^+$) Calc: $[M+H]^+$ ($C_{10}H_{13}O_6S$) 261.0427; measured: 261.0426 = 0.4 ppm difference.

methyl 3-methoxy-4-(tosyloxy)benzoate (**G–OTs**)

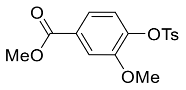

From methyl 4-hydroxy-3-methoxybenzoate (9.1 g, 50 mmol) and *p*-toluenesulfonyl chloride (11.4 g, 60 mmol), the title compound was prepared following **GP 1** as a white powder (16 g, 95% yield). The spectroscopic data matched those reported in the literature.[12]

^{1}H NMR (400 MHz, $CDCl_3$) δ 7.79 – 7.71 (m, 2H), 7.60 (dd, *J* = 8.4, 2.0 Hz, 1H), 7.51 (d, *J* = 1.9 Hz, 1H), 7.30 (d, *J* = 7.9 Hz, 2H), 7.22 (d, *J* = 8.4 Hz, 1H), 3.91 (s, 3H), 3.62 (s, 3H), 2.45 (s, 3H).

^{13}C NMR (101 MHz, $CDCl_3$) δ 166.1, 151.7, 145.3, 141.9, 133.0, 129.8, 129.5, 128.6, 123.9, 122.3, 113.6, 55.8, 52.4, 21.7.

HRMS (ESI$^+$) Calc: $[M+H]^+$ ($C_{16}H_{17}O_6S$) 337.0740; measured: 337.0736 = 1.2 ppm difference.

methyl 3-methoxy-4-(((trifluoromethyl)sulfonyl)oxy)benzoate (**G–OTf**)

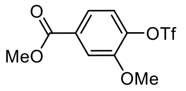

From methyl 4-hydroxy-3-methoxybenzoate (9.1 g, 50 mmol) and trifluoromethanesulfonic anhydride (10.1 mL, 60 mmol), the title compound was prepared following **GP 1** as a yellow liquid (14.4 g, 92% yield). The spectroscopic data matched those reported in the literature.[1]

^{1}H NMR (400 MHz, $CDCl_3$) δ 7.72 (d, *J* = 1.9 Hz, 1H), 7.68 (dd, *J* = 8.4, 1.9 Hz, 1H), 7.28 (d, *J* = 8.5 Hz, 1H), 3.98 (s, 3H), 3.94 (s, 3H).

^{13}C NMR (101 MHz, $CDCl_3$) δ 165.6, 151.3, 141.8, 131.1, 122.8, 122.4, 120.3, 117.1, 114.2, 56.4, 52.6.

HRMS (ESI$^+$) Calc: $[M+H]^+$ ($C_{10}H_{10}F_3O_6S$) 315.0145; measured: 315.0141 = 1.3 ppm difference.

methyl 3,5-dimethoxy-4-((methylsulfonyl)oxy)benzoate (**S–OMs**)

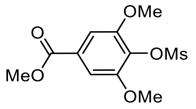

From methyl 4-hydroxy-3,5-dimethoxybenzoate (10.6 g, 50 mmol) and methanesulfonyl chloride (9.9 mL, 60 mmol), the title compound was prepared following **GP 1** as a white powder (13 g, 89% yield).

^{1}H NMR (400 MHz, $CDCl_3$) δ 7.34 (s, 2H), 3.95 (s, 6H), 3.93 (s, 3H), 3.33 (s, 3H).

^{13}C NMR (101 MHz, $CDCl_3$) δ 166.1, 153.1, 131.6, 129.1, 106.4, 56.5, 52.6, 40.2.

HRMS (ESI$^+$) Calc: $[M+NH_4]^+$ ($C_{11}NH_{18}O_7S$) 308.0799; measured: 308.0796 = 1.0 ppm difference.

methyl 3,5-dimethoxy-4-(tosyloxy)benzoate (**S–OTs**)

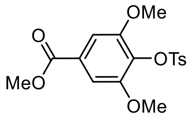

From methyl 4-hydroxy-3,5-dimethoxybenzoate (10.6 g, 50 mmol) and *p*-toluenesulfonyl chloride (11.4 g, 60 mmol), the title compound was prepared following **GP 1** as a white powder (16.5 g, 90% yield).

^{1}H NMR (400 MHz, $CDCl_3$) δ 7.89 – 7.82 (m, 2H), 7.34 (d, *J* = 8.0 Hz, 2H), 7.26 (s, 2H), 3.91 (s, 3H), 3.73 (s, 6H), 2.46 (s, 3H).

^{13}C NMR (101 MHz, $CDCl_3$) δ 166.1, 153.3, 144.8, 134.7, 131.6, 129.2, 129.0, 128.4, 106.3, 56.2, 52.5, 21.7.

HRMS (ESI$^+$) Calc: $[M+NH_4]^+$ ($C_{17}NH_{22}O_7S$) 384.1112; measured: 384.1110 = 0.5 ppm difference.

methyl 3,5-dimethoxy-4-(((trifluoromethyl)sulfonyl)oxy)benzoate (**S–OTf**)

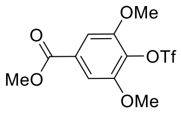

From methyl 4-hydroxy-3,5-dimethoxybenzoate (10.6 g, 50 mmol) and trifluoromethanesulfonic anhydride (10.1 mL, 60 mmol), the title compound was prepared following **GP 1** as a white powder (16 g, 93% yield).

^{1}H NMR (400 MHz, $CDCl_3$) δ 7.34 (s, 2H), 3.95 (s, 6H), 3.94 (s, 3H).

^{13}C NMR (101 MHz, $CDCl_3$) δ 165.8, 152.3, 131.0, 130.2, 120.2, 117.0, 106.3, 56.6, 52.7.

HRMS (ESI$^+$) Calc: $[M+H]^+$ ($C_{11}H_{12}F_3O_7S$) 345.0250; measured: 345.0246 = 1.2 ppm difference.

dimethyl [1,1'-biphenyl]-4,4'-dicarboxylate (**H–H**)

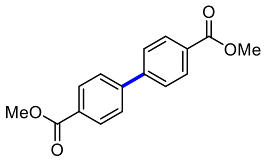

GP 2 was followed using **H–OMs** (460 mg, 2.0 mmol), $NiCl_2$(dme) (4.4 mg, 1 mol%), 2,2'-bipyridyl (9.4 mg, 3 mol%), which furnished the title compound as a white powder (262 mg, 97% yield). The spectroscopic data matched those reported in the literature.[13]

^{1}H NMR (400 MHz, $CDCl_3$) δ 8.13 (d, *J* = 8.2 Hz, 4H), 7.69 (d, *J* = 8.2 Hz, 4H), 3.95 (s, 6H).

^{13}C NMR (126 MHz, $CDCl_3$) δ 166.8, 144.4, 130.2, 129.7, 127.3, 52.2.

HRMS (ESI$^+$) Calc: $[M+H]^+$ ($C_{16}H_{15}O_4$) 271.0965; measured: 271.0973 = 0.7 ppm difference.

dimethyl 2,2'-dimethoxy-[1,1'-biphenyl]-4,4'-dicarboxylate (**G–G**)

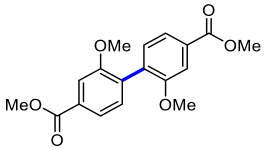

GP 2 was followed using **G–OMs** (520 mg, 2.0 mmol), $NiCl_2$(dme) (22 mg, 5 mol%), 2,2'-bipyridyl (47 mg, 15 mol%), which furnished the title compound as a white powder (297 mg, 90% yield). The spectroscopic data matched those reported in the literature.[14]

^{1}H NMR (400 MHz, $CDCl_3$) δ 7.70 (dd, J = 7.8, 1.6 Hz, 2H), 7.64 (d, J = 1.5 Hz, 2H), 7.30 (d, J = 7.8 Hz, 2H), 3.94 (s, 6H), 3.83 (s, 6H).

^{13}C NMR (126 MHz, $CDCl_3$) δ 166.9, 156.9, 131.8, 131.1, 130.9, 121.8, 111.9, 55.4, 52.2.

HRMS (ESI$^+$) Calc: $[M+H]^+$ ($C_{18}H_{19}O_6$) 331.1176; measured: 331.1174 = 0.6 ppm difference.

dimethyl 2,2',6,6'-tetramethoxy-[1,1'-biphenyl]-4,4'-dicarboxylate (**S–S**)

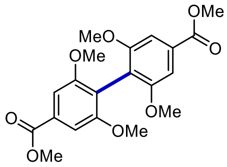

GP 3 was followed to furnish the title compound as a pale-yellow powder (293 mg, 75% yield).

^{1}H NMR (400 MHz, $CDCl_3$) δ 7.34 (s, 4H), 3.94 (s, 6H), 3.77 (s, 12H).

^{13}C NMR (151 MHz, $CDCl_3$) δ 167.0, 158.5, 131.0, 116.9, 105.6, 56.2, 52.2.

HRMS (ESI$^+$) Calc: $[M+H]^+$ ($C_{20}H_{23}O_8$) 391.1387; measured: 391.1382 = 1.3 ppm difference.

dimethyl 2-methoxy-[1,1'-biphenyl]-4,4'-dicarboxylate (**H–G**)

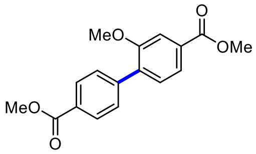

GP 4 at 60 ºC was followed using **H–OMs** (460 mg, 2.0 mmol), **G–OTs** (841 mg, 2.5 mmol), $NiCl_2$(dme) (44 mg, 10 mol%), 4,4'-dPhbpy (74 mg, 12 mol%), $PdCl_2(MeCN)_2$ (15.6 mg, 3 mol%), dppb (30.6 mg, 3.6 mol%), DMA (5 mL), a Ni foam cathode, which furnished the title compound as a white powder (420 mg, 70% yield). The spectroscopic data matched those reported in the literature.[15]

^{1}H NMR (400 MHz, $CDCl_3$) δ 8.12 – 8.05 (m, 2H), 7.72 (dd, J = 7.9, 1.6 Hz, 1H), 7.66 (d, J = 1.5 Hz, 1H), 7.64 – 7.59 (m, 2H), 7.39 (d, J = 7.9 Hz, 1H), 3.95 (s, 3H), 3.94 (s, 3H), 3.88 (s, 3H).

^{13}C NMR (101 MHz, $CDCl_3$) δ 167.0, 166.8, 156.4, 142.2, 134.1, 131.0, 130.7, 129.5, 129.4, 129.1, 122.3, 112.1, 55.8, 52.3, 52.2.

HRMS (ESI$^+$) Calc: $[M+H]^+$ ($C_{17}H_{17}O_5$) 301.1071; measured: 301.1071 < 0.1 ppm difference.

dimethyl 2,6-dimethoxy-[1,1'-biphenyl]-4,4'-dicarboxylate (**H–S**)

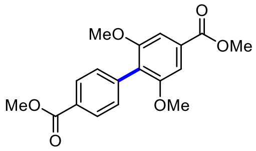

GP 4 at 80 ºC was followed using **H–OTs** (612 mg, 2.0 mmol), **S–OTf** (688 mg, 2.0 mmol), $NiCl_2$(dme) (44 mg, 10 mol%), phen (43 mg, 12 mol%), $PdCl_2(MeCN)_2$ (10.4 mg, 2 mol%), SPhos (33 mg, 4 mol%), DMSO (5 mL), a RVC cathode, which furnished the title compound as a white powder (468 mg, 71% yield).

^{1}H NMR (400 MHz, $CDCl_3$) δ 8.12 – 8.05 (m, 2H), 7.45 – 7.38 (m, 2H), 7.34 (s, 2H), 3.96 (s, 3H), 3.93 (s, 3H), 3.79 (s, 6H).

^{13}C NMR (151 MHz, $CDCl_3$) δ 167.1, 166.8, 157.3, 138.5, 131.0, 130.8, 129.0, 128.9, 123.0, 105.4, 56.1, 52.4, 52.1.

HRMS (ESI$^+$) Calc: $[M+H]^+$ ($C_{18}H_{19}O_5$) 331.1176; measured: 331.1172 = 1.2 ppm difference.

dimethyl 2,2',6-trimethoxy-[1,1'-biphenyl]-4,4'-dicarboxylate (**G–S**)

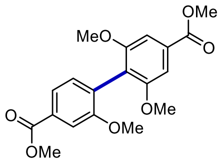

GP 4 at 80 ºC was followed using **G–OTs** (841 mg, 2.5 mmol), **S–OTf** (688 mg, 2.0 mmol), $NiCl_2$(dme) (44 mg, 10 mol%), phen (43 mg, 12 mol%), $PdCl_2(MeCN)_2$ (15.6 mg, 3 mol%), SPhos (49 mg, 6 mol%), DMSO (5 mL), a Ni foam cathode, which furnished the title compound as a white powder (504 mg, 70% yield).

^{1}H NMR (400 MHz, $CDCl_3$) δ 7.71 (dd, J = 7.8, 1.6 Hz, 1H), 7.65 (d, J = 1.6 Hz, 1H), 7.34 (s, 2H), 7.22 (d, J = 7.8 Hz, 1H), 3.95 (s, 3H), 3.93 (s, 3H), 3.80 (s, 3H), 3.78 (s, 6H).

^{13}C NMR (151 MHz, $CDCl_3$) δ 167.1, 166.9, 157.7, 157.3, 131.8, 131.0, 130.7, 128.1, 121.7, 120.1, 111.9, 105.4, 56.2, 56.0, 52.3, 52.2.

HRMS (ESI$^+$) Calc: $[M+H]^+$ ($C_{19}H_{21}O_7$) 361.1282; measured: 361.1278 = 1.1 ppm difference.

Bis(2-ethylhexyl) phthalate (**DEHP**)

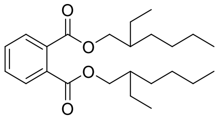

From phthalic anhydride (500 mg, 1 equiv) and 2-ethyl hexanol (2.41 g, 10 equiv), using methane sulfonic acid (1.5 wt%) instead of titanium butoxide, the title compound was prepared following **GP 5** at 130 °C as a transparent viscous oil (1.05 g, 80% yield).

^{1}H NMR (400 MHz, $CDCl_3$, ppm): δ 7.71 (dd, *J* = 5.6, 3.2 Hz, 2H), 7.53 (dd, *J* = 5.6, 3.2 Hz, 2H), 4.26 – 4.18 (m, 4H), 1.71 – 1.65 (m, 2H), 1.43 – 1.28 (m, 16H), 0.94-0.88 (m, 12H).

^{13}C NMR (101 MHz, $CDCl_3$, ppm): δ 11.5, 14.6, 23.5, 24.3, 29.4, 30.9, 39.2, 68.7, 129.3, 131.4, 133.0, 168.3.

HRMS (ESI$^+$) Calc: $[2M+Na]^+$ ($C_{48}H_{76}O_8Na$) 803.5432; measured: 803.5422 = 1.2 ppm difference.

Bis(2-ethylhexyl)[1,1’-biphenyl]-4,4’-dicarboxylate **(H–H^{PL})**

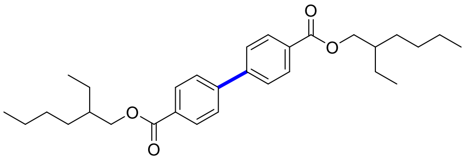

From dimethyl [1,1'-biphenyl]-4,4'-dicarboxylate (500 mg, 1 equiv) and 2-ethyl hexanol (2.41 g, 10 equiv), the title compound was prepared following **GP 5** as a translucent viscous oil (794 mg, 92% yield).

^{1}H NMR (400 MHz, $CDCl_3$, ppm): δ 8.13 (d, *J* = 8.4 Hz, 2H), 7.69 (d, *J* = 8.4 Hz, 2H), 4.31 – 4.23 (m, 4H), 1.77 – 1.71 (m, 2H), 1.50 – 1.33 (m, 16H), 0.964 (t, *J* = 7.2 Hz, 6H), 0.913 (t, *J* = 6.8 Hz, 6H).

^{13}C NMR (101 MHz, $CDCl_3$, ppm): δ 11.2, 14.0, 23.0, 24.0, 29.0, 30.6, 38.9, 67.4, 127.2, 130.1, 144.3, 166.4.

HRMS (ESI$^+$) Calc: $[M+H]^+$ ($C_{30}H_{43}O_4$) 467.3156; measured: 467.3149 = 1.5 ppm difference.

Bis(2-ethylhexyl) 2-methoxy-[1,1’-biphenyl]-4,4’-dicarboxylate **(H–G^{PL})**

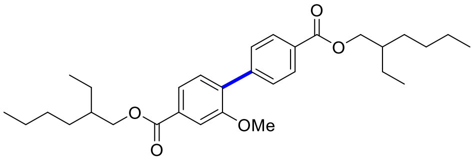

From dimethyl 2-methoxy-[1,1'-biphenyl]-4,4'-dicarboxylate (500 mg, 1 equiv) and 2-ethyl hexanol (2.17 g, 10 equiv), the title compound was prepared following **GP 5** as a translucent viscous oil (777 mg, 94% yield).

^{1}H NMR (400 MHz, $CDCl_3$, ppm): δ 8.09 (d, *J* = 11.3 Hz, 2H), 7.72 (dd, *J* = 2, 10.5 Hz, 1H), 7.68 (d, *J* = 1.8 Hz, 1H), 7.60 (d, *J* = 11.32 Hz, 2H), 7.38 (d, *J* = 10.4 Hz, 1H), 4.29 – 4.25 (m, 4H), 3.87 (s, 3H) 1.79 – 1.70 (m, 2H), 1.50 – 1.33 (m, 16H), 0.994 – 0.891 (m, 12H).

^{13}C NMR (101 MHz, $CDCl_3$, ppm): δ 11.07, 11.00, 14.02, 22.96, 23.98, 24.04, 28.98, 30.58, 30.62, 38.93, 55.71,67.25, 67.56, 112.05, 122.09, 129.36, 129.43, 129.52, 130.58, 131.39, 134.05, 142.13, 156.37, 166.36, 166.53.

HRMS (ESI$^+$) Calc: $[M+H]^+$ ($C_{31}H_{45}O_5$) 497.3262; measured: 497.3252 = 2.0 ppm difference.

Bis(2-ethylhexyl) 2,6-dimethoxy-[1,1'-biphenyl]-4,4'-dicarboxylate **(H–S^{PL})**

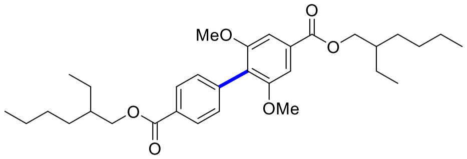

From dimethyl 2,6-dimethoxy-[1,1'-biphenyl]-4,4'-dicarboxylate (500 mg, 1 equiv) and 2-ethyl hexanol (1.97 g, 10 equiv), the title compound was prepared following **GP 5** as a translucent viscous oil (733 mg, 92% yield).

^{1}H NMR (400 MHz, $CDCl_3$, ppm): δ 8.12 – 8.05 (m, 2H), 7.45 – 7.38 (m, 2H), 7.35 (s, 2H), 4.29-4.25 (m, 4H), 3.79 (s, 6H), 1.79 – 1.69 (m, 2H), 1.49 – 1.29 (m, 16H), 0.994 – 0.954 (m, 12H).

^{13}C NMR (101 MHz, $CDCl_3$, ppm): δ 11.06, 14.58, 23.52, 24.52, 24.68, 29.54, 29.54, 31.14, 31.25, 39.48, 56.53, 65.85, 67.63, 68.31, 105.84, 123.46, 129.48, 129.81, 131.25, 131.92, 138.88, 157.85, 166.85, 167.22.

HRMS (ESI$^+$) Calc: $[M+H]^+$ ($C_{32}H_{47}O_6$) 527.3367; measured: 527.3357 = 1.9 ppm difference.

Bis(2-ethylhexyl) 2,2'-dimethoxy-[1,1'-biphenyl]-4,4'-dicarboxylate **(G–G^{PL})**

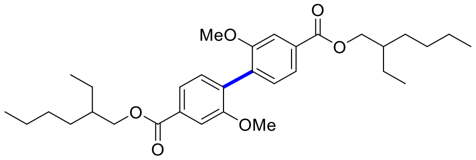

From dimethyl 2,2'-dimethoxy-[1,1'-biphenyl]-4,4'-dicarboxylate (500 mg, 1 equiv) and 2-ethyl hexanol (1.97 g, 10 equiv), the title compound was prepared following **GP 5** as a translucent viscous oil (741 mg, 93% yield).

^{1}H NMR (400 MHz, $CDCl_3$, ppm): δ 7.72 (dd, *J* = 2, 10.4 Hz, 2H), 7.66 (d, *J* = 1.8 Hz, 2H), 7.29 (d, *J* = 10.4 Hz, 2H), 4.28 – 4.26 (m, 4H), 3.83 (s, 6H), 1.78 – 1.68 (m, 2H), 1.53 – 1.33 (m, 16H), 0.990 – 0.893 (m, 12H).

^{13}C NMR (101 MHz, $CDCl_3$, ppm): δ 11.1, 14.1, 23.0, 24.1, 29.0, 30.7, 39.0, 55.8, 67.5, 111.9, 121.7, 131.1, 131.3, 131.7, 156.9, 166.6.

HRMS (ESI$^+$) Calc: $[M+H]^+$ ($C_{32}H_{47}O_6$) 527.3367; measured: 527.3358 = 1.7 ppm difference.

Bis(2-ethylhexyl) 2,2’,6-trimethoxy-[1,1’-biphenyl]-4,4’-dicarboxylate **(G–S^PL^)**

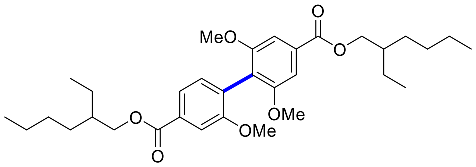

From dimethyl 2,2',6-trimethoxy-[1,1'-biphenyl]-4,4'-dicarboxylate (500 mg, 1 equiv) and 2-ethyl hexanol (1.81 g, 10 equiv), the title compound was prepared following **GP 5** as a translucent viscous oil (695 mg, 90% yield).

^{1}H NMR (400 MHz, $CDCl_3$, ppm): δ 7.70 (d, *J* = 7.8 Hz, 1H), 7.67 (d, *J* = 1.6 Hz, 1H), 7.35 (s, 2H), 7.22 (d, *J* = 7.8 Hz, 1H), 4.28 – 4.25 (m, 4H), 3.81 (s, 3H), 3.77 (s, 6H), 1.79 – 1.70 (m, 2H), 1.49 – 1.32 (m, 16H), 0.991 – 0.959 (m, 6H), 0.940 – 0.899 (m, 6H).

^{13}C NMR (101 MHz, $CDCl_3$, ppm): δ 11.1, 14.0, 23.0, 24.0, 24.1, 29.0, 30.6, 30.7, 39.0, 55.9, 56.1, 67.2, 67.6, 105.3, 111.9, 120.0, 121.6, 128.0, 131.1, 131.4, 131.7, 157.2, 157.7, 166.5, 166.7.

HRMS (ESI$^+$) Calc: $[M+H]^+$ ($C_{33}H_{49}O_7$) 557.3473; measured: 557.3469 = 0.7 ppm difference.

Bis(2-ethylhexyl) 2,2’,6,6’-tetramethoxy-[1,1’-biphenyl]-4,4’-dicarboxylate **(S–S^PL^)**

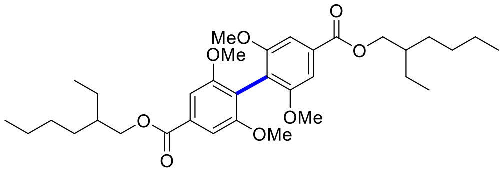

From dimethyl 2,2',6,6'-tetramethoxy-[1,1'-biphenyl]-4,4'-dicarboxylate (500 mg, 1 equiv) and 2-ethyl hexanol (1.67 g, 10 equiv), the title compound was prepared following **GP 5** as a slightly yellow viscous oil (677 mg, 90% yield).

^{1}H NMR (400 MHz, $CDCl_3$, ppm): δ 7.36 (s, 4H), 4.29 – 4.23 (m, 4H), 3.78 (s, 12H), 1.76 – 1.72 (m, 2H), 1.49 – 1.32 (m, 16H), 0.99 – 0.90 (m, 12H).

^{13}C NMR (101 MHz, $CDCl_3$, ppm): δ 166.6, 158.0, 131.4, 116.7, 105.5, 67.5, 56.2, 39.0, 30.8, 29.0, 24.2, 23.0, 14.1, 11.2.

HRMS (ESI$^+$) Calc: $[M+H]^+$ ($C_{34}H_{51}O_8$) 587.3579; measured: 587.3573 = 1.0 ppm difference.

9. *References*

1. Mori, A.; Mizusaki, T.; Ikawa, T.; Maegawa, T.; Monguchi, Y.; Sajiki, H. Mechanistic Study of a Pd/C-Catalyzed Reduction of Aryl Sulfonates Using the Mg–MeOH–NH_4OAc System. *Chem. Eur. J.* **2007**, *13*, 1432–1441.
2. Boehm, P.; Roediger, S.; Bismuto, A.; Morandi, B. Palladium-Catalyzed Chlorocarbonylation of Aryl (Pseudo)Halides Through In Situ Generation of Carbon Monoxide. *Angew. Chem. Int. Ed.* **2020**, *59*, 17887–17896.
3. Zhong, X.; Hoque, M. A.; Graaf, M. D.; Harper, K. C.; Wang, F.; Genders, J. D.; Stahl, S. S. Scalable Flow Electrochemical Alcohol Oxidation: Maintaining High Stereochemical Fidelity in the Synthesis of Levetiracetam. *Org. Process Res. Dev.* **2021**, *25*, 2601–2607.
4. Percec, V.; Bae, J.-Y.; Zhao, M.; Hill, D. H. Aryl Mesylates in Metal-Catalyzed Homocoupling and Cross-Coupling Reactions. 1. Functional Symmetrical Biaryls from Phenols via Nickel-Catalyzed Homocoupling of Their Mesylates. *J. Org. Chem.* **1995**, *60*, 176.
5. Zackasee, J. L. S.; Al Zubaydi, S.; Truesdell, B. L.; Sevov, C. S. Synergistic Catalyst–Mediator Pairings for Electroreductive Cross-Electrophile Coupling Reactions. *ACS Catal.* **2022**, 1161–1166.
6. Kawamata, Y.; Vantourout, J. C.; Hickey, D. P.; Bai, P.; Chen, L.; Hou, Q.; Qiao, W.; Barman, K.; Edwards, M. A.; Garrido-Castro, A. F.; deGruyter, J. N.; Nakamura, H.; Knouse, K.; Qin, C.; Clay, K. J.; Bao, D.; Li, C.; Starr, J. T.; Garcia-Irizarry, C.; Sach, N.; White, H. S.; Neurock, M.; Minteer, S. D.; Baran, P. S. Electrochemically Driven, Ni-Catalyzed Aryl Amination: Scope, Mechanism, and Applications. *J. Am. Chem. Soc.* **2019**, *141*, 6392–6402.
7. Skrzypek, J.; Lachowska, M.; Kulawska, M.; Moroz, H. Synthesis of Bis(2-Ethylhexyl) Phthalate over Methane Sulfonic Acid Catalyst. Kinetic Investigations. *React Kinet Catal Lett* **2008**, *93*, 281–286.
8. Gubbels, E.; Drijfhout, J. P.; Posthuma-van Tent, C.; Jasinska-Walc, L.; Noordover, B. A. J.; Koning, C. E. Bio-Based Semi-Aromatic Polyesters for Coating Applications. *Prog. Org. Coat.* **2014**, *77*, 277–284.
9. Martin, T. M. *T. E. S. T. (Toxicity Estimation Software Tool) Version 5.1*, 5.1; United State Environmental Protection Agency: **2020**.
10. EPA, U. S., Chemical Transformation Simulator (CTS), Version 1.0. *Chemical Transformation Simulator (CTS), Version 1.0* **2019**.
11. Wilson, D. A.; Wilson, C. J.; Moldoveanu, C.; Resmerita, A.-M.; Corcoran, P.; Hoang, L. M.; Rosen, B. M.; Percec, V. Neopentylglycolborylation of Aryl Mesylates and Tosylates Catalyzed by Ni-Based Mixed-Ligand Systems Activated with Zn. *J. Am. Chem. Soc.* **2010**, *132*, 1800–1801.
12. Yanagita, H.; Yamamoto, N.; Fuji, H.; Liu, X.; Ogata, M.; Yokota, M.; Takaku, H.; Hasegawa, H.; Odagiri, T.; Tashiro, M.; Hoshino, T. Mechanism of Drug Resistance of Hemagglutinin of Influenza Virus and Potent Scaffolds Inhibiting Its Function. *ACS Chem. Biol.* **2012**, *7*, 552–562.
13. Malineni, J.; Jezorek, R. L.; Zhang, N.; Percec, V. An Indefinitely Air-Stable σ-Ni^{II} Precatalyst for Quantitative Cross-Coupling of Unreactive Aryl Halides and Mesylates with Aryl Neopentylglycolboronates. *Synthesis* **2016**, *48*, 2795–2807.
14. Rankine, D.; Avellaneda, A.; Hill, M. R.; Doonan, C. J.; Sumby, C. J. Control of Framework Interpenetration for in Situ Modified Hydroxyl Functionalised IRMOFs. *Chem. Commun.* **2012**, *48*, 10328–10330.
15. Burrows, A. D.; Frost, C. G.; Mahon, M. F.; Richardson, C. Post-Synthetic Modification of Tagged Metal–Organic Frameworks. *Angew. Chem. Int. Ed.* **2008**, *47*, 8482–8486.

10. NMR Spectra

^{1}H NMR (400 MHz, $CDCl_3$) of **H–OMs**

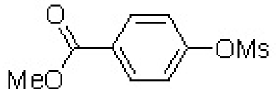

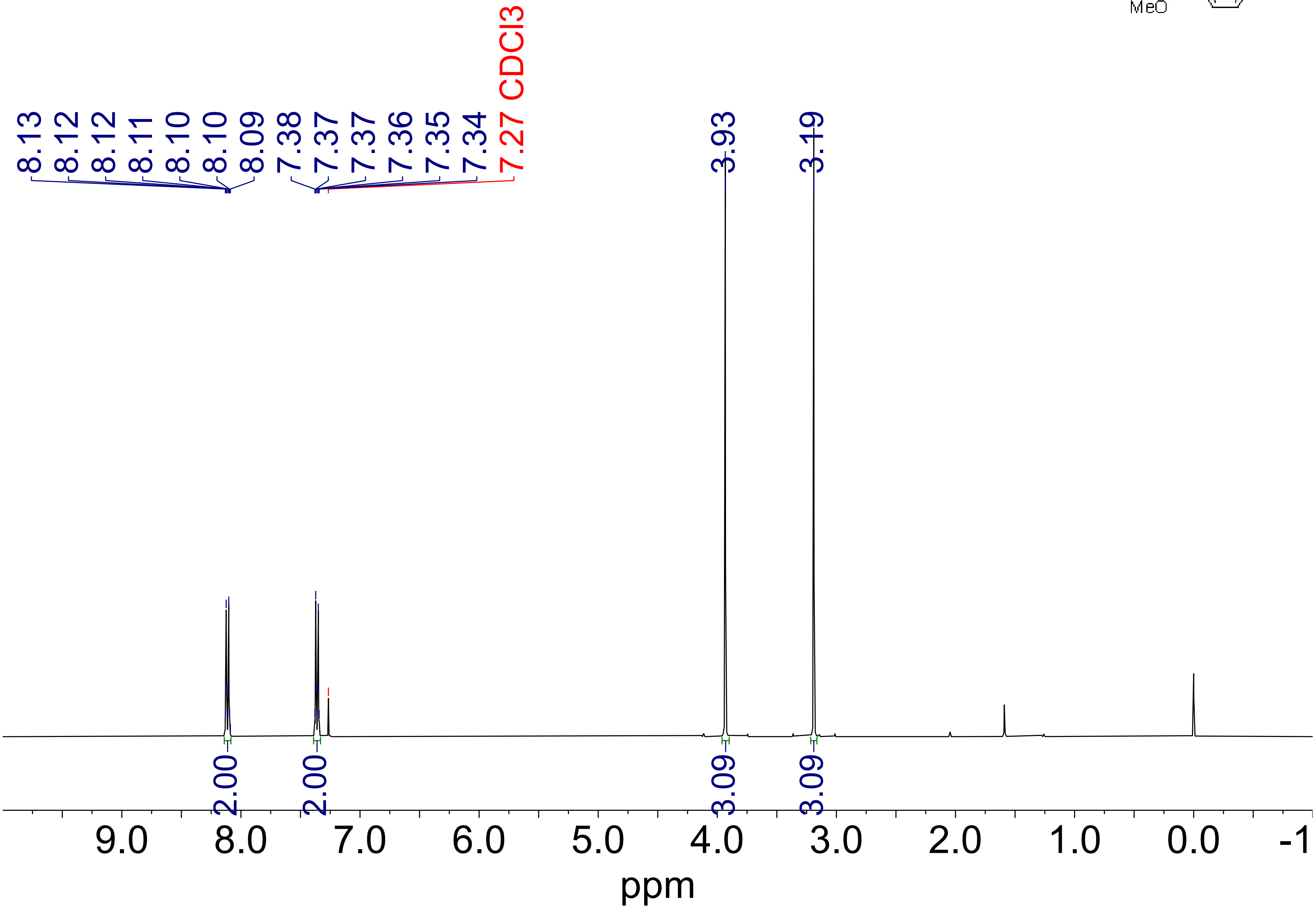

^{13}C NMR (100 MHz, $CDCl_3$) of **H–OMs**

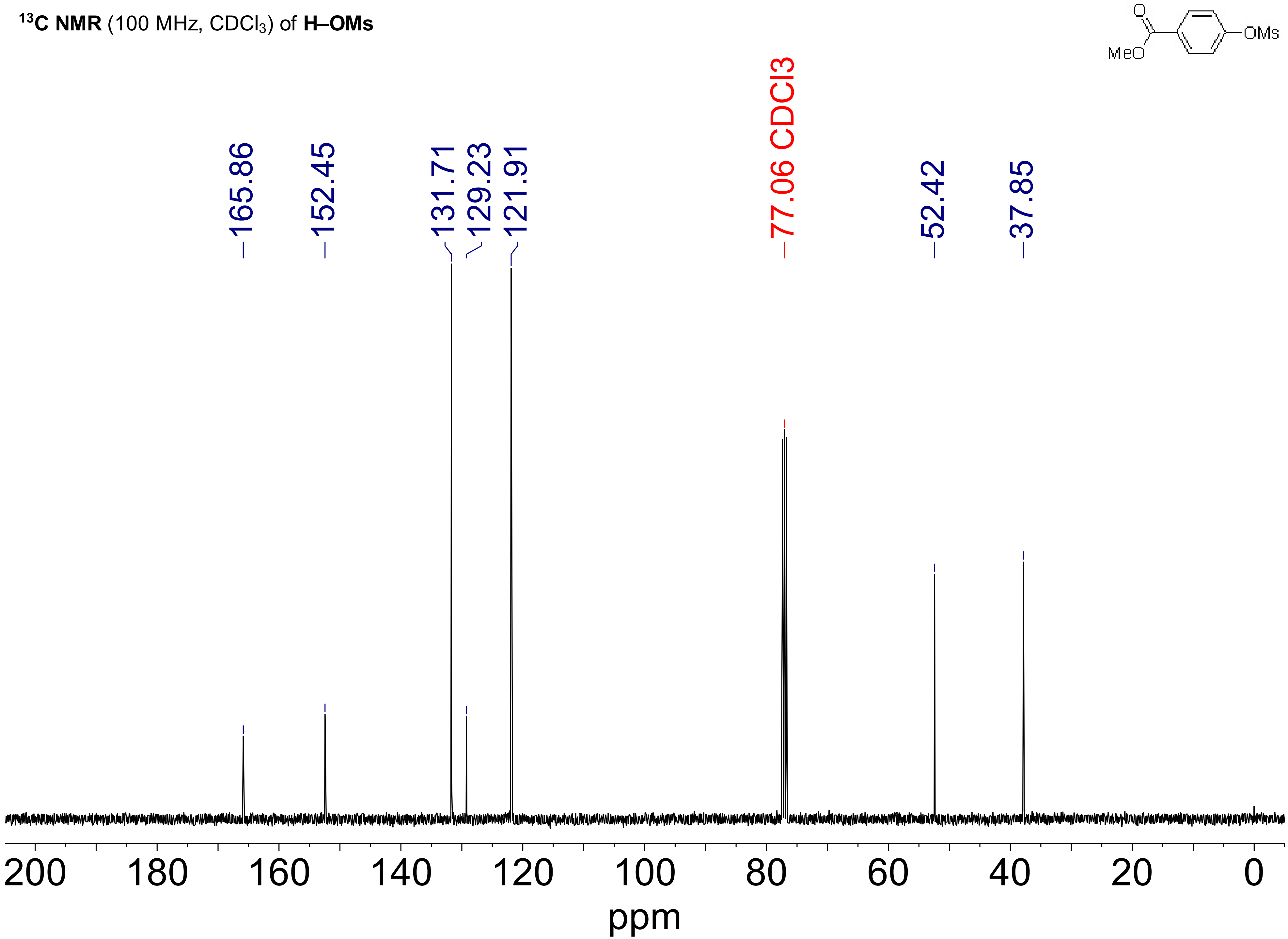

^{1}H NMR (600 MHz, $CDCl_3$) of **G–OMs**

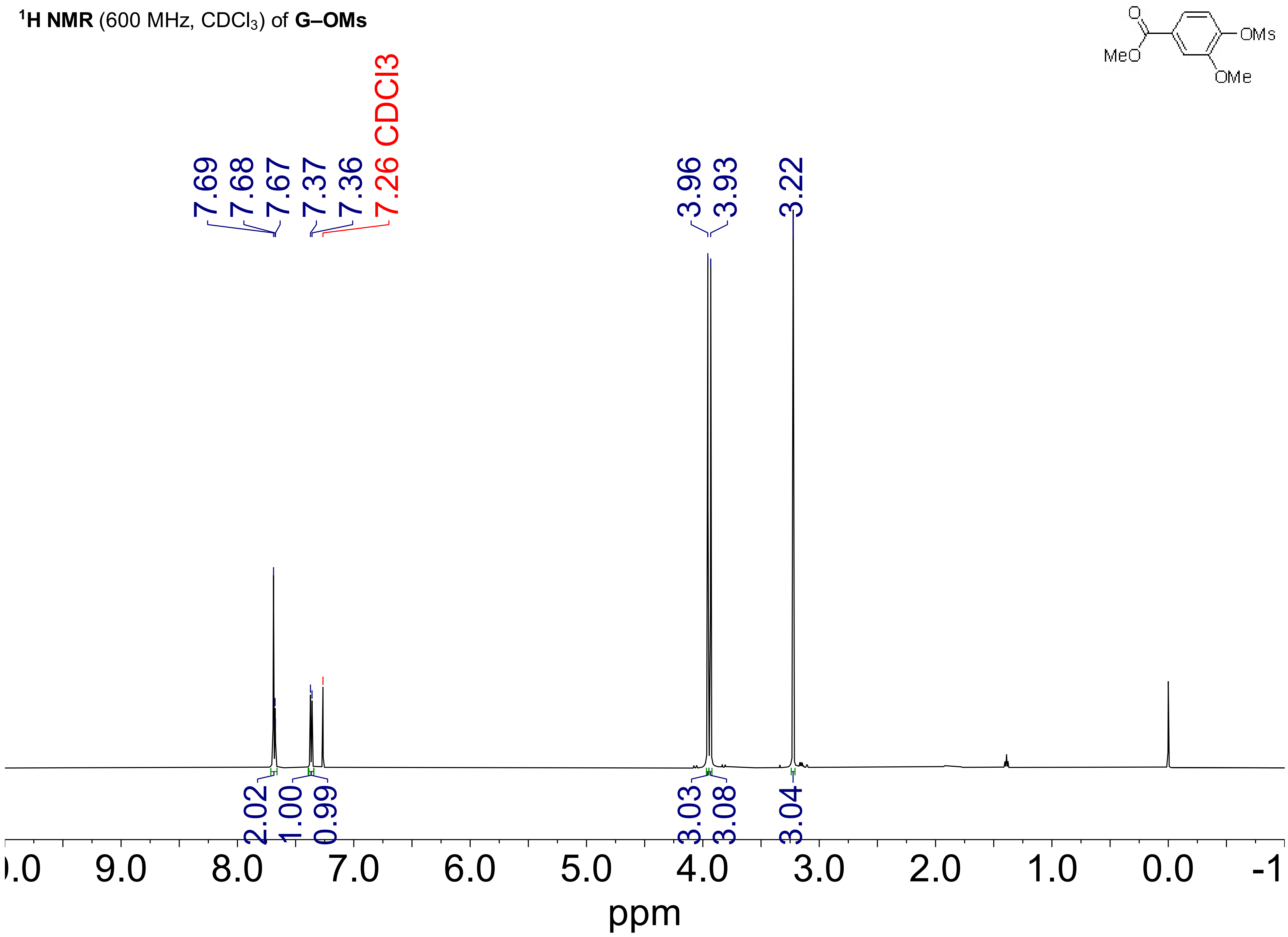

^{13}**C NMR** (100 MHz, $CDCl_3$) of **G–OMs**

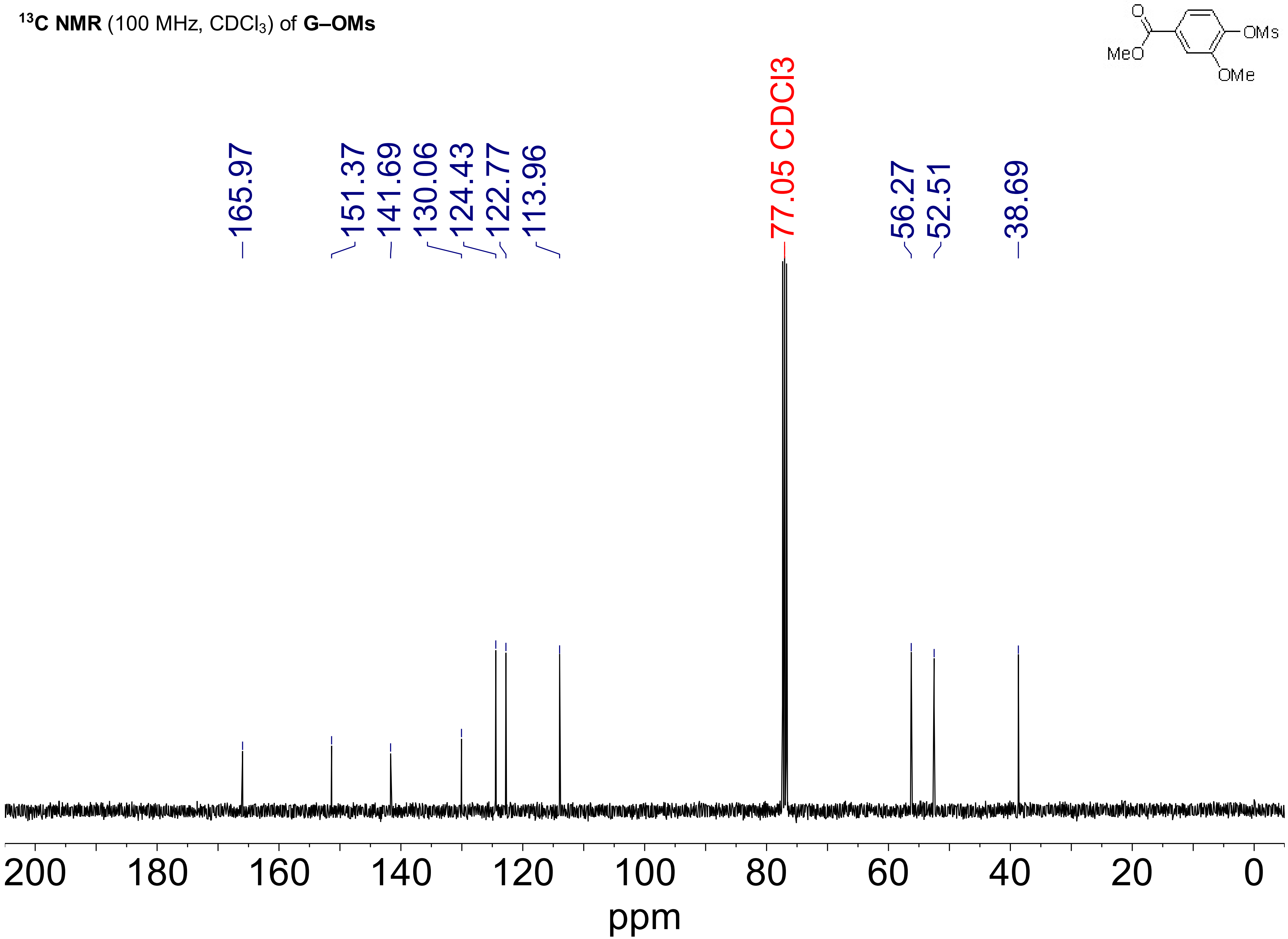

^{1}H NMR (400 MHz, $CDCl_3$) of **S–OMs**

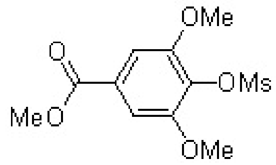

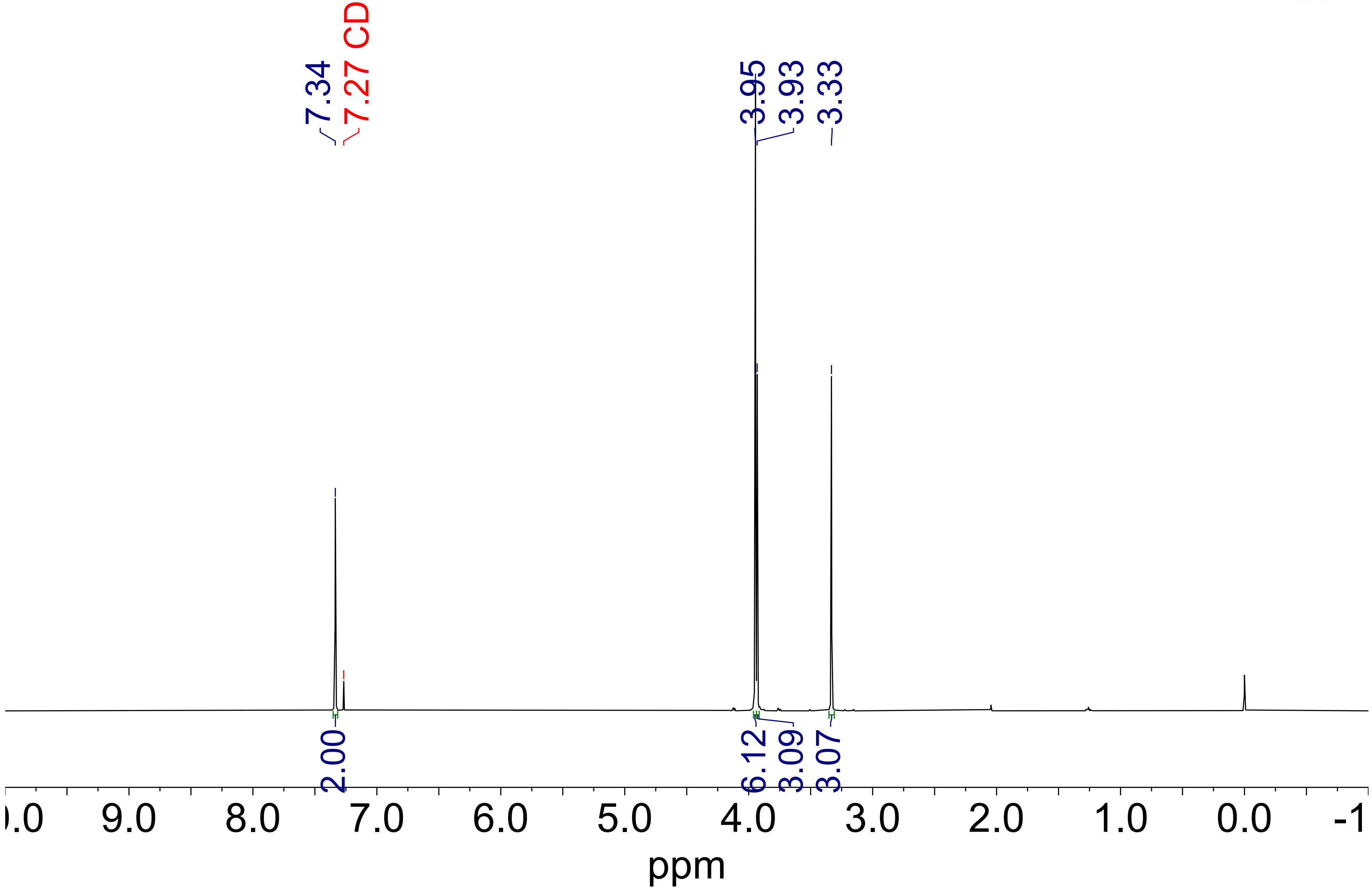

^{13}**C NMR** (100 MHz, $CDCl_3$) of **S–OMs**

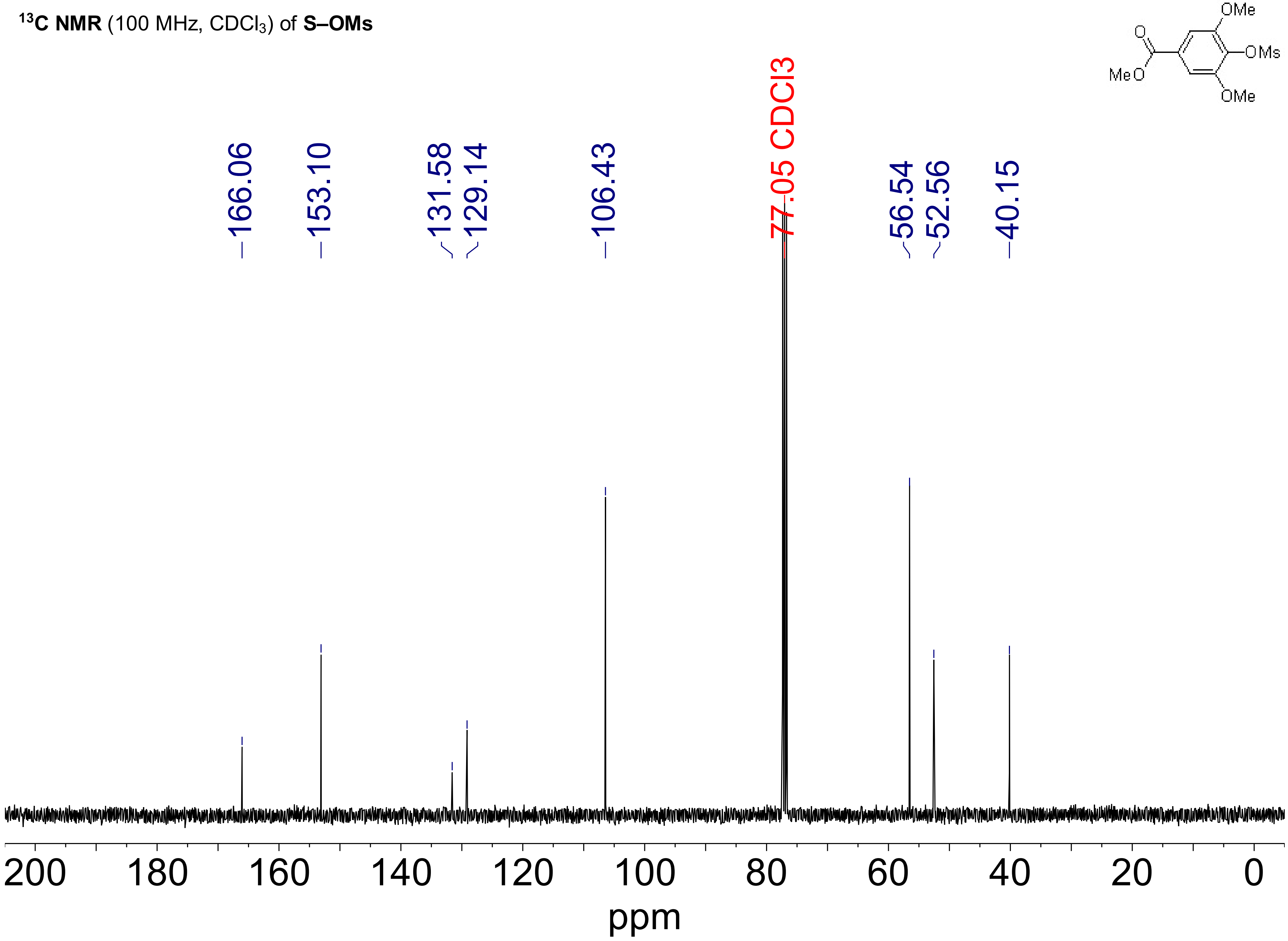

^{1}H NMR (400 MHz, CDCl$_3$) of **H–OTs**

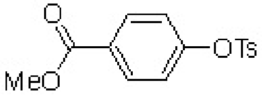

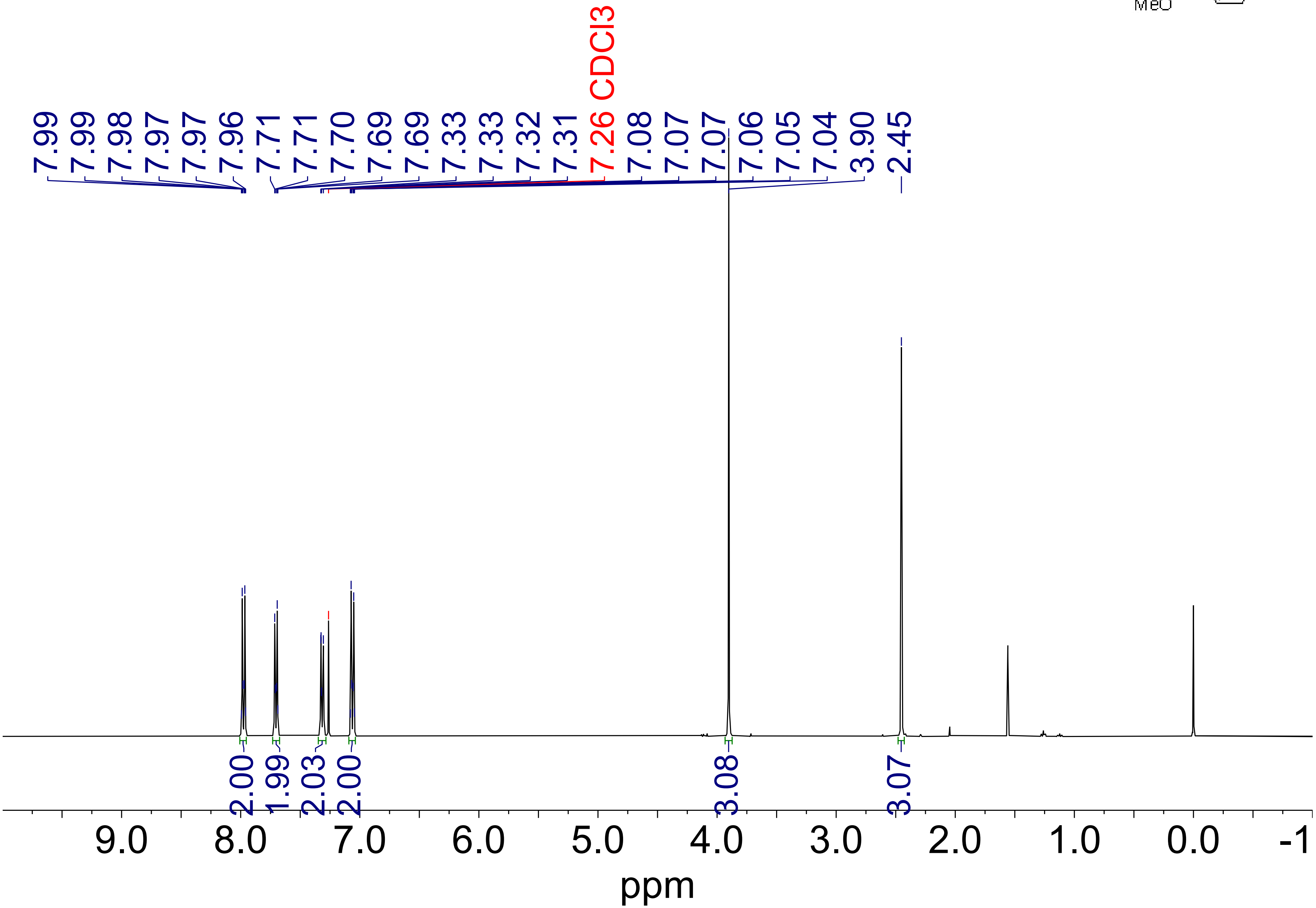

^{13}C NMR (100 MHz, $CDCl_3$) of **H–OTs**

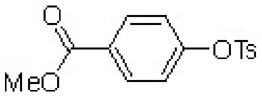

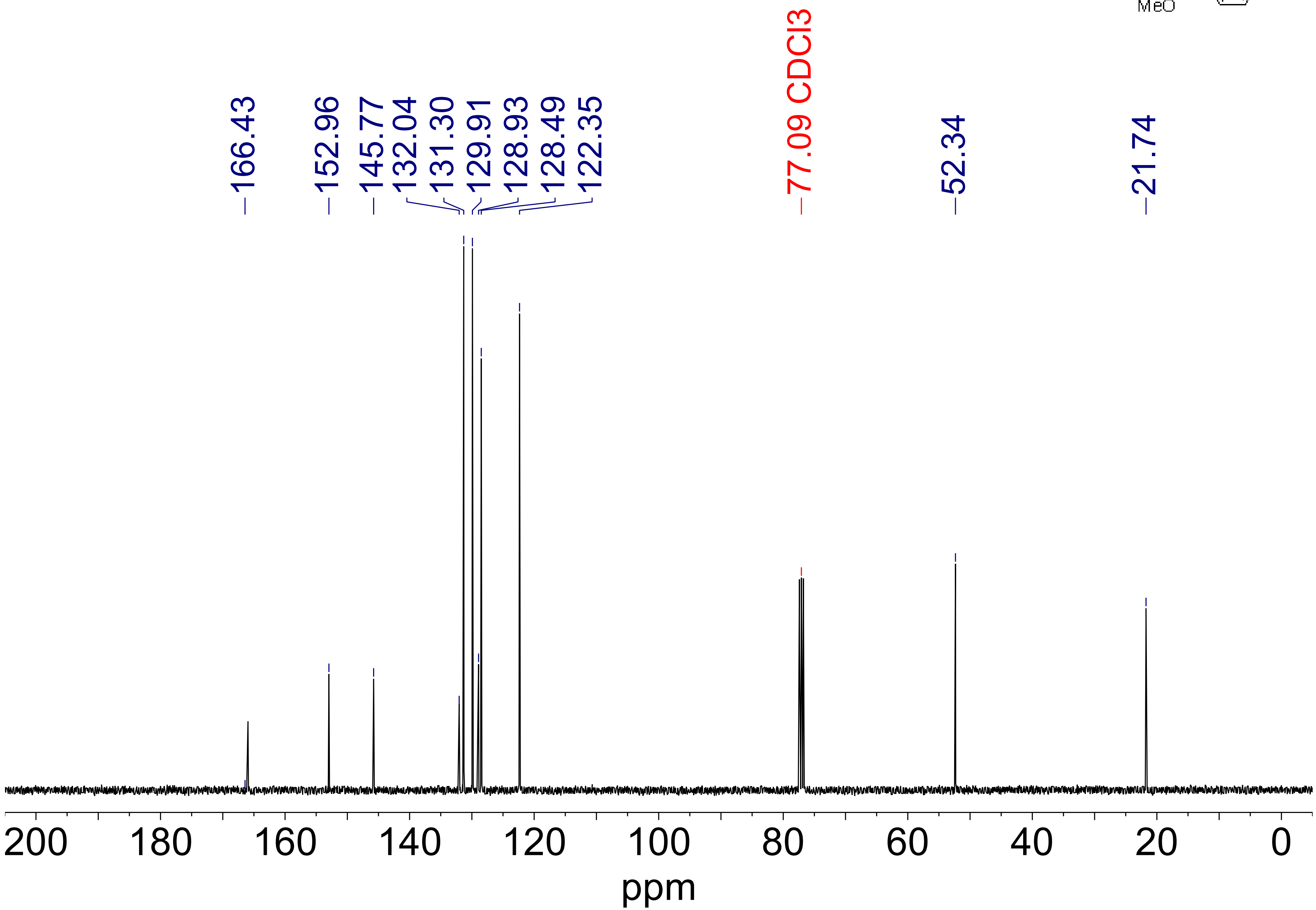

^{1}**H NMR** (400 MHz, $CDCl_3$) of **G–OTs**

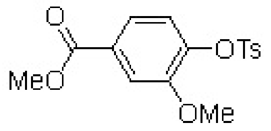

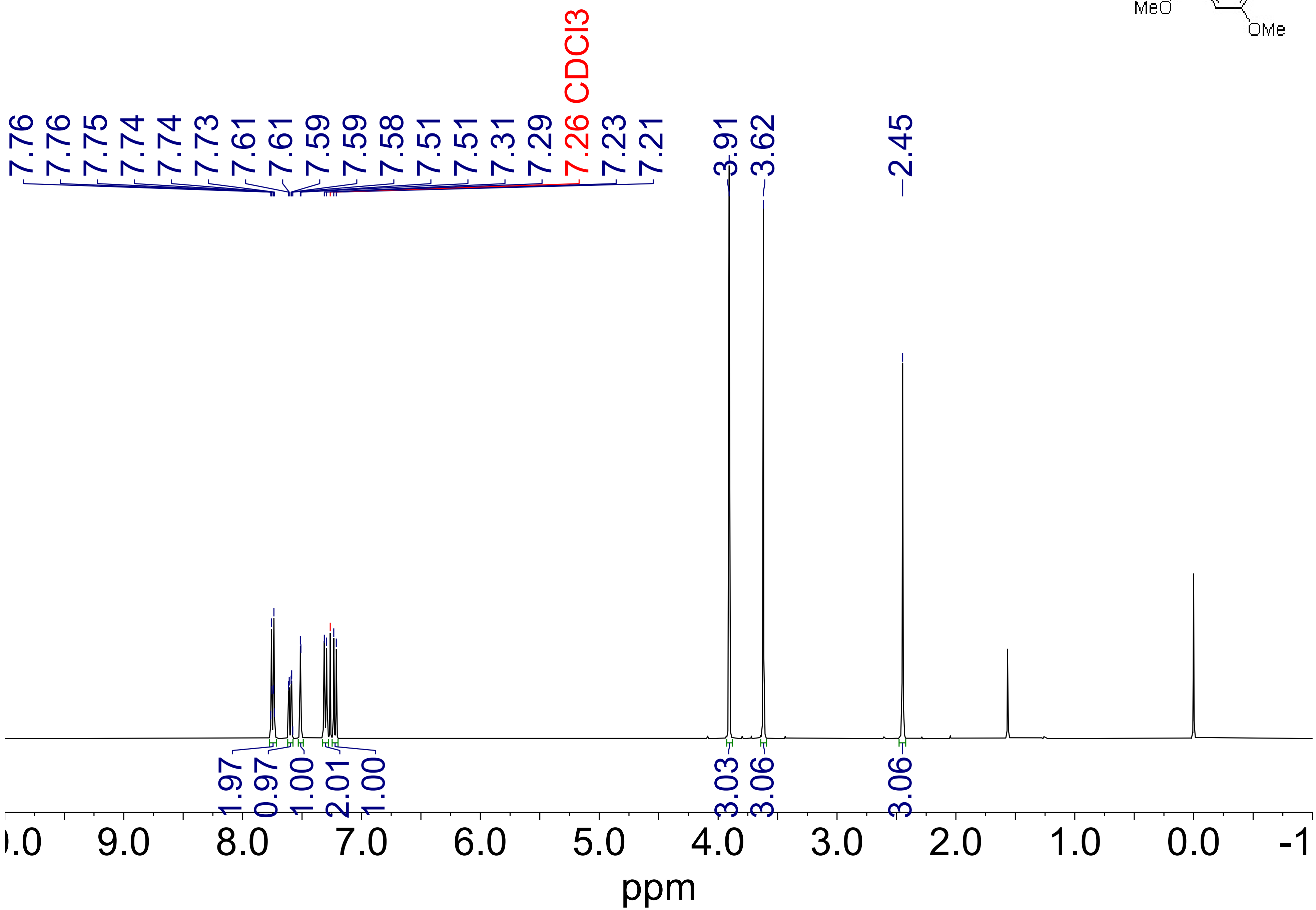

^{13}C NMR (100 MHz, $CDCl_3$) of **G–OTs**

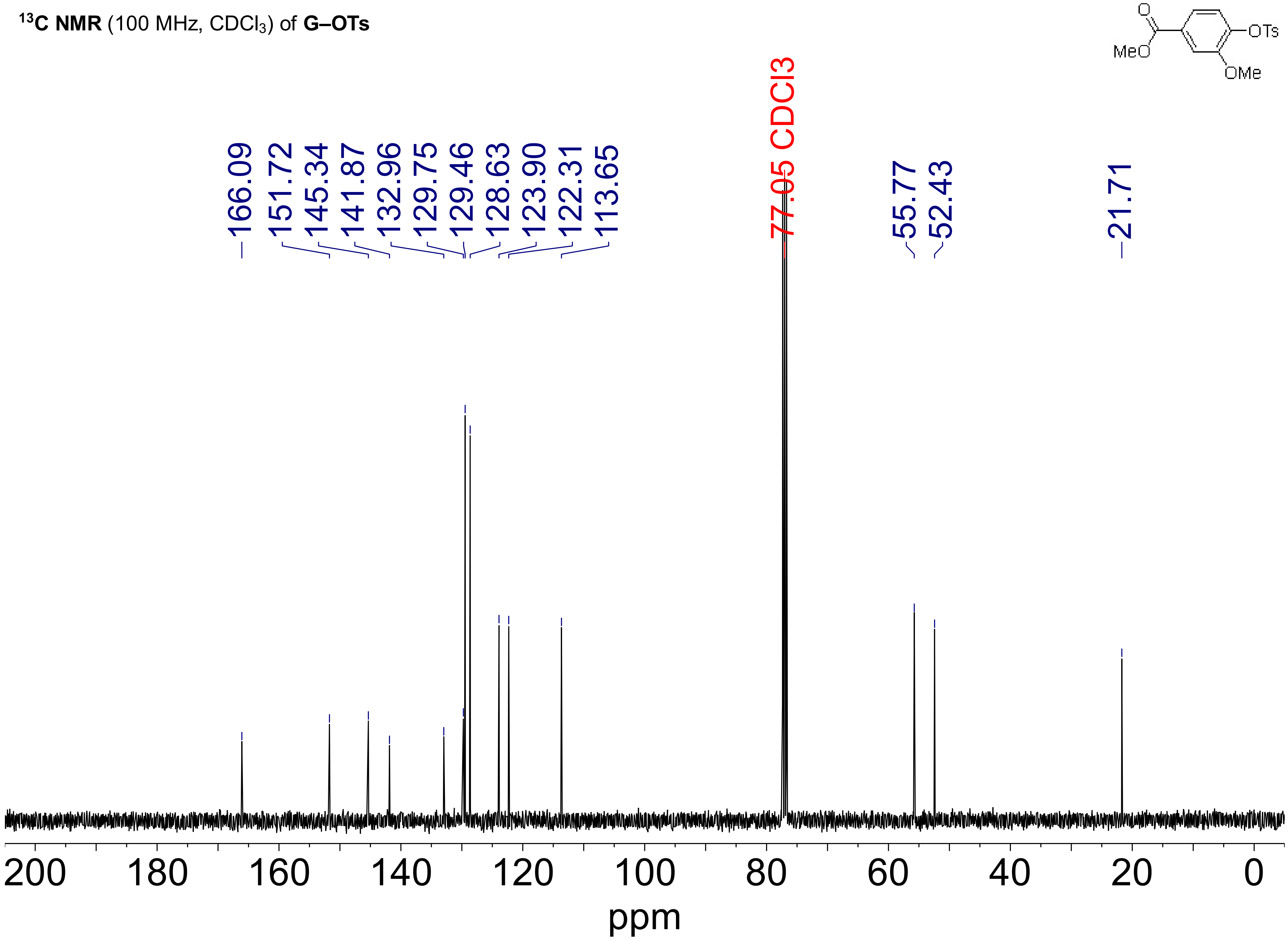

^{1}H NMR (400 MHz, $CDCl_3$) of **S–OTs**

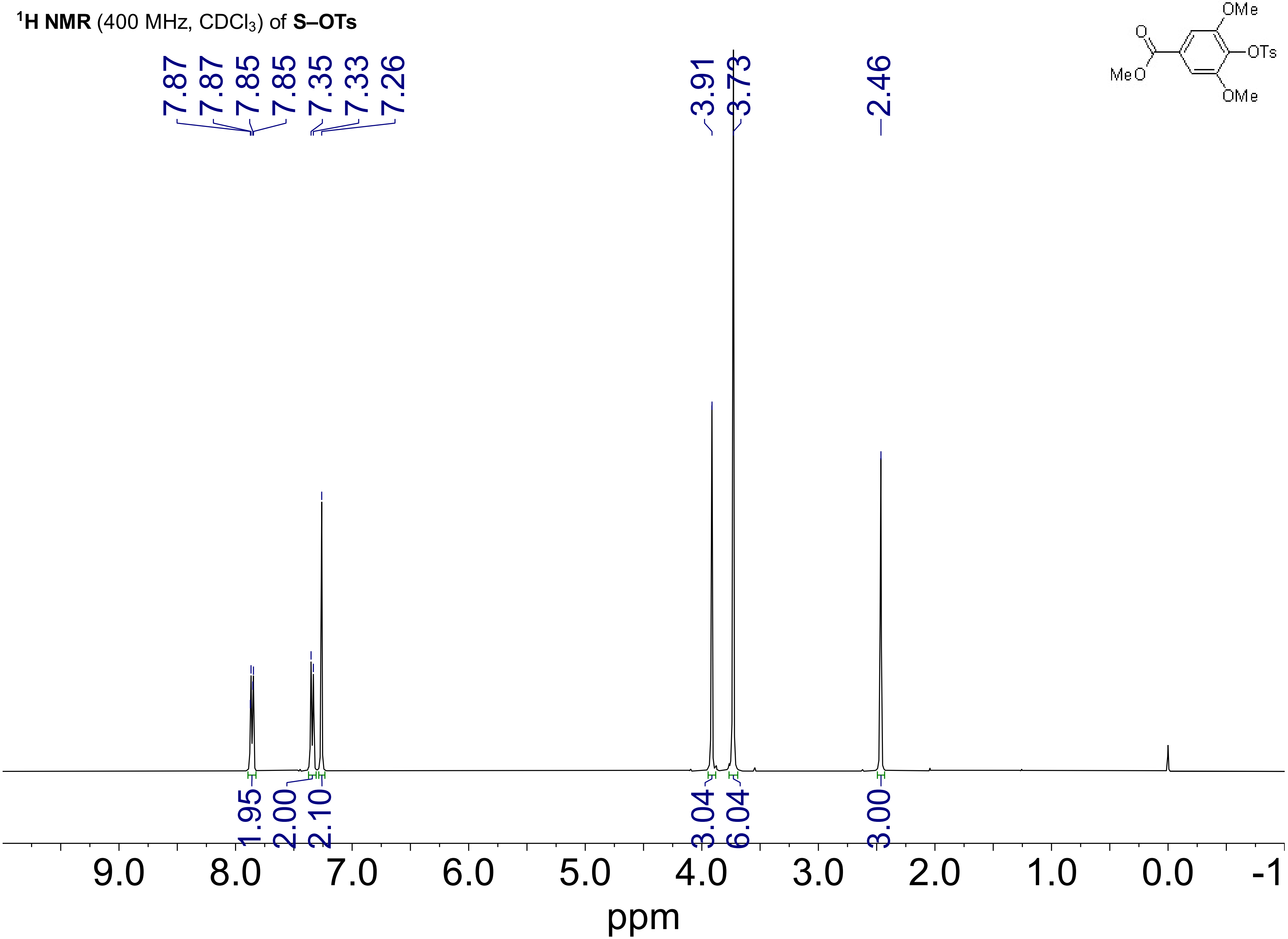

^{13}C NMR (100 MHz, $CDCl_3$) of **S–OTs**

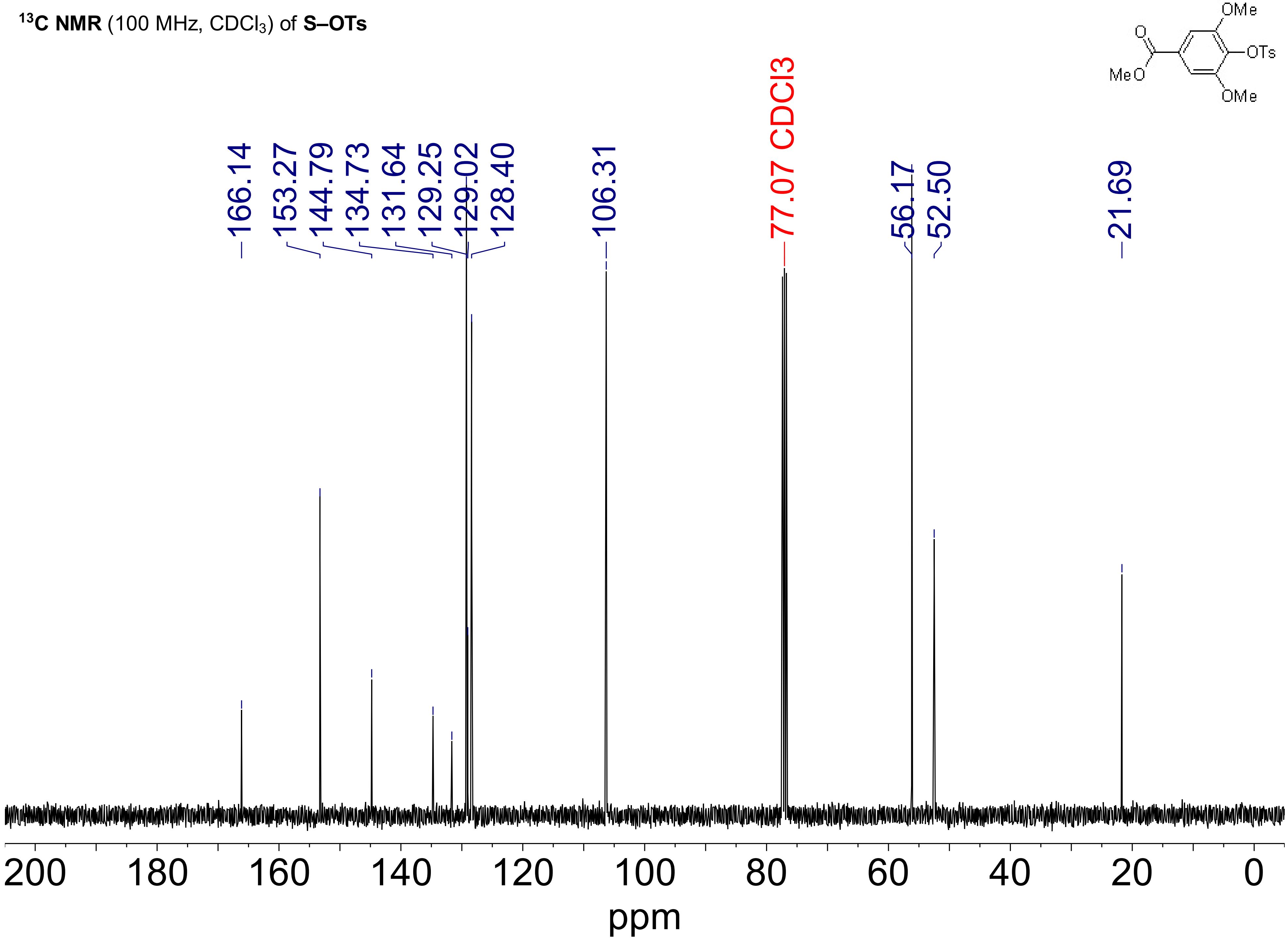

^{1}H NMR (400 MHz, $CDCl_3$) of **G–OTf**

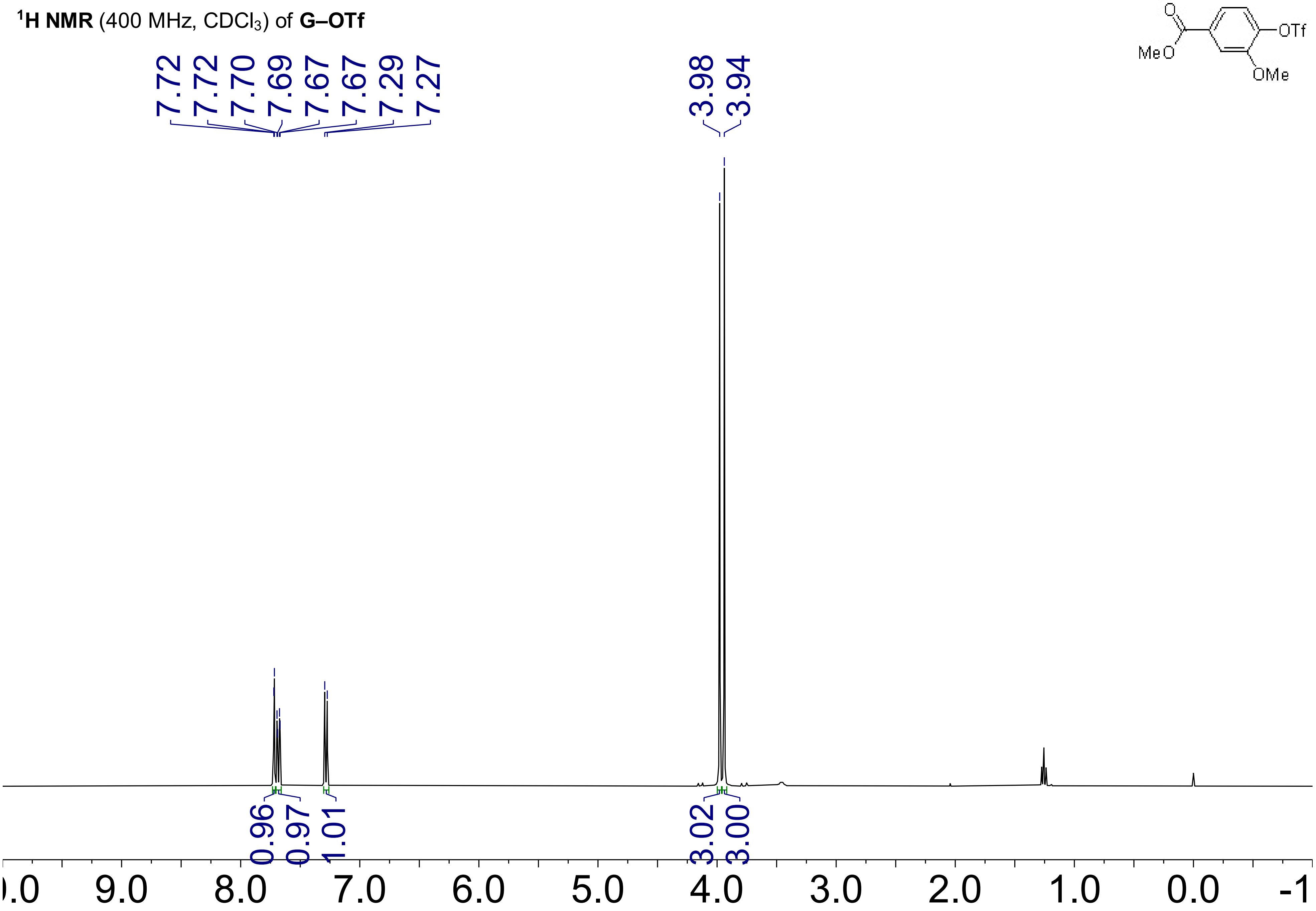

^{13}C NMR (100 MHz, $CDCl_3$) of **G–OTf**

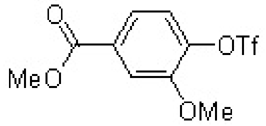

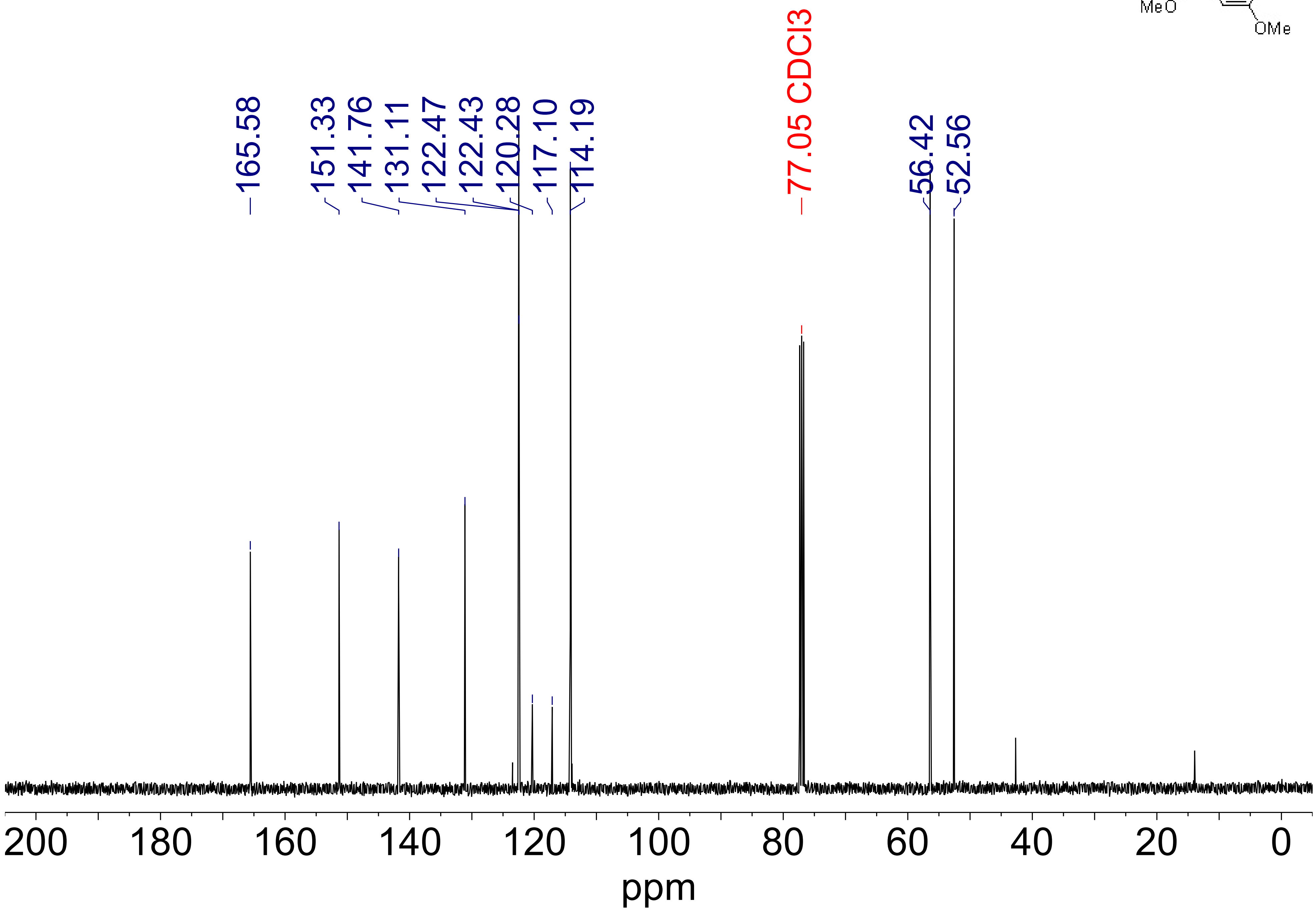

^{1}H NMR (400 MHz, $CDCl_3$) of **S–OTf**

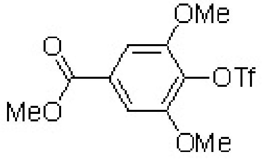

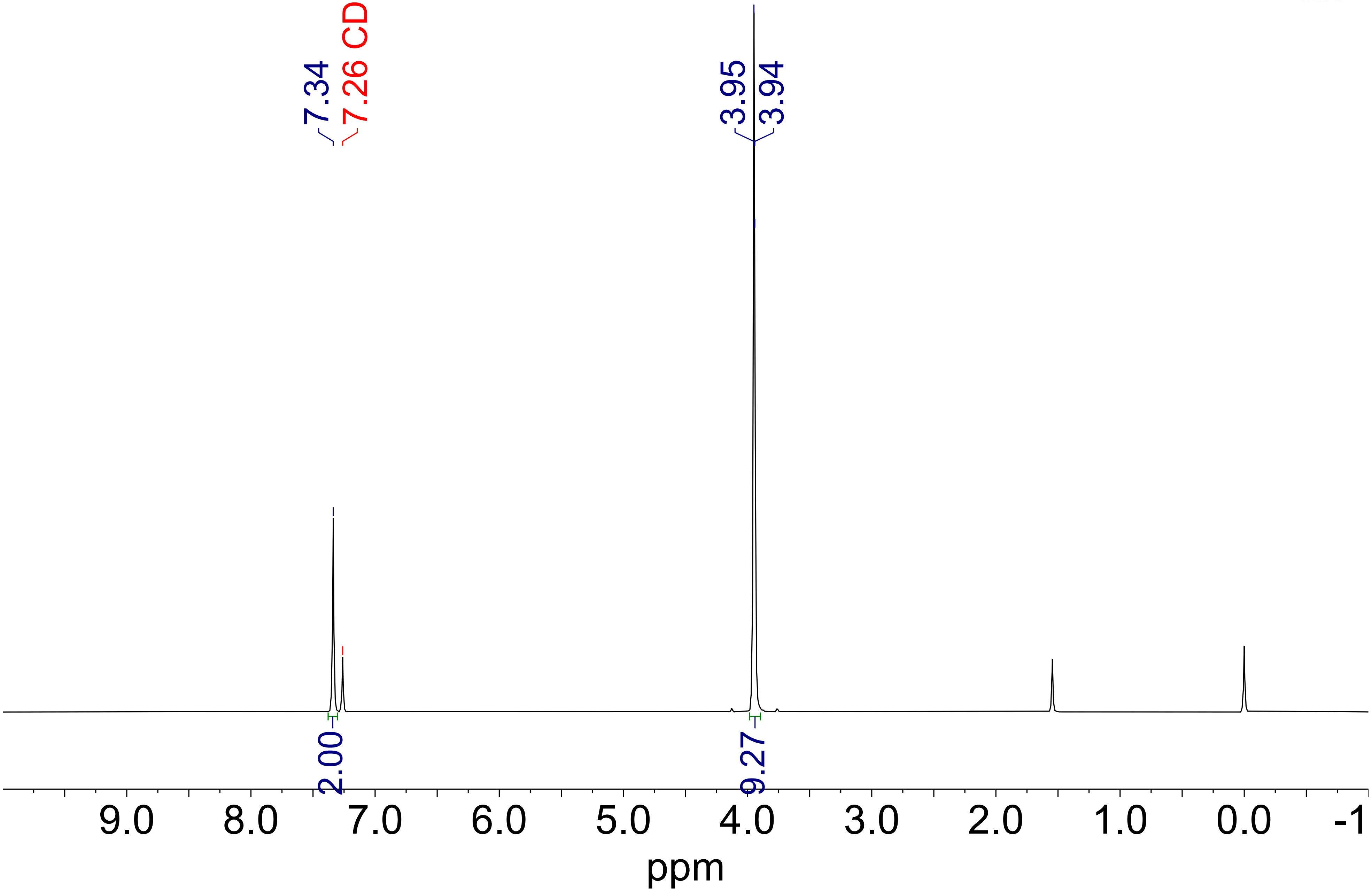

^{13}C NMR (100 MHz, $CDCl_3$) of **S–OTf**

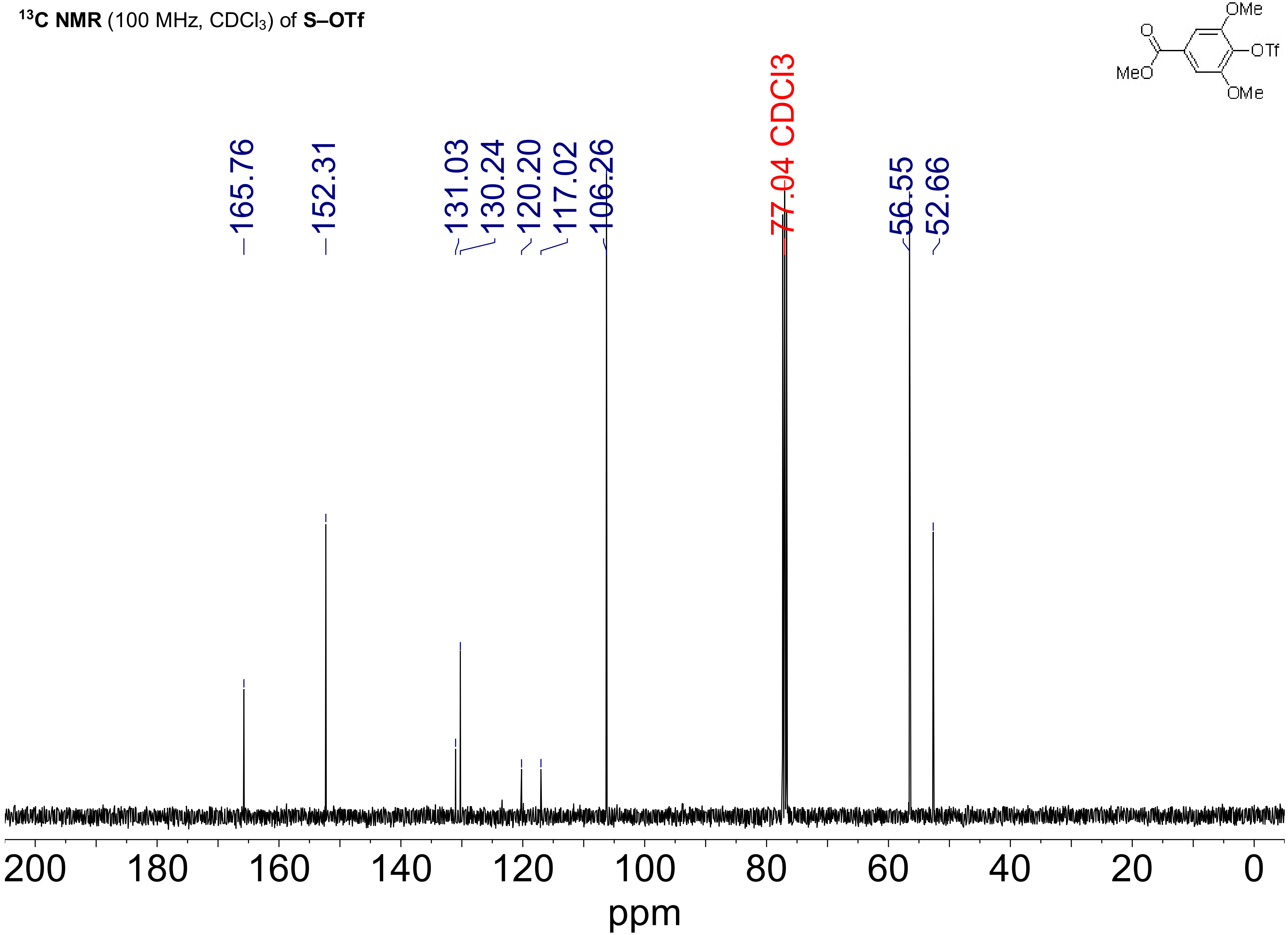

^{1}H NMR (400 MHz, $CDCl_3$) of **H–H**

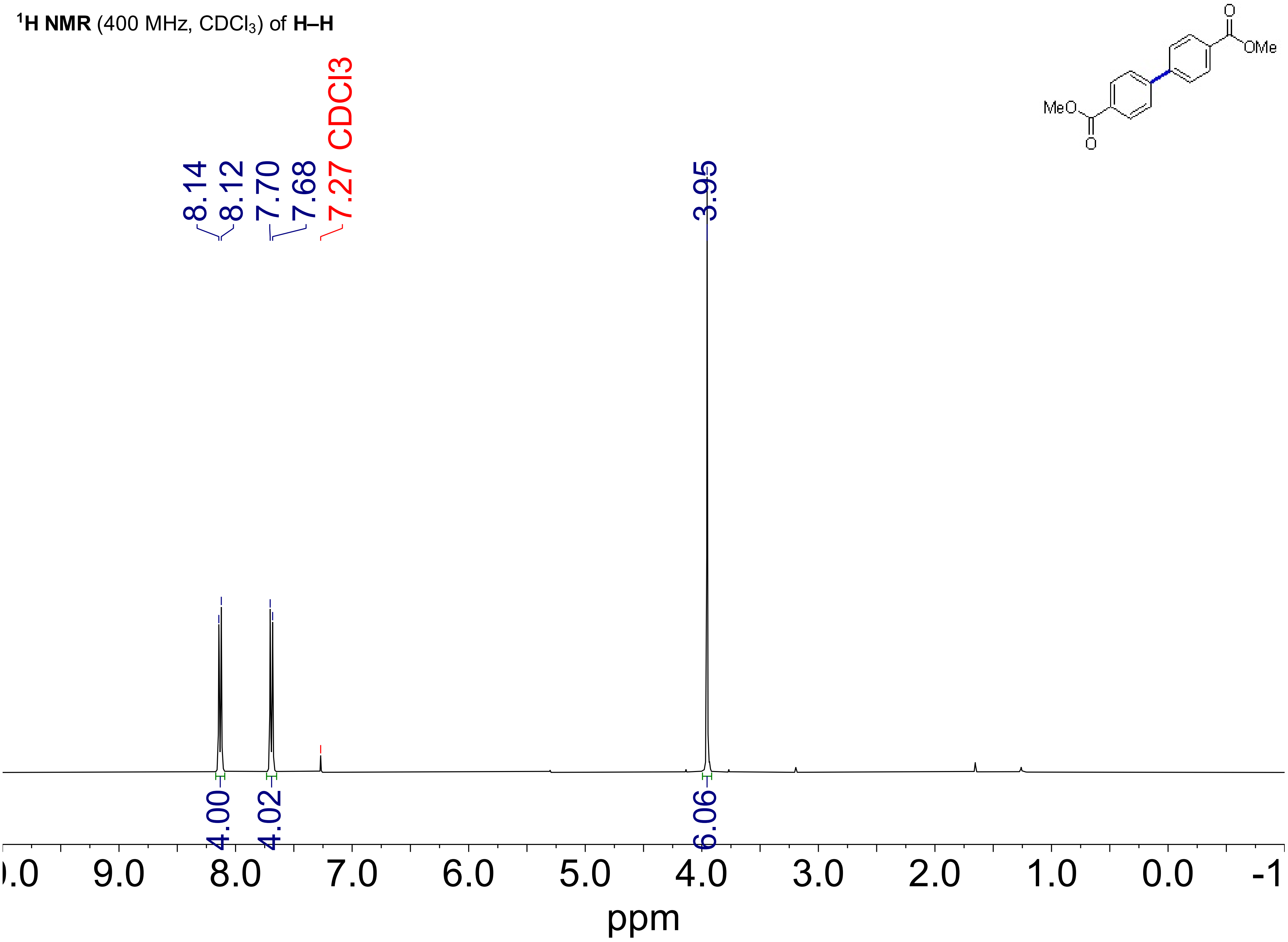

^{13}**C NMR** (126 MHz, $CDCl_3$) of **H–H**

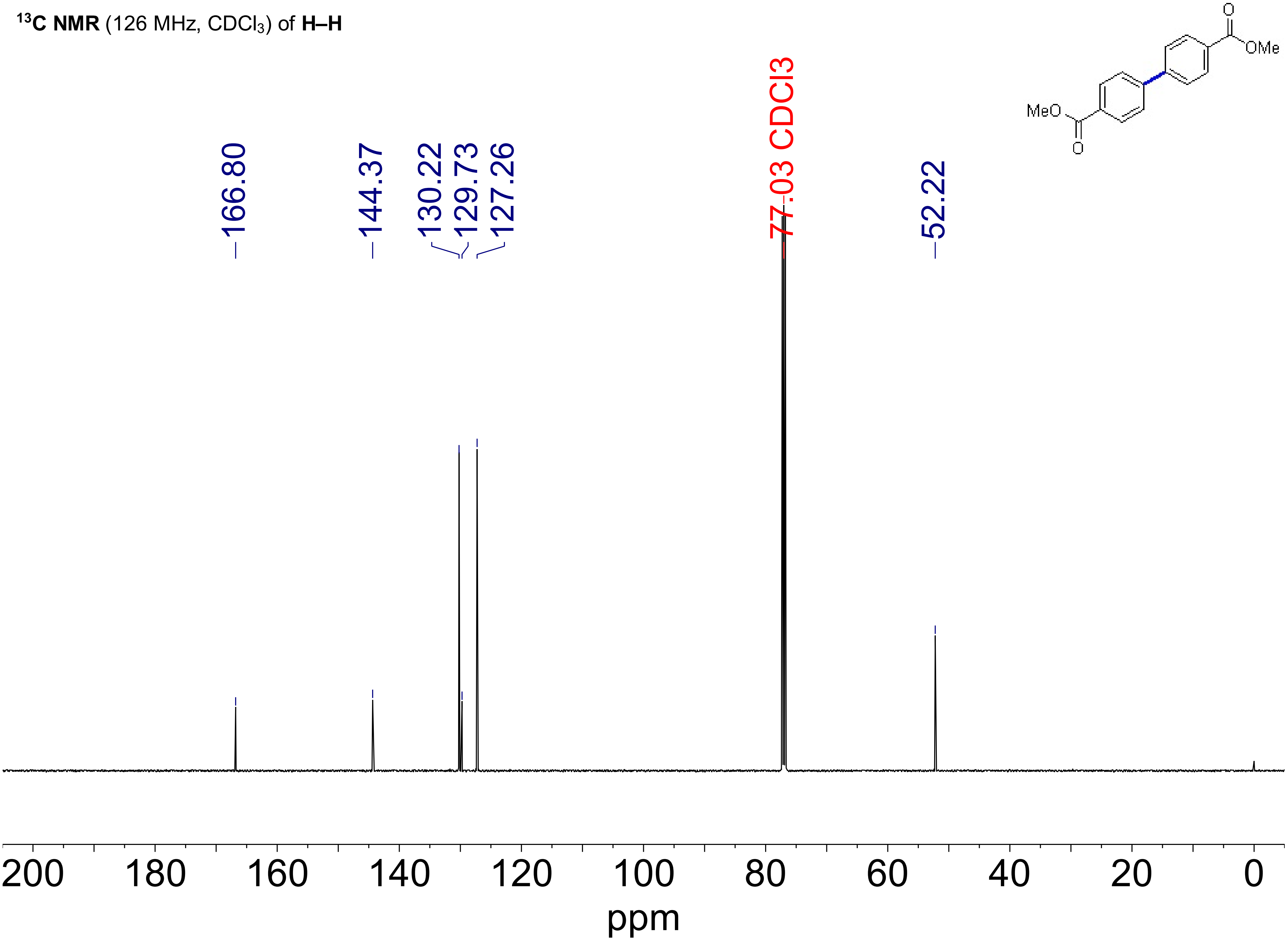

^{1}H NMR (400 MHz, $CDCl_3$) of **G–G**

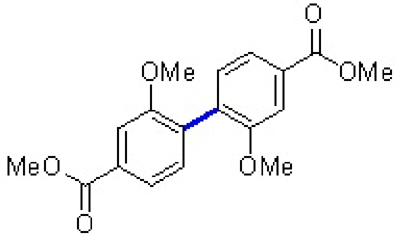

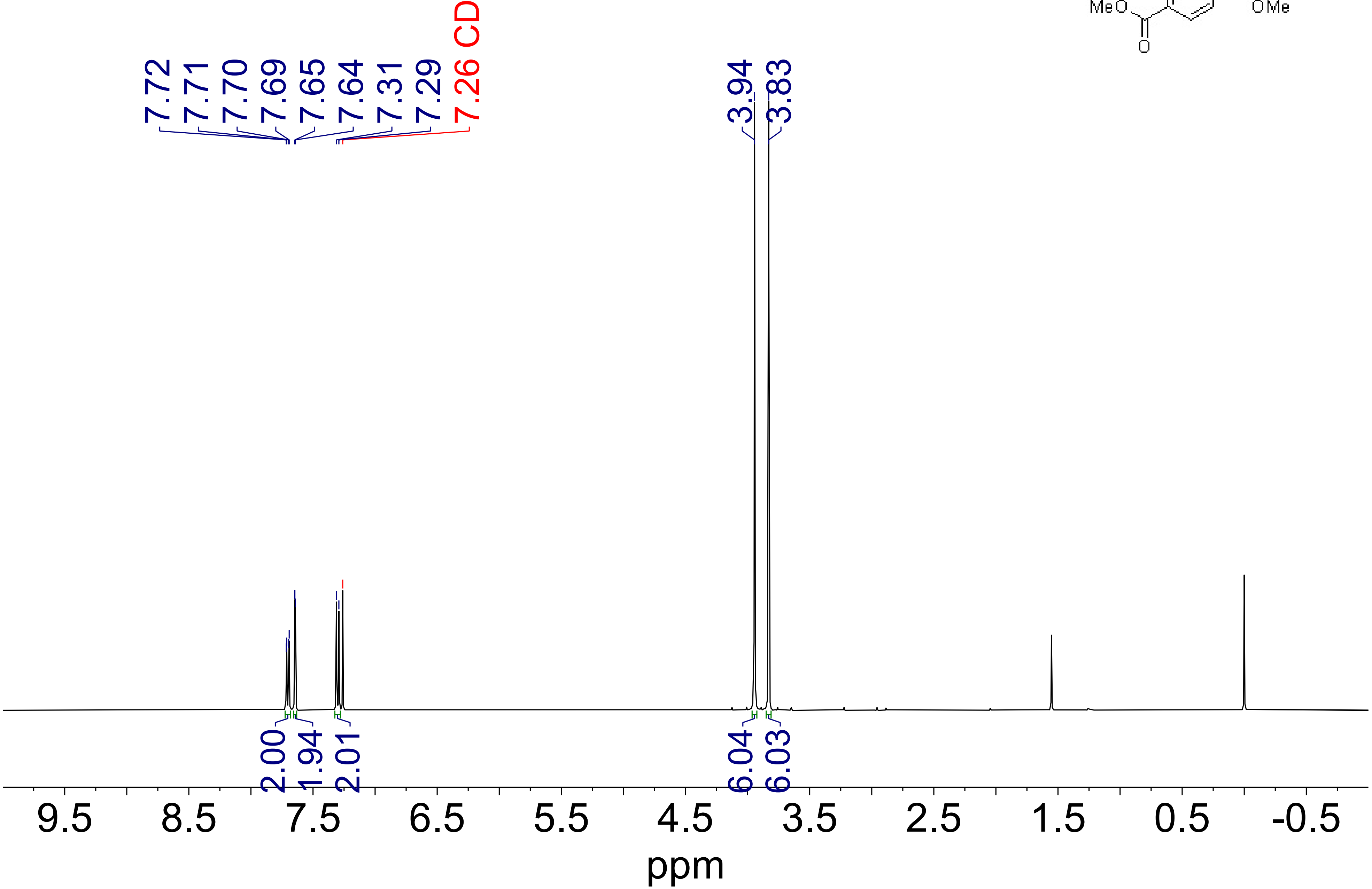

^{13}**C NMR** (126 MHz, $CDCl_3$) of **G–G**

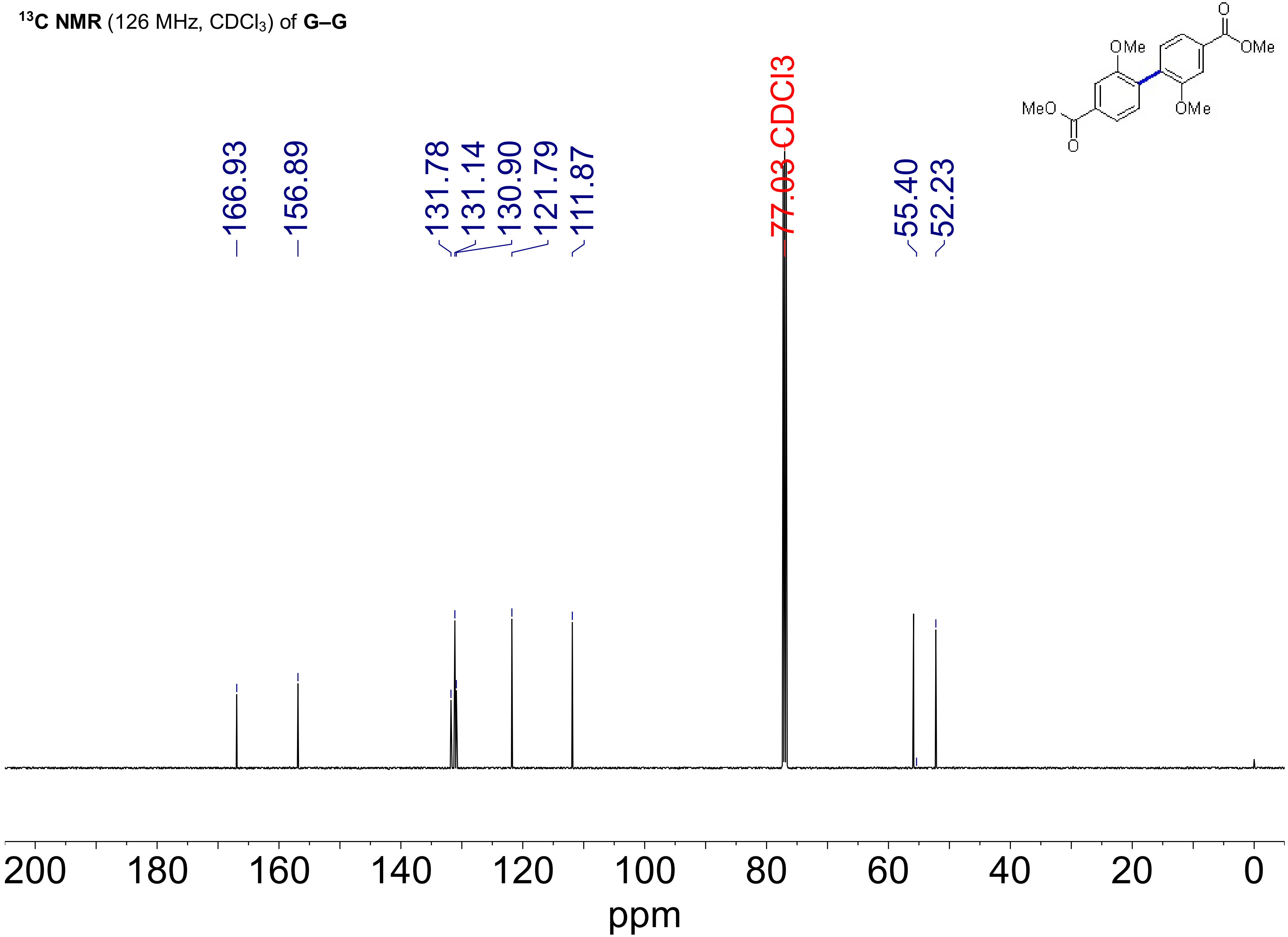

¹H NMR (400 MHz, $CDCl_3$) of **S–S**

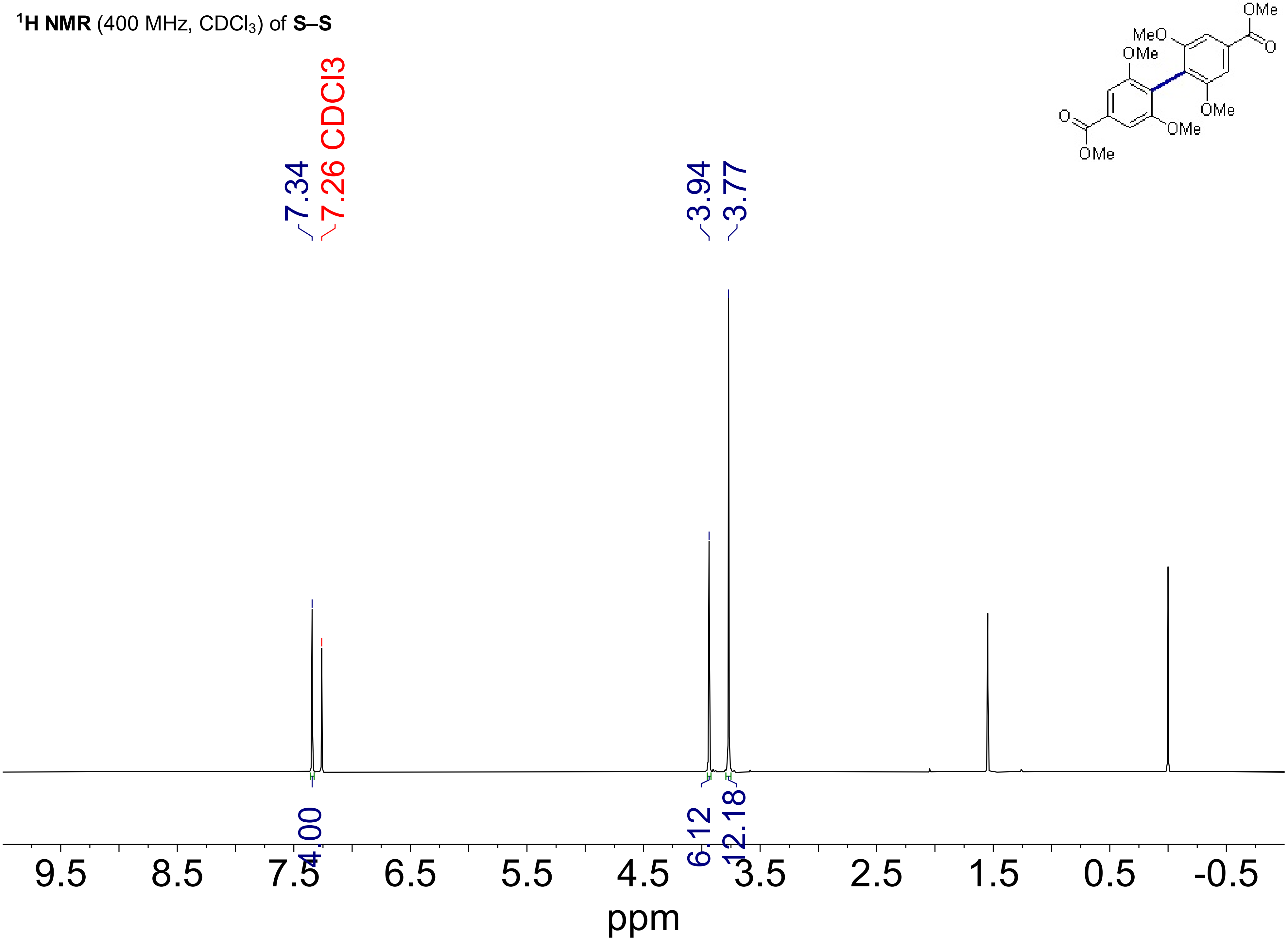

^{13}**C NMR** (151 MHz, $CDCl_3$) of **S–S**

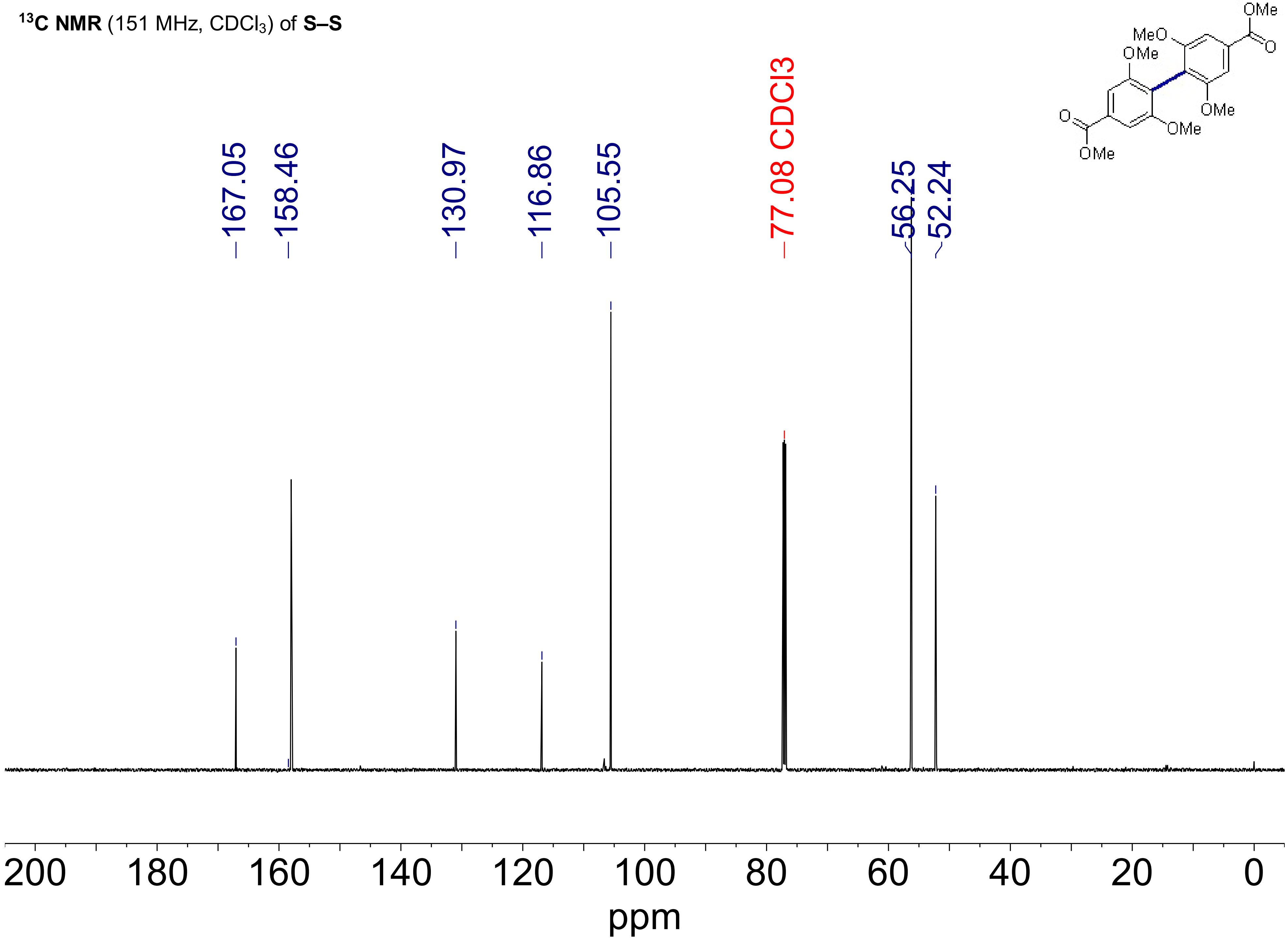

^{1}H NMR (400 MHz, $CDCl_3$) of **H–G**

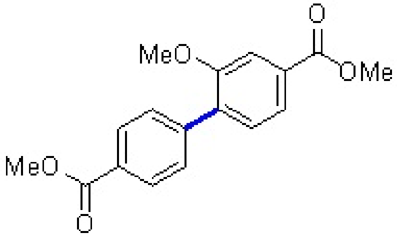

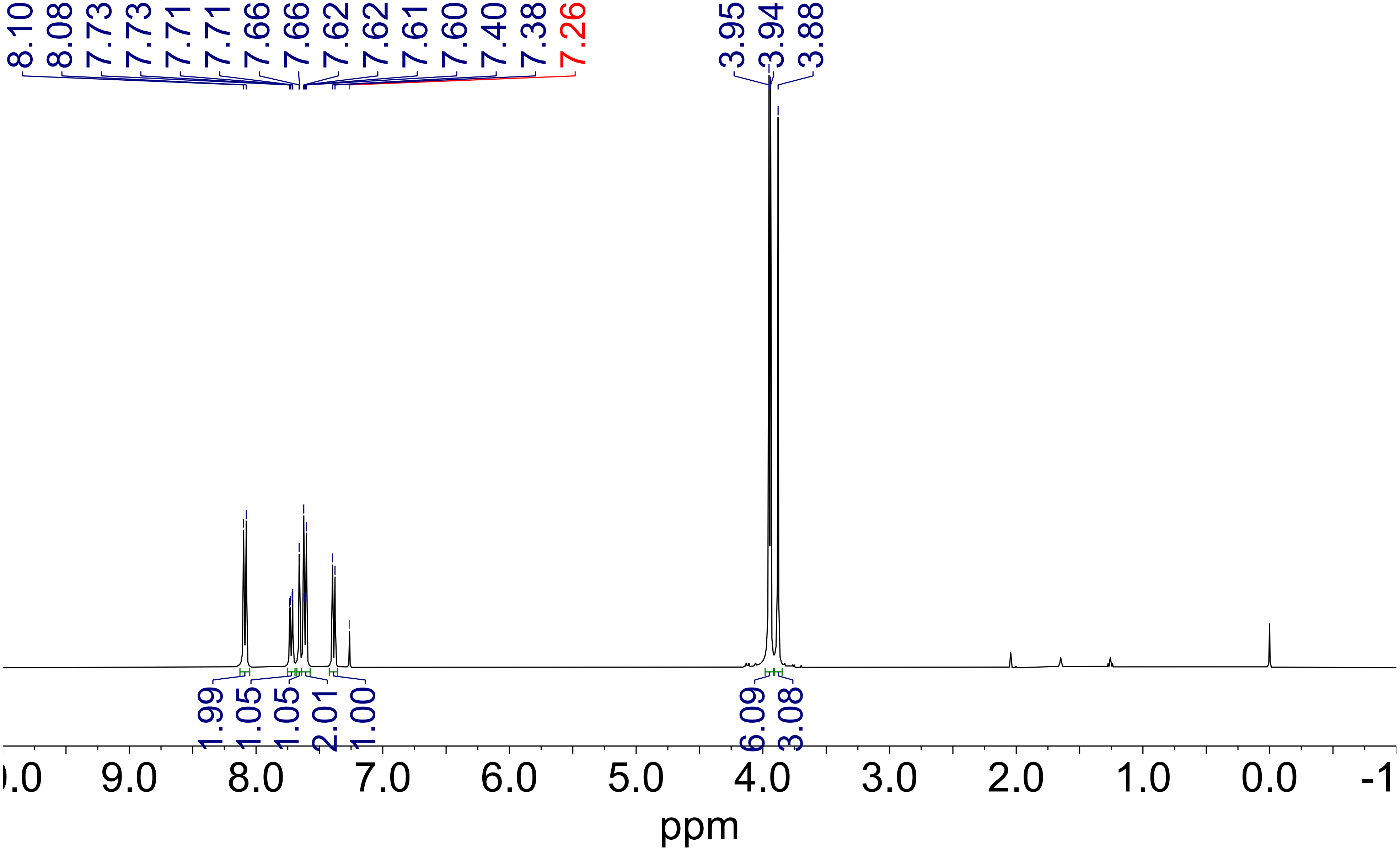

^{13}C NMR (100 MHz, $CDCl_3$) of **H–G**

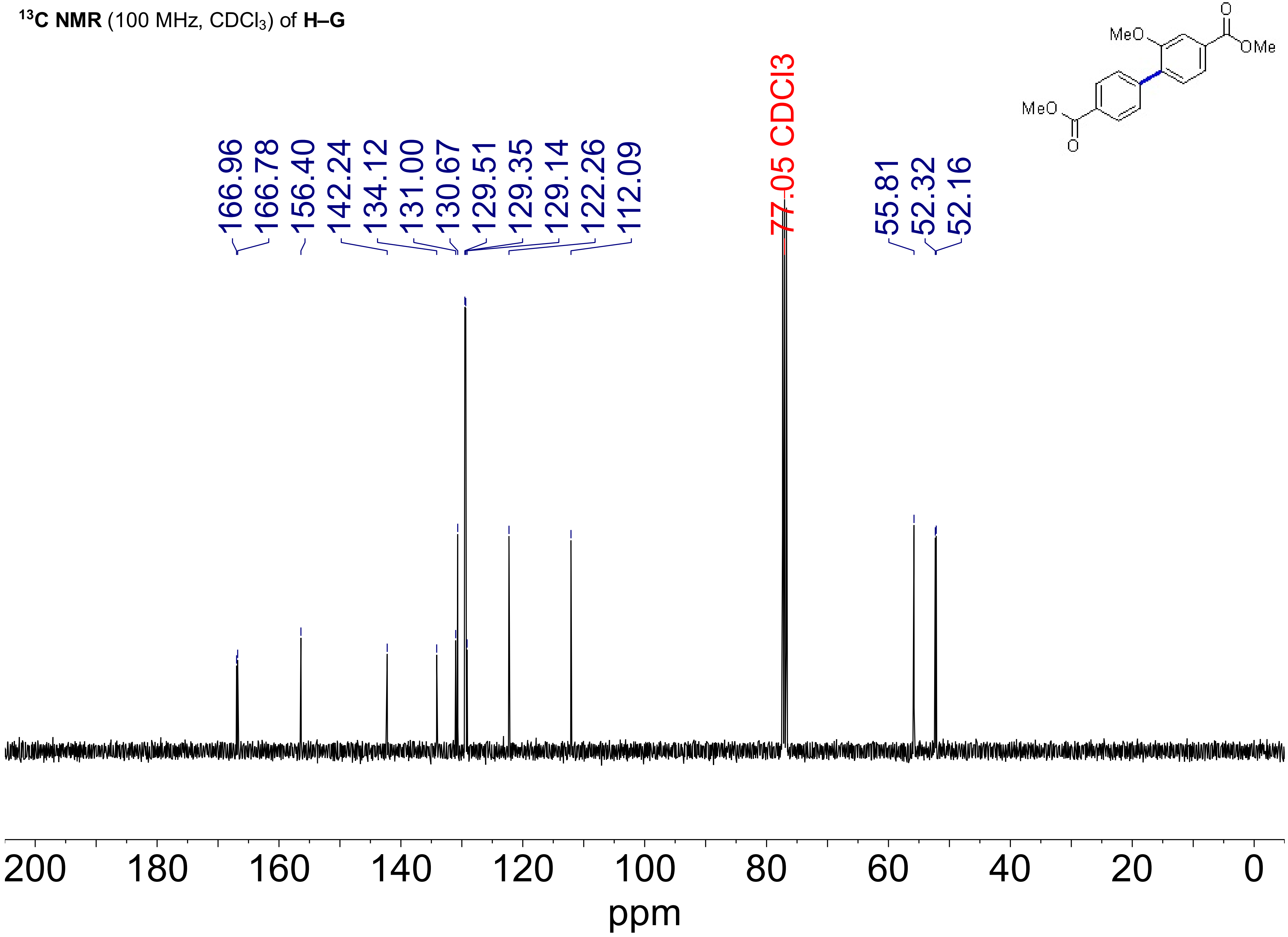

^{1}H NMR (400 MHz, $CDCl_3$) of **H–S**

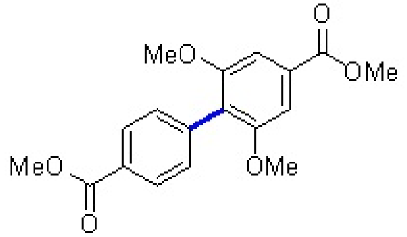

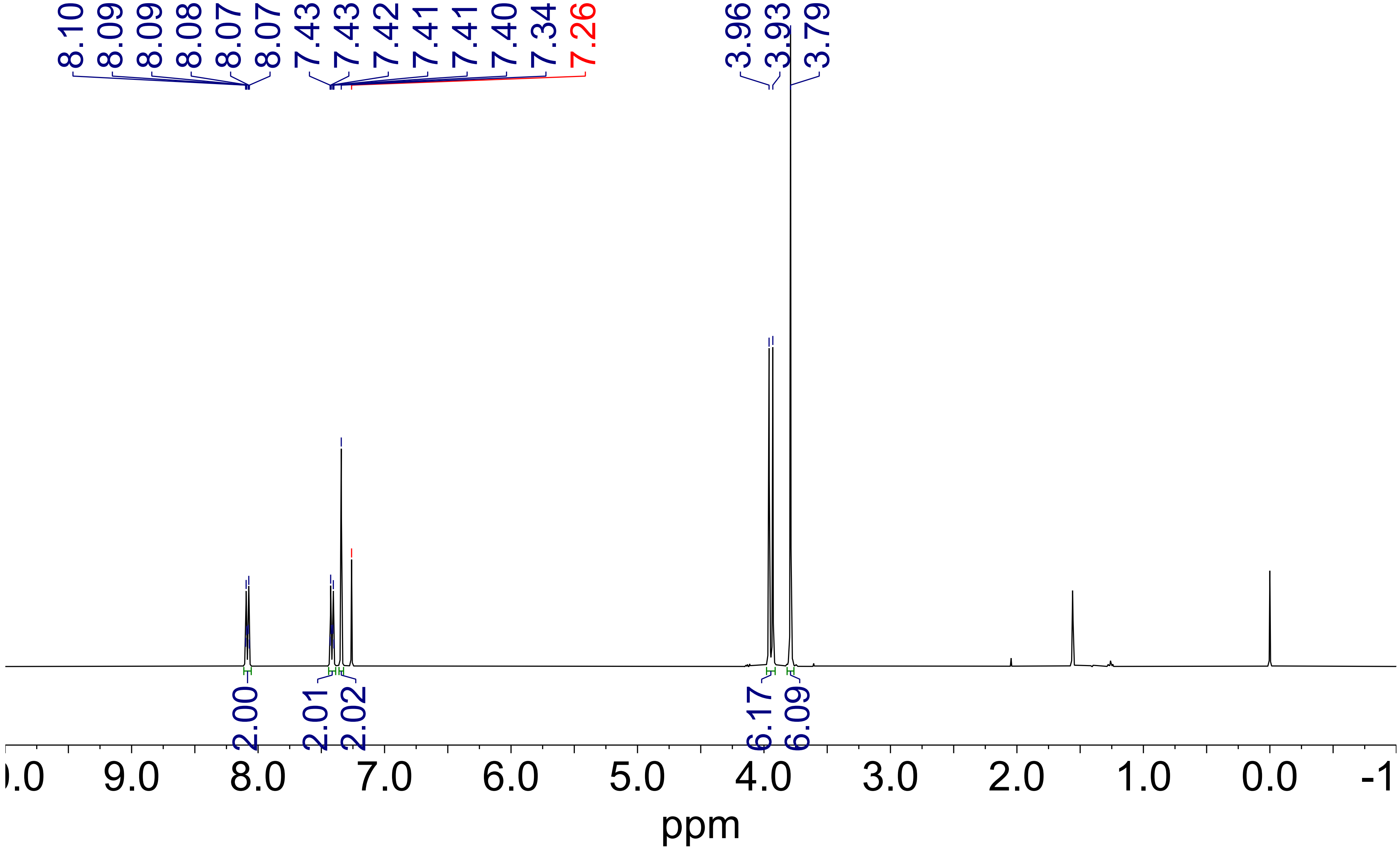

^{13}C NMR (151 MHz, $CDCl_3$) of **H–S**

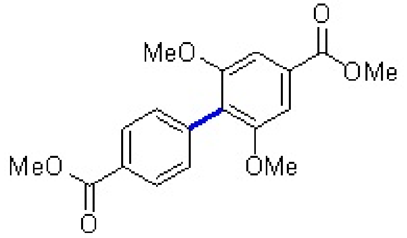

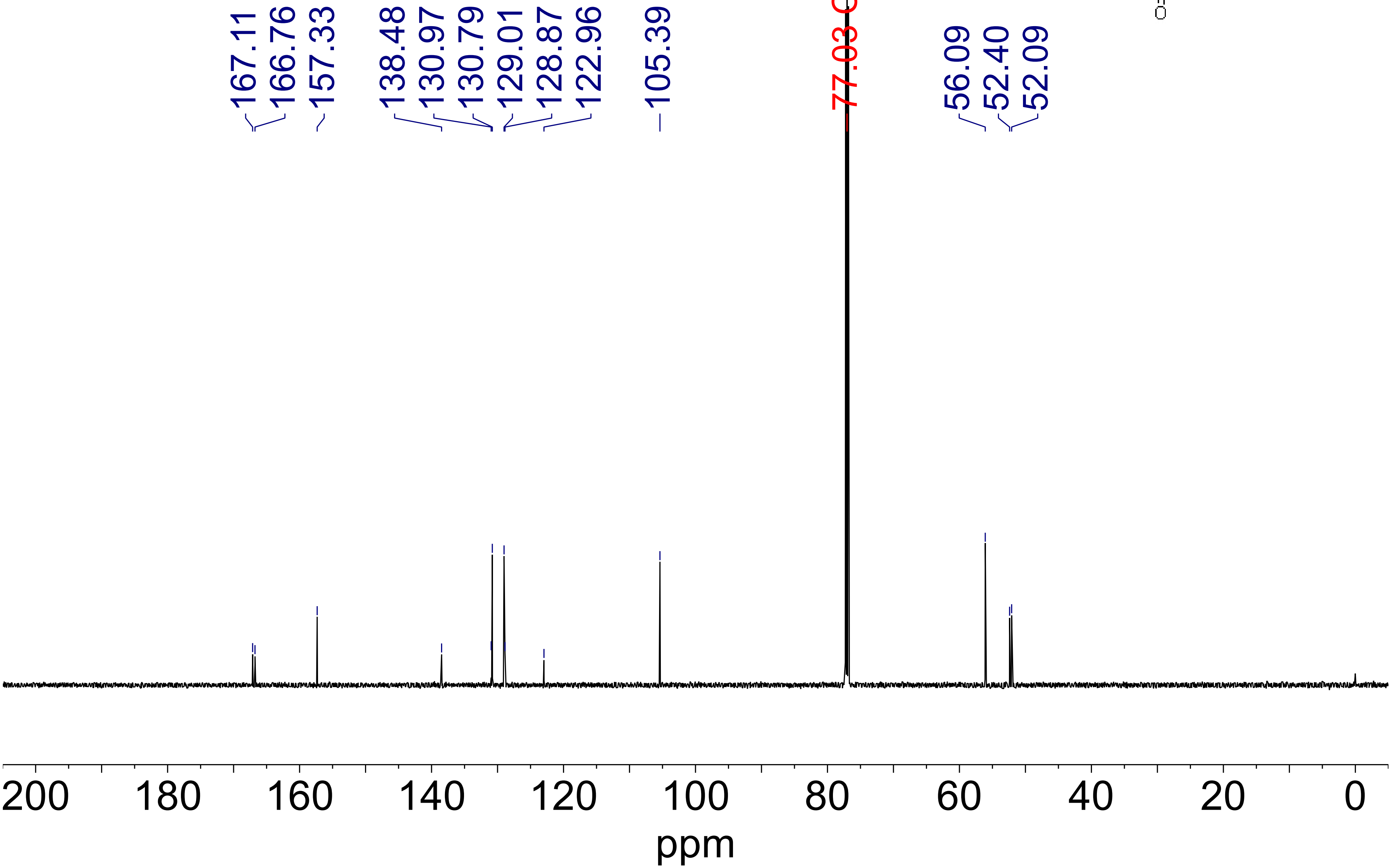

^{1}H NMR (400 MHz, $CDCl_3$) of **G–S**

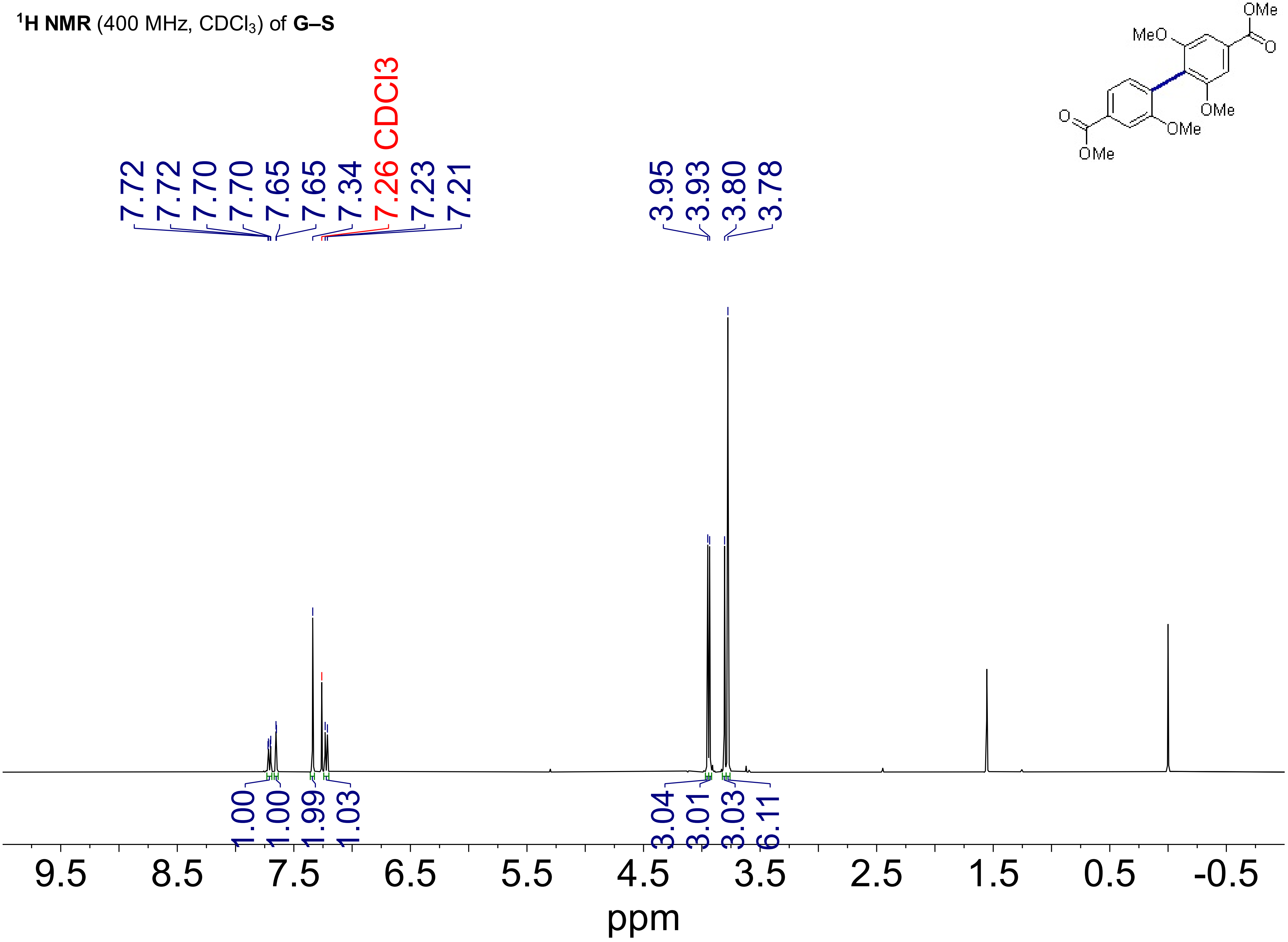

^{13}**C NMR** (151 MHz, $CDCl_3$) of **G–S**

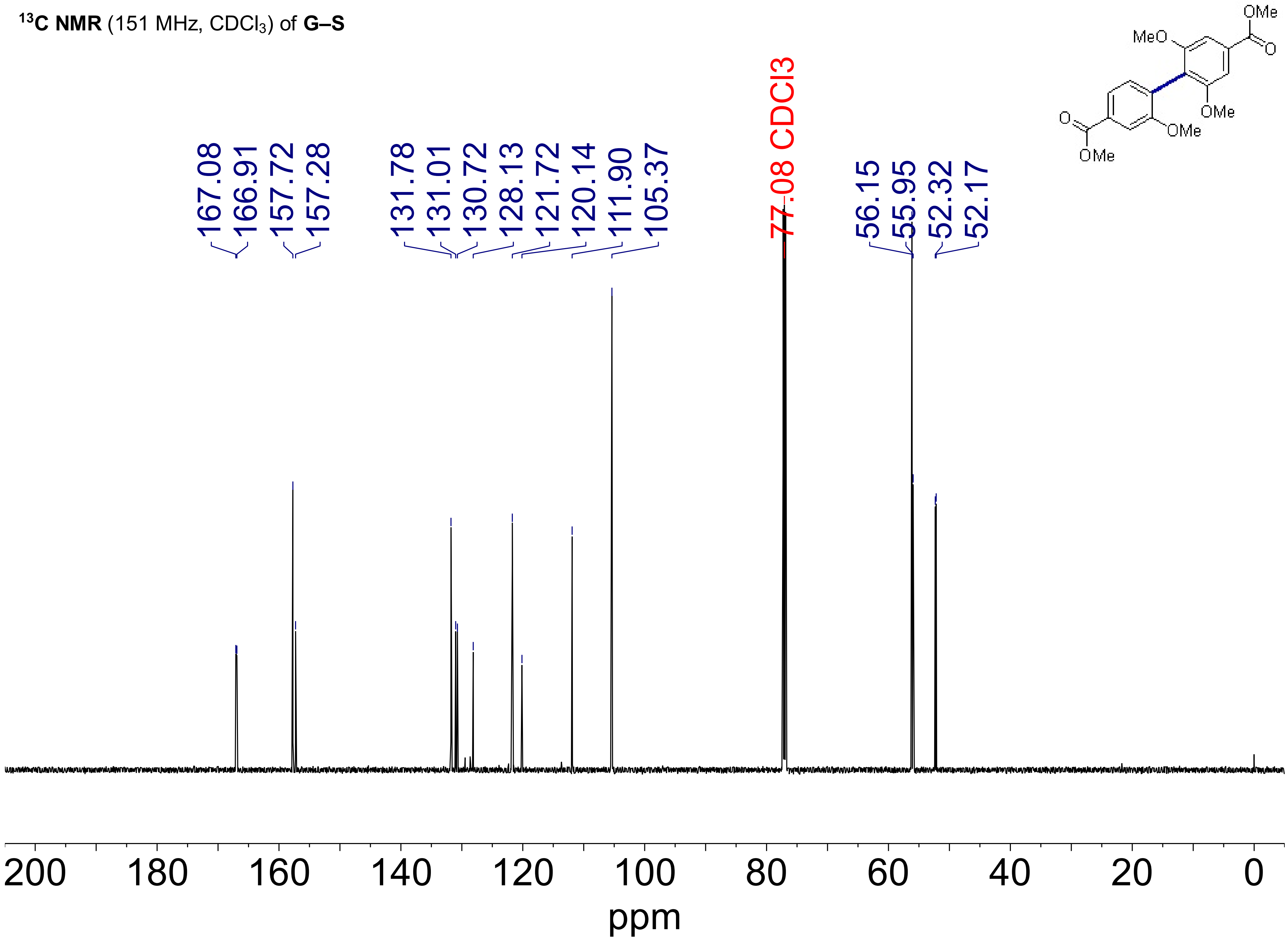

^{1}H NMR (400 MHz, $CDCl_3$) of **Bis(2-ethylhexyl) phthalate (DEHP)**

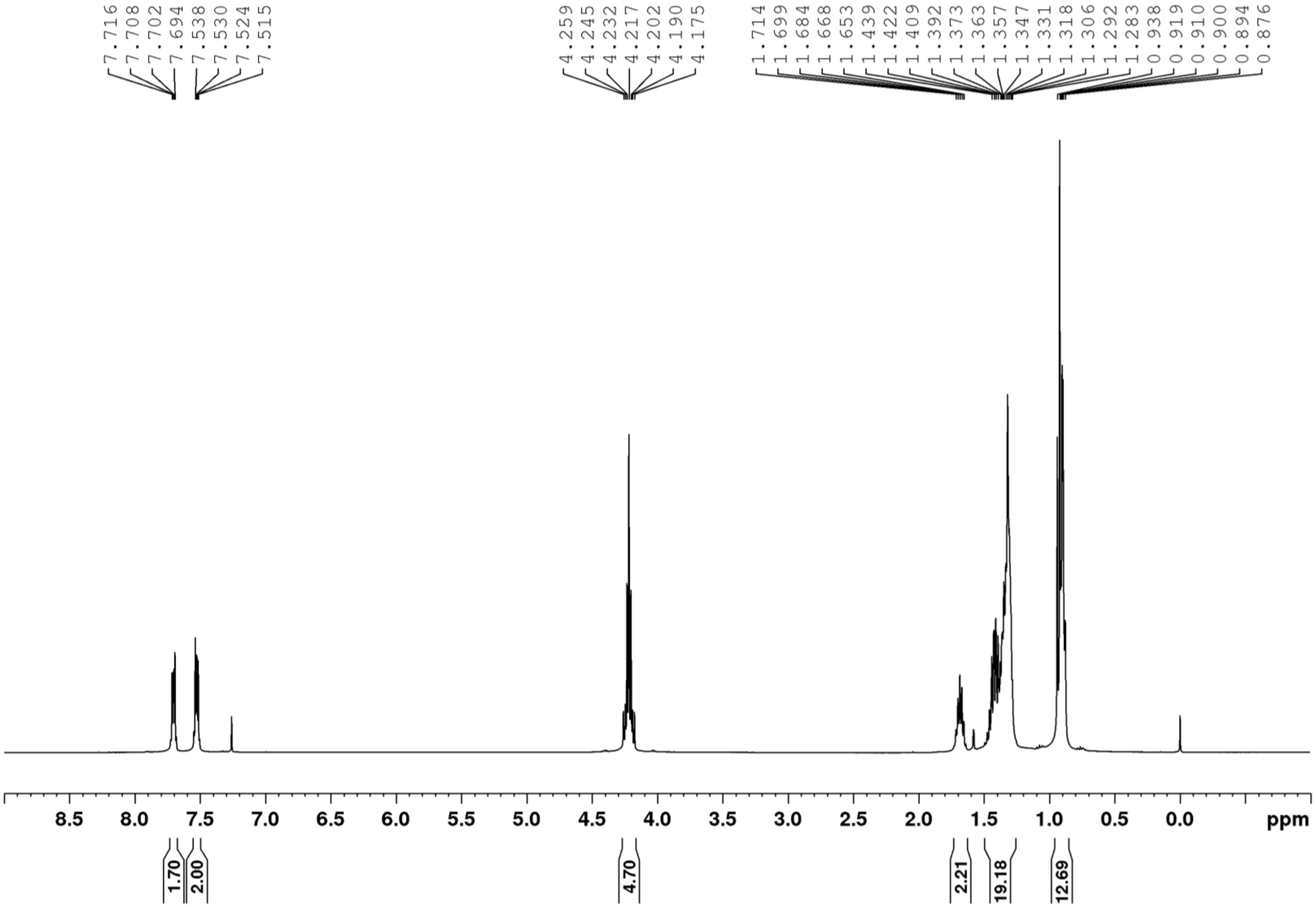

^{13}C NMR (101 MHz, CDCl$_3$) of **Bis(2-ethylhexyl) phthalate (DEHP)**

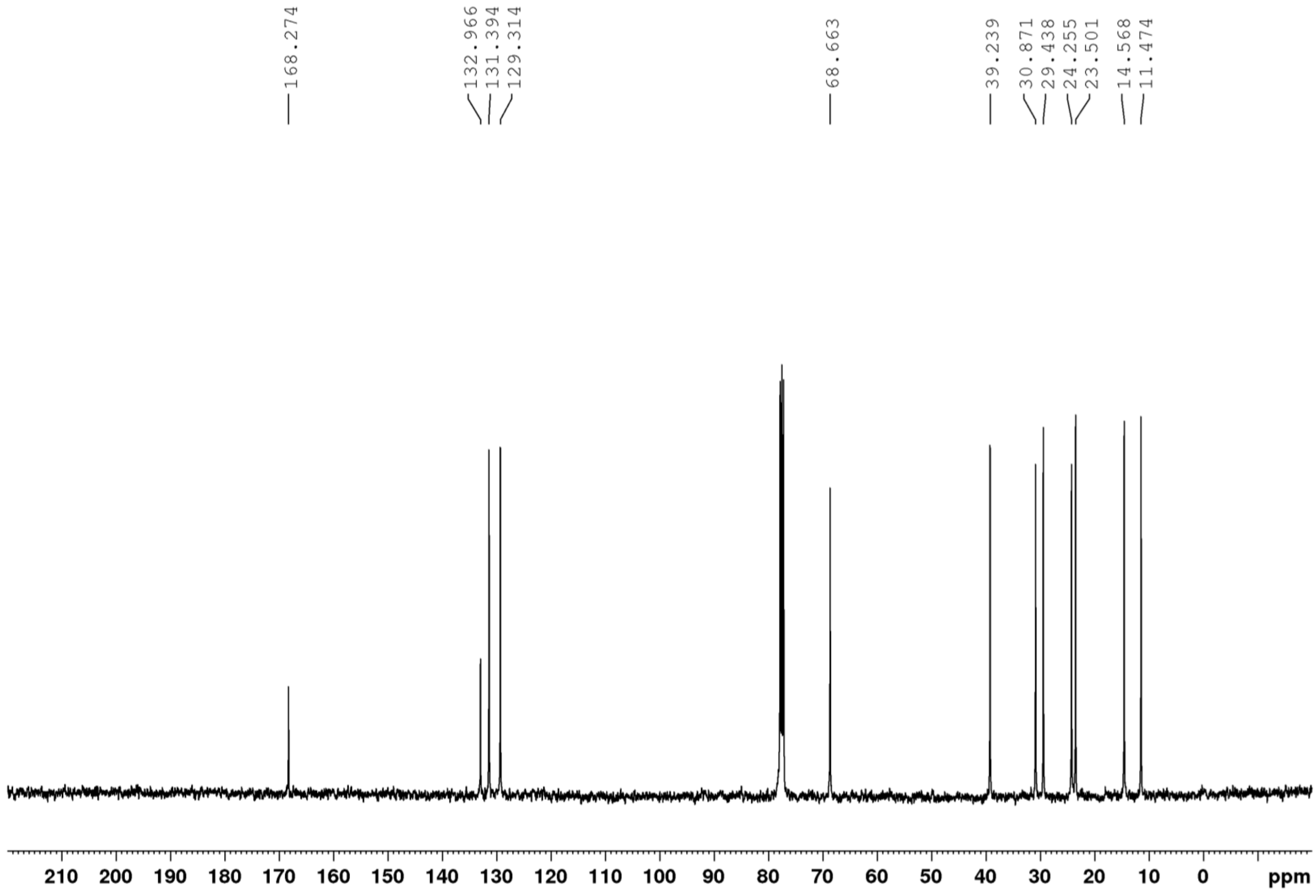

^{1}H NMR (400MHz, $CDCl_3$) of **Bis(2-ethylhexyl)[1,1'-biphenyl]-4,4'-dicarboxylate (H–H^{PL})**

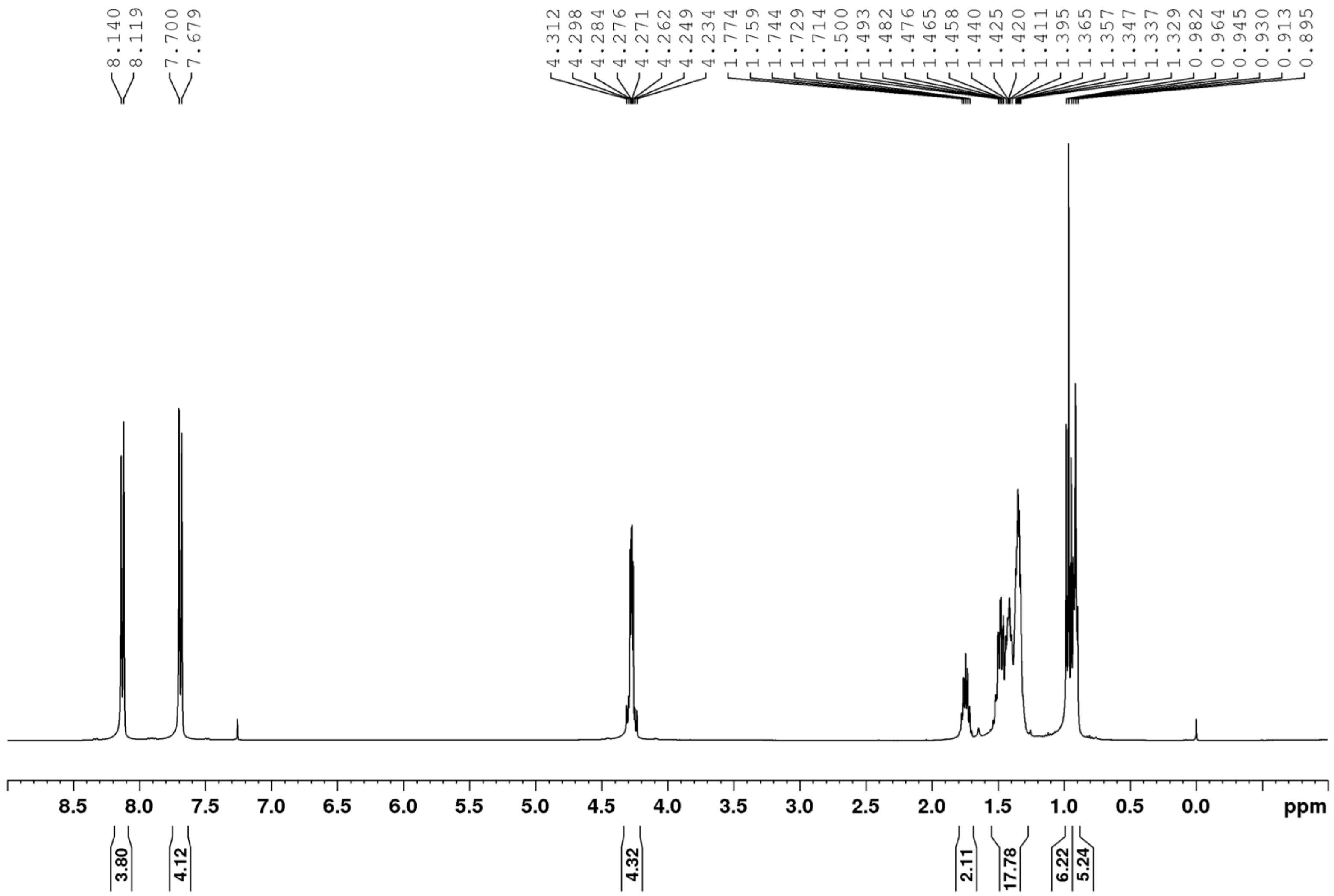

^{13}C NMR (101 MHz, $CDCl_3$) of **Bis(2-ethylhexyl)[1,1'-biphenyl]-4,4'-dicarboxylate (H–H^{PL})**

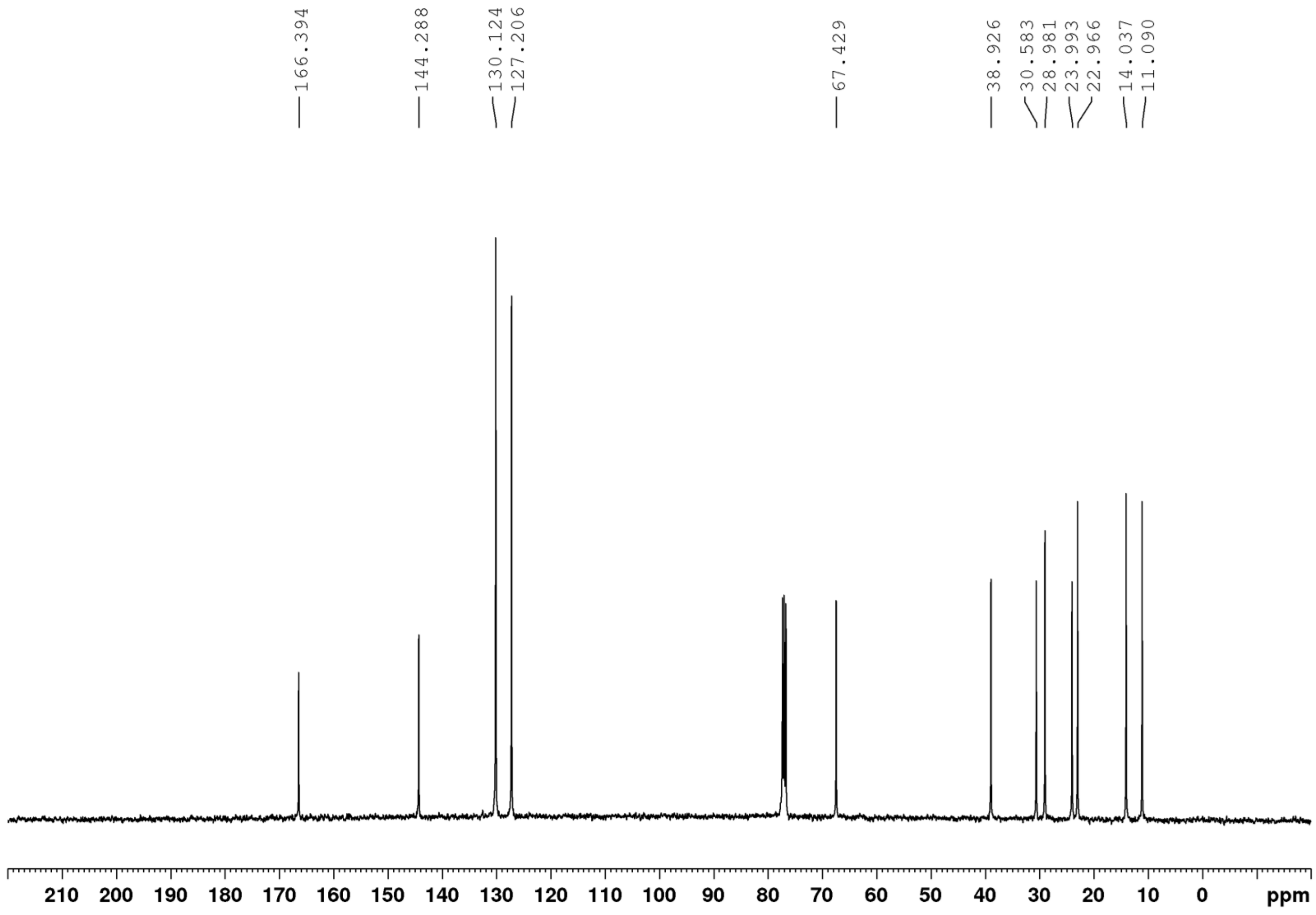

^{1}H NMR (400 MHz, $CDCl_3$) of **Bis(2-ethylhexyl) 2-methoxy-[1,1'-biphenyl]-4,4'-dicarboxylate (H–G^{PL})**

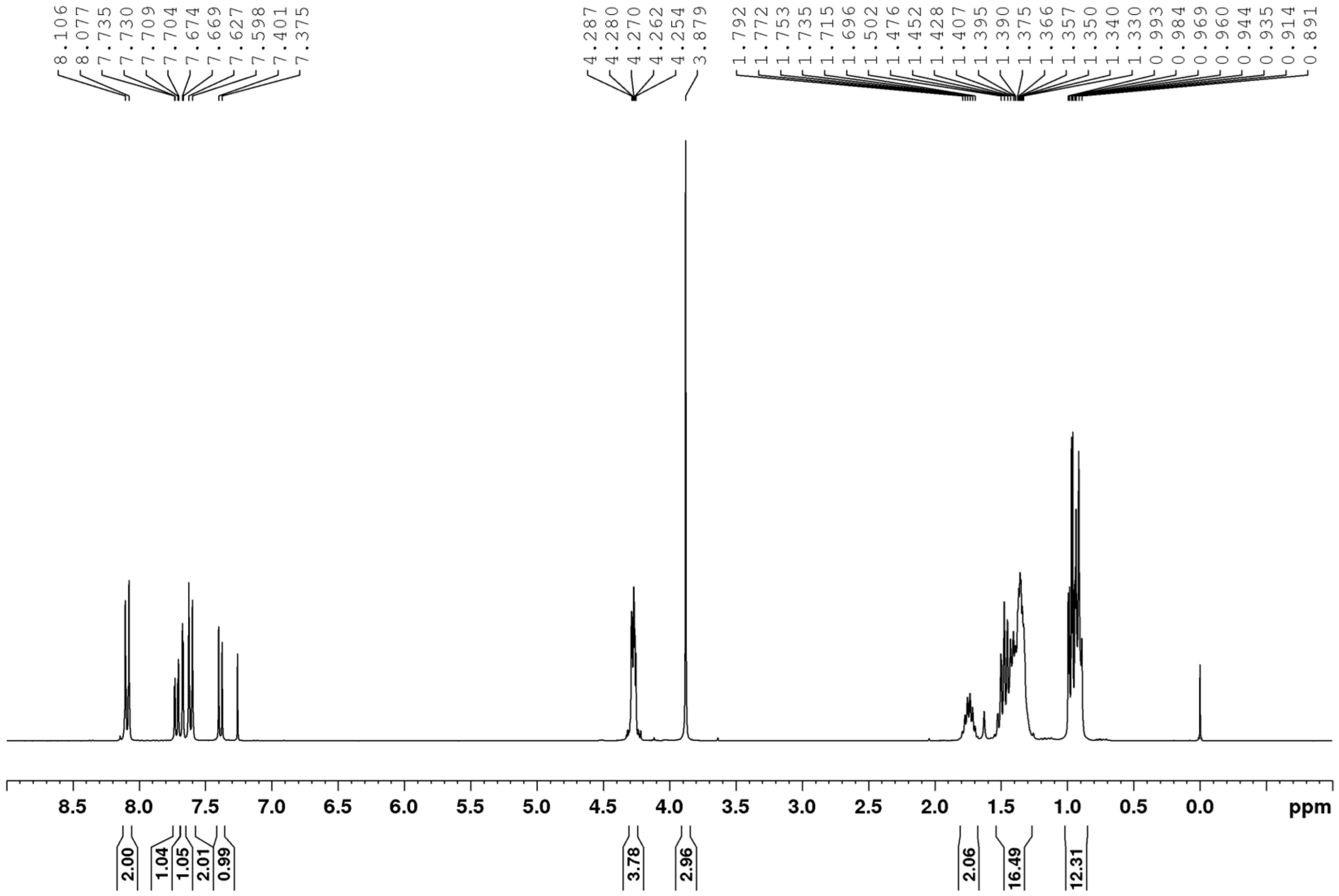

^{13}C NMR (101 MHz, $CDCl_3$) of **Bis(2-ethylhexyl) 2-methoxy-[1,1'-biphenyl]-4,4'-dicarboxylate (H–G^{PL})**

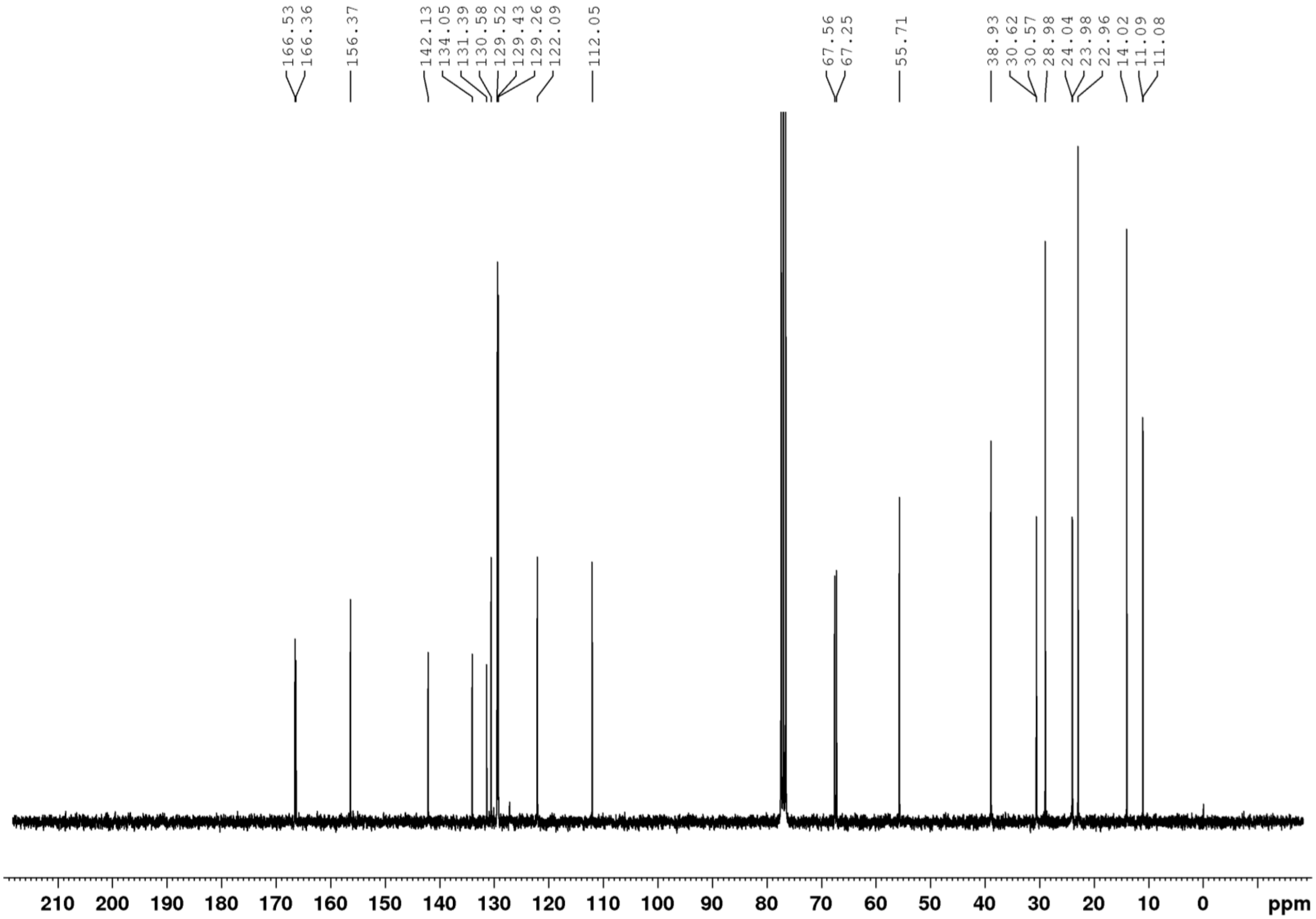

^{1}H NMR (400 MHz, $CDCl_3$) of **Bis(2-ethylhexyl) 2,6-dimethoxy-[1,1'-biphenyl]-4,4'-dicarboxylate (H–S[PL])**

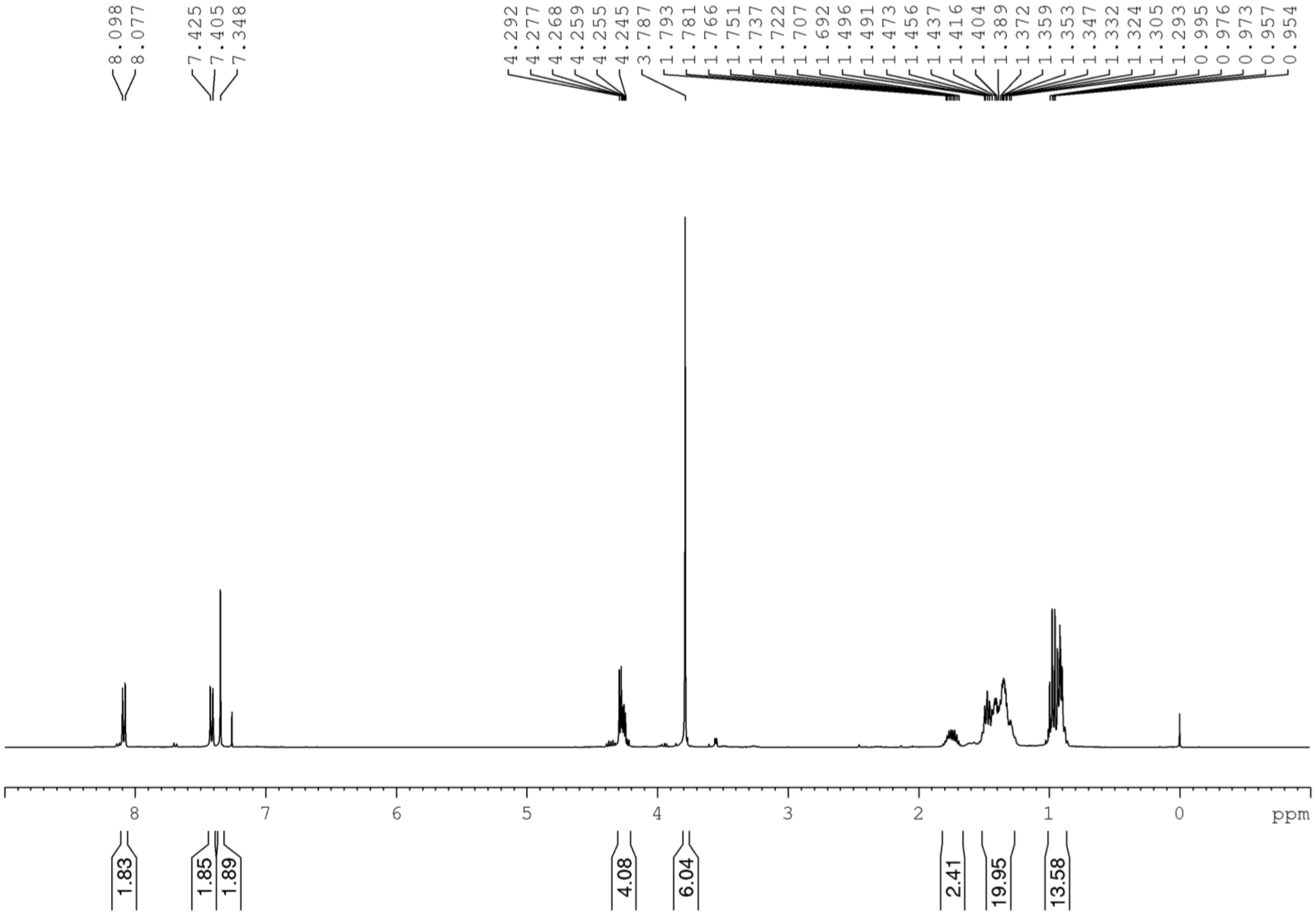

^{13}C NMR (101 MHz, $CDCl_3$) of **Bis(2-ethylhexyl) 2,6-dimethoxy-[1,1'-biphenyl]-4,4'-dicarboxylate (H–S^{PL})**

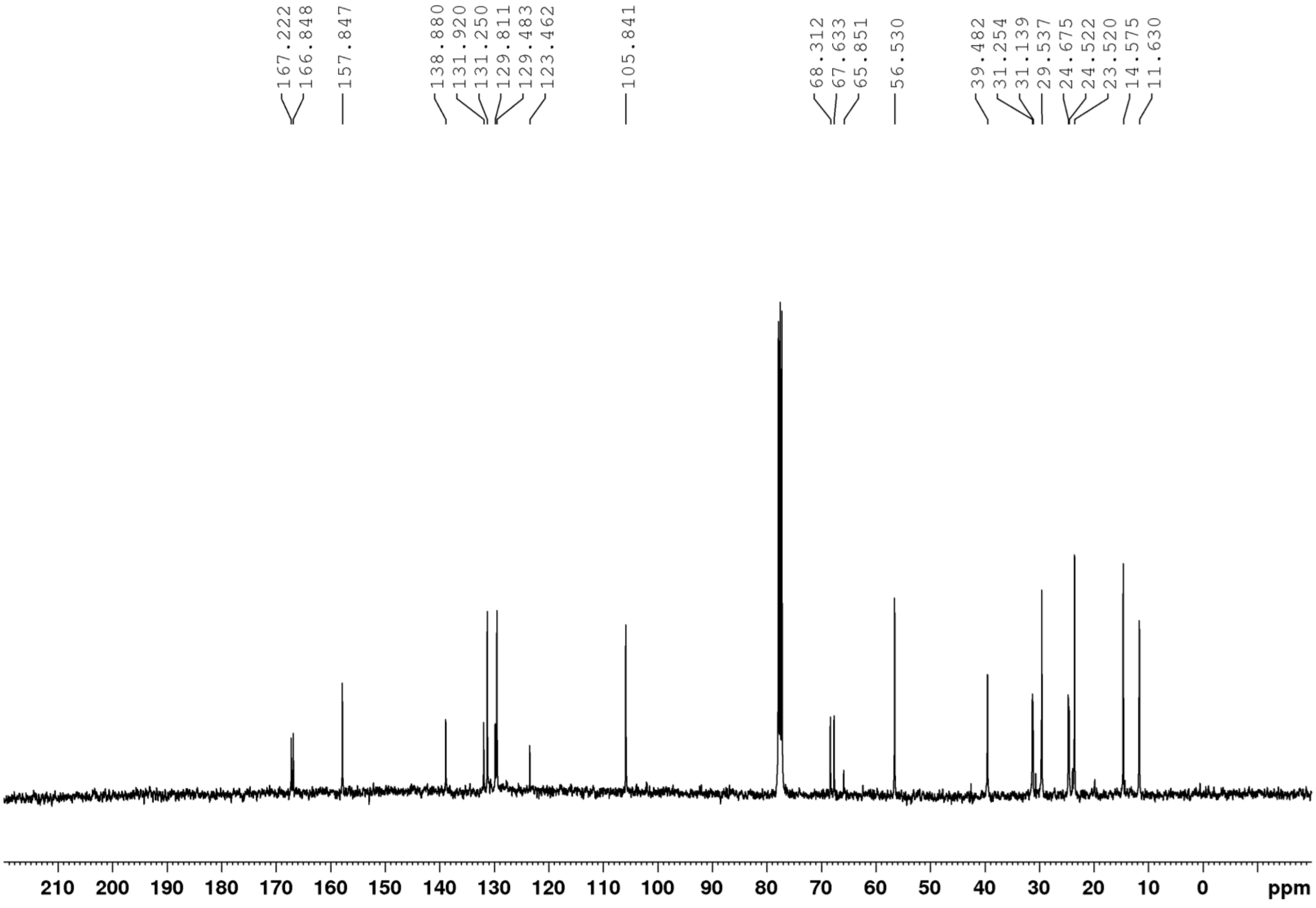

^{1}H NMR (400 MHz, $CDCl_3$) of **Bis(2-ethylhexyl) 2,2'-dimethoxy-[1,1'-biphenyl]-4,4'-dicarboxylate (G–G^{PL})**

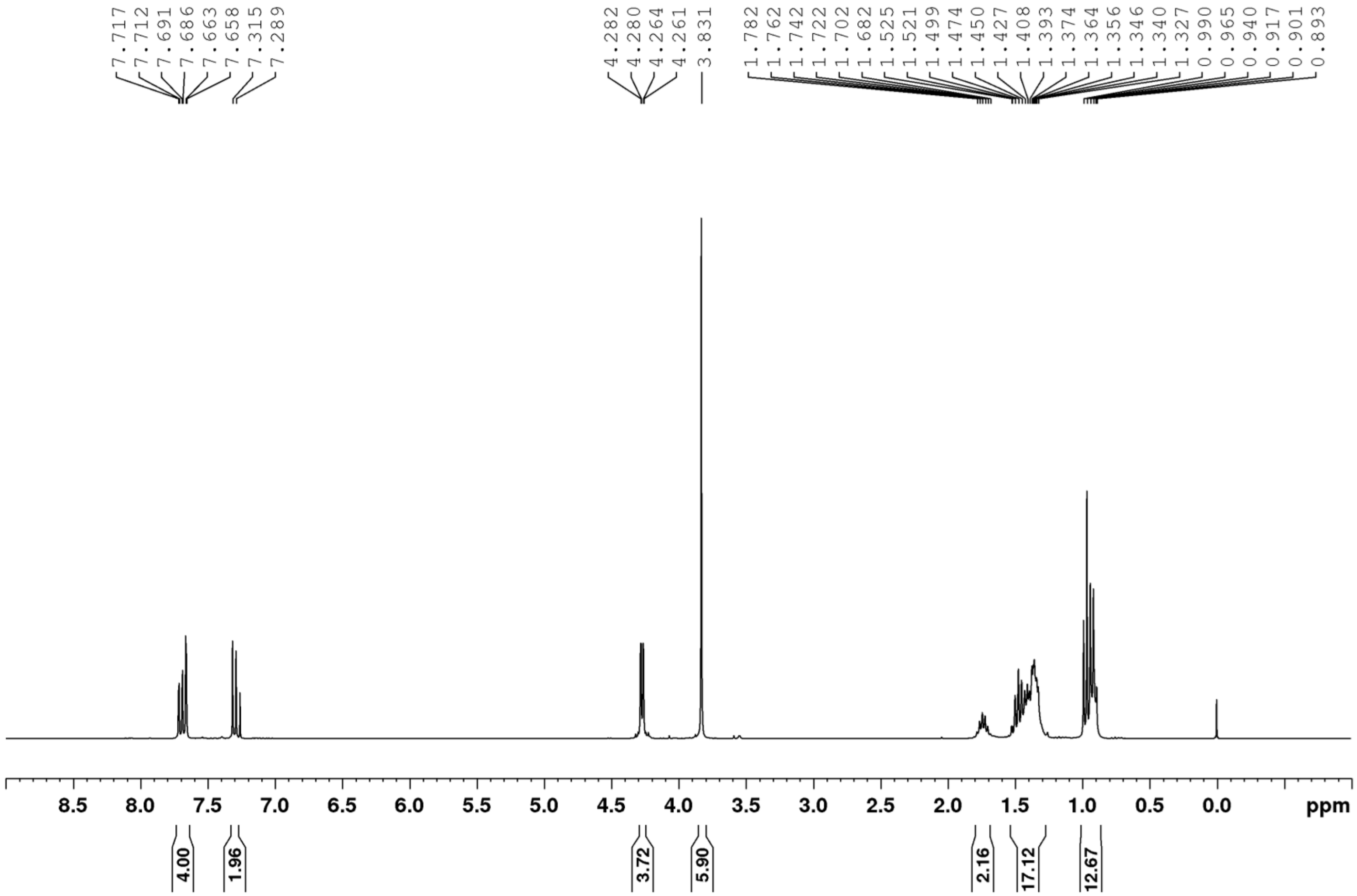

^{13}C NMR (101 MHz, $CDCl_3$) of **Bis(2-ethylhexyl) 2,2'-dimethoxy-[1,1'-biphenyl]-4,4'-dicarboxylate (G–G^{PL})**

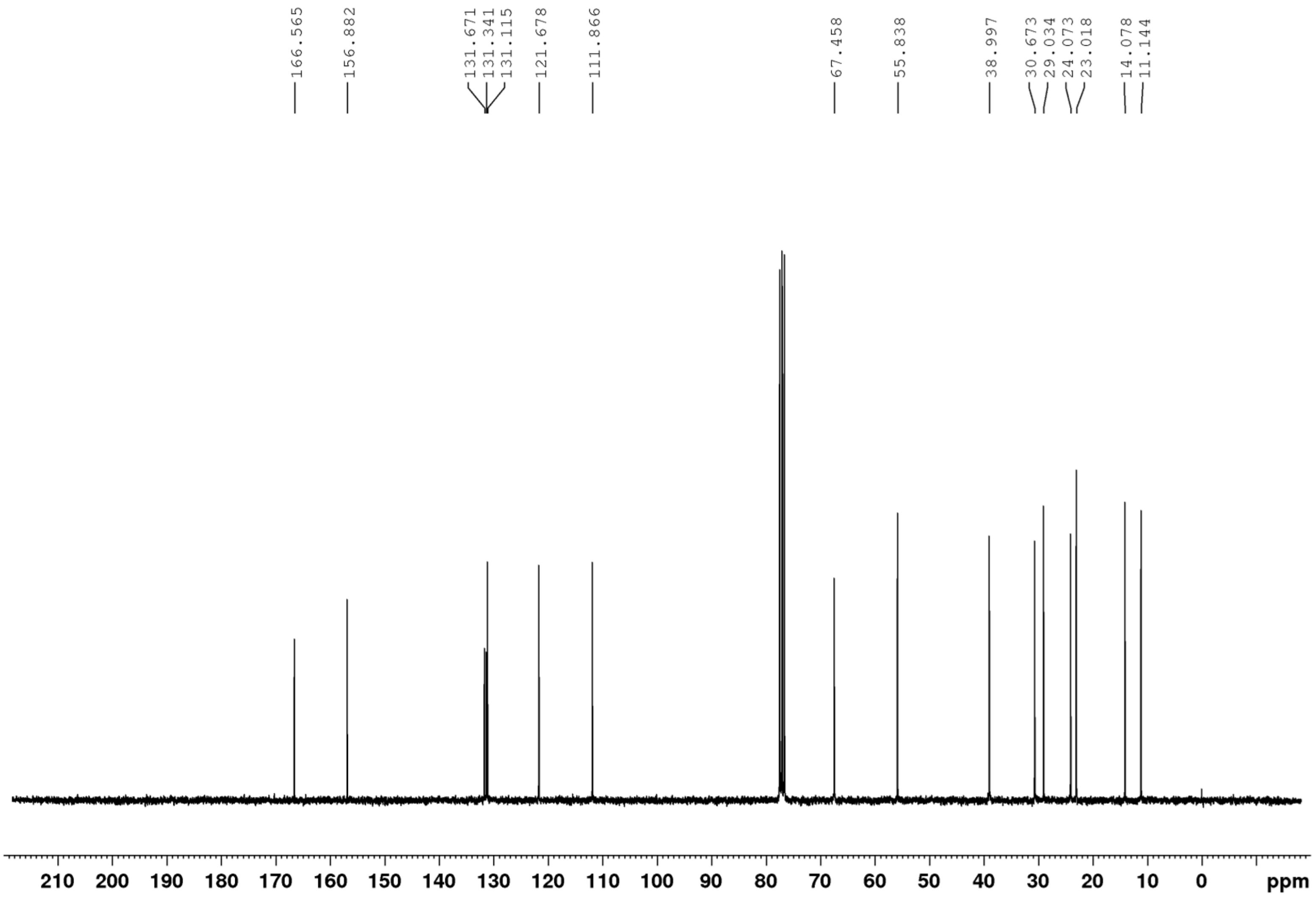

^{1}H NMR (400 MHz, $CDCl_3$) of **Bis(2-ethylhexyl) 2,2’,6-trimethoxy-[1,1’-biphenyl]-4,4’-dicarboxylate (G–S^{PL})**

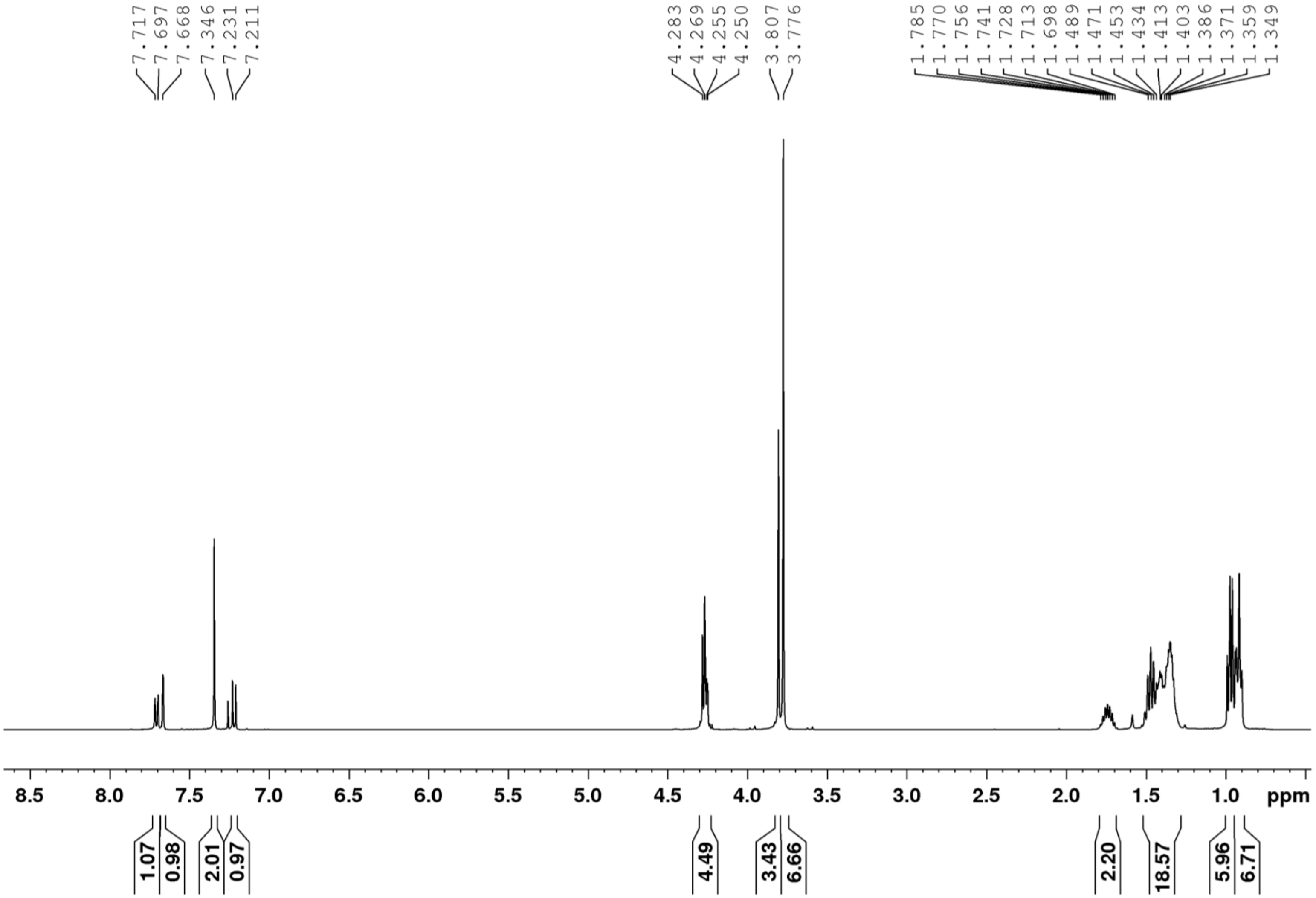

^{13}C NMR (101 MHz, $CDCl_3$) of **Bis(2-ethylhexyl) 2,2',6-trimethoxy-[1,1'-biphenyl]-4,4'-dicarboxylate (G–S^{PL})**

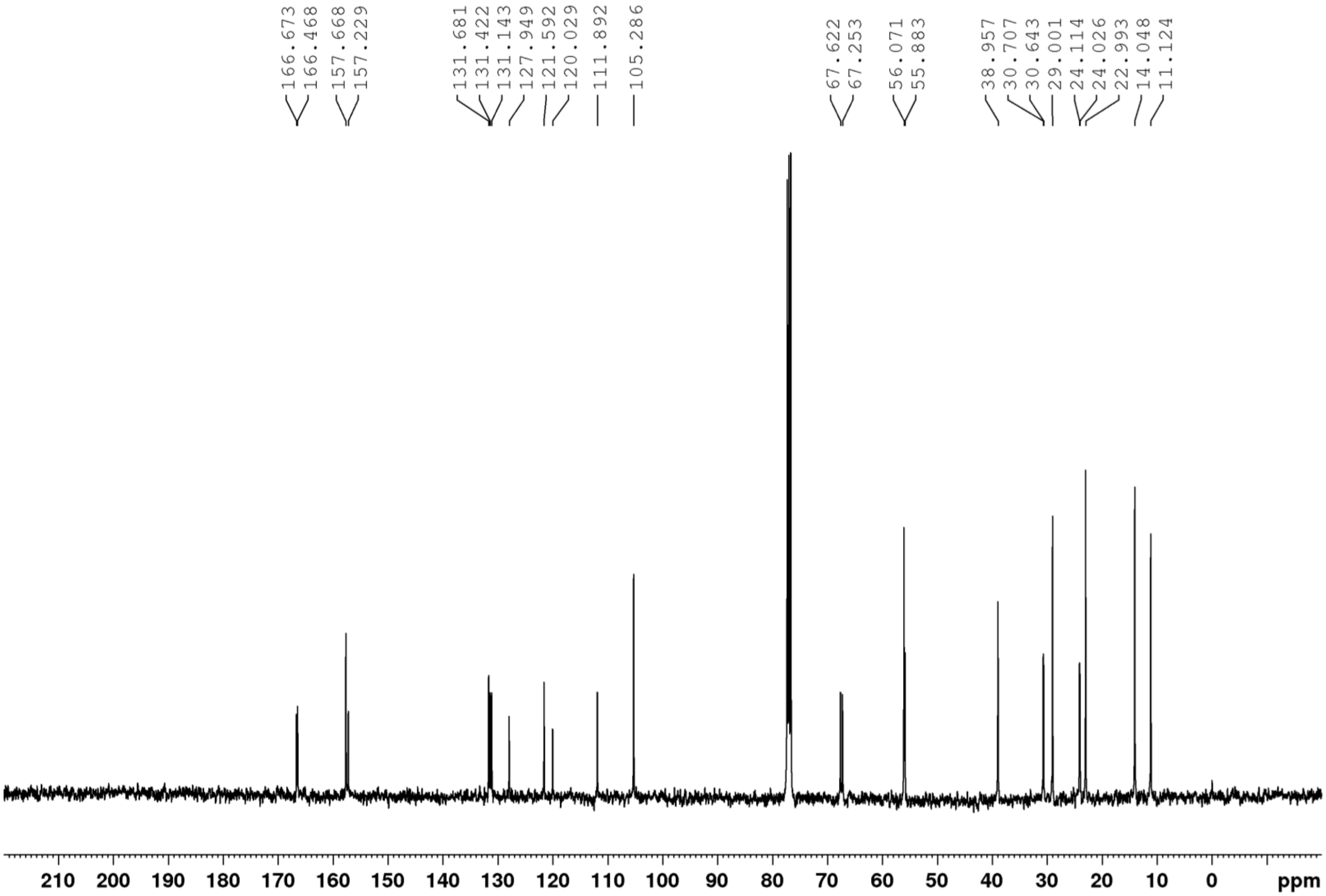

^{1}H NMR (400 MHz, $CDCl_3$) of **Bis(2-ethylhexyl) 2,2’,6,6’-tetramethoxy-[1,1’-biphenyl]-4,4’-dicarboxylate (S–S^{PL})**

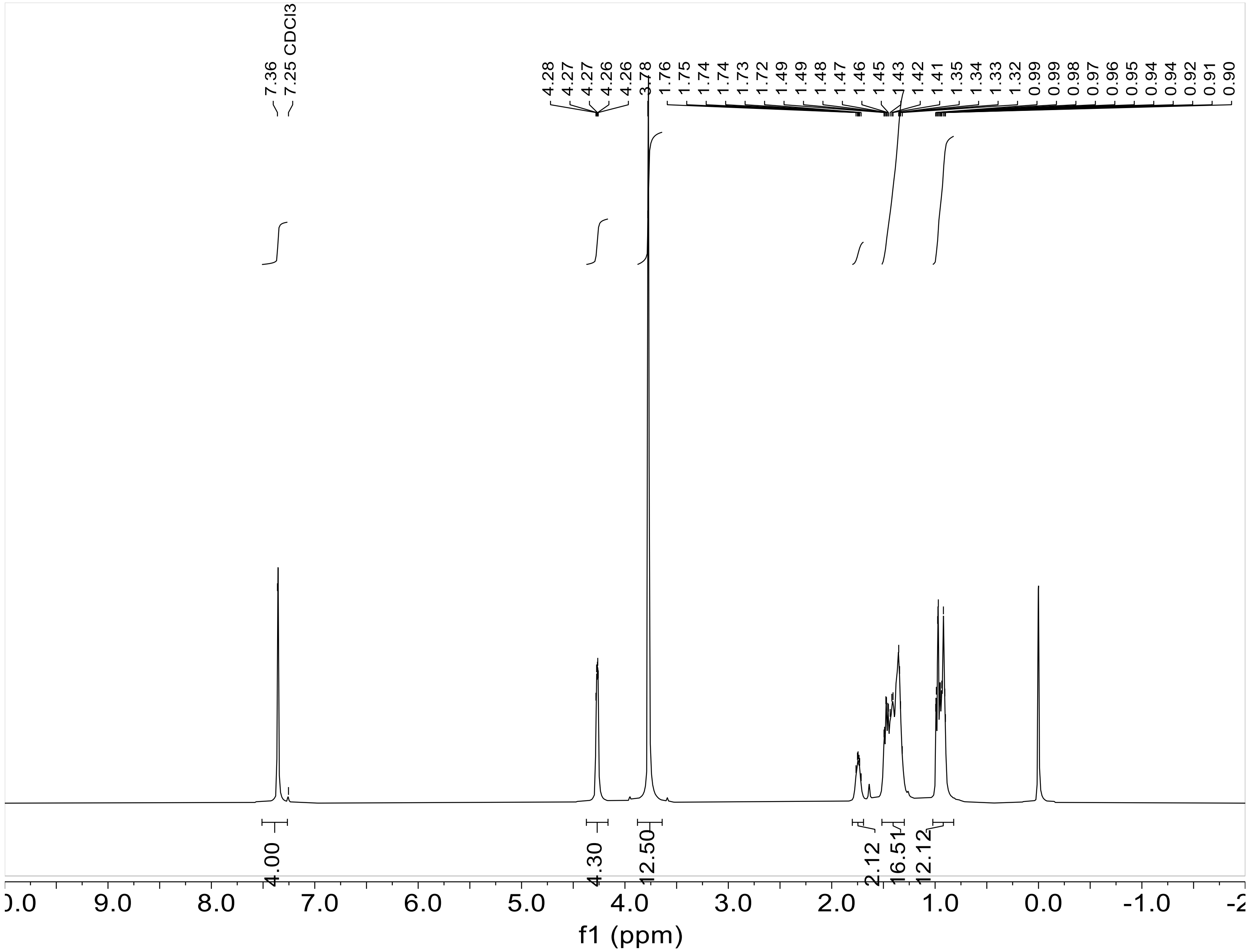

^{13}C NMR (101 MHz, $CDCl_3$) of **Bis(2-ethylhexyl) 2,2',6,6'-tetramethoxy-[1,1'-biphenyl]-4,4'-dicarboxylate (S–S^{PL})**

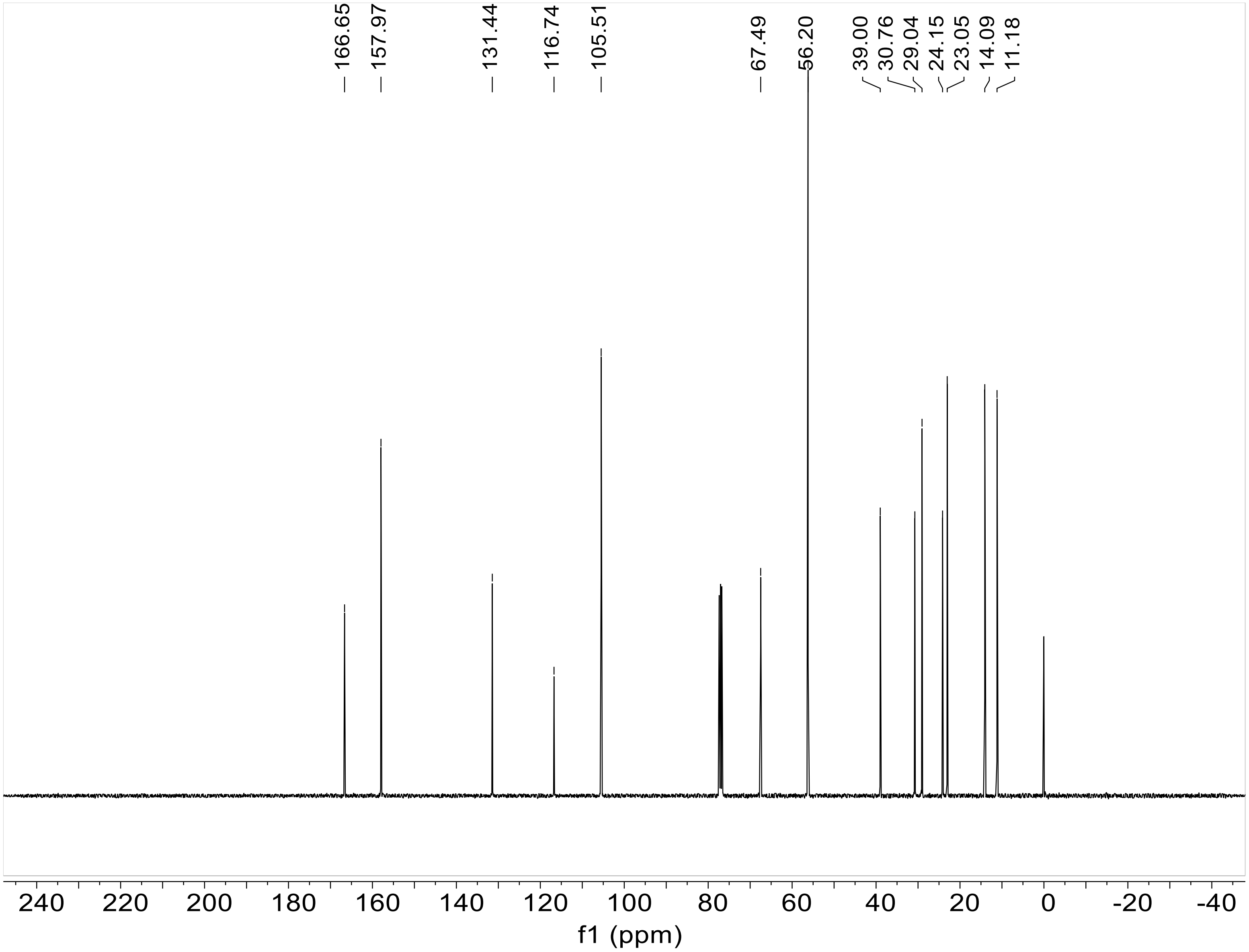

Supplement: Supplementary file 1 — oc2c01324_si_001.pdf [file oc2c01324_si_001.pdf]
